# Risk factors affecting spinal fusion: A meta-analysis of 39 cohort studies

**DOI:** 10.1371/journal.pone.0304473

**Published:** 2024-06-07

**Authors:** Shudong Yang, Beijun Zhou, Jiaxuan Mo, Ruidi He, Kunbo Mei, Zhi Zeng, Gaigai Yang, Yuwei Chen, Mingjiang Luo, Siliang Tang, Zhihong Xiao

**Affiliations:** 1 Department of Orthopedic Trauma, Second Affiliated Hospital, Hengyang Medical School, University of South China, Hengyang City, Hunan Province, China; 2 Hengyang Medical School, University of South China, Hengyang City, Hunan Province, China; 3 Department of Spine Surgery, Lishui Hospital of Wenzhou Medical University, Lishui People’s Hospital, Lishui, Zhejiang, China; 4 Department of Spine Surgery, Second Affiliated Hospital, Hengyang Medical School, University of South China, Hengyang City, Hunan Province, China; Korea University Medical Center, REPUBLIC OF KOREA

## Abstract

**Purpose:**

We performed a meta-analysis to identify risk factors affecting spinal fusion.

**Methods:**

We systematically searched PubMed, Embase, and the Cochrane Library from inception to January 6, 2023, for articles that report risk factors affecting spinal fusion. The pooled odds ratios (ORs) and 95% confidence intervals (CIs) were estimated using fixed-effects models for each factor for which the interstudy heterogeneity I^2^ was < 50%, while random-effects models were used when the interstudy heterogeneity I^2^ was ≥ 50%. Using sample size, Egger’s P value, and heterogeneity across studies as criteria, we categorized the quality of evidence from observational studies as high-quality (Class I), moderate-quality (Class II or III), or low-quality (Class IV). Furthermore, the trim-and-fill procedure and leave-one-out protocol were conducted to investigate potential sources of heterogeneity and verify result stability.

**Results:**

Of the 1,257 citations screened, 39 unique cohort studies comprising 7,145 patients were included in the data synthesis. High-quality (Class I) evidence showed that patients with a smoking habit (OR, 1.57; 95% CI, 1.11 to 2.21) and without the use of bone morphogenetic protein-2 (BMP-2) (OR, 4.42; 95% CI, 3.33 to 5.86) were at higher risk for fusion failure. Moderate-quality (Class II or III) evidence showed that fusion failure was significantly associated with vitamin D deficiency (OR, 2.46; 95% CI, 1.24 to 4.90), diabetes (OR, 3.42; 95% CI, 1.59 to 7.36), allograft (OR, 1.82; 95% CI, 1.11 to 2.96), conventional pedicle screw (CPS) fixation (OR, 4.77; 95% CI, 2.23 to 10.20) and posterolateral fusion (OR, 3.63; 95% CI, 1.25 to 10.49).

**Conclusions:**

Conspicuous risk factors affecting spinal fusion include three patient-related risk factors (smoking, vitamin D deficiency, and diabetes) and four surgery-related risk factors (without the use of BMP-2, allograft, CPS fixation, and posterolateral fusion). These findings may help clinicians strengthen awareness for early intervention in patients at high risk of developing fusion failure.

## Introduction

Spinal disease is a common clinical surgical disease, which is usually caused by lesions of the vertebral body and its surrounding soft tissue or spinal canal. Common spinal disorders include spinal degenerative diseases, inflammation, tumors, spinal deformity, and spinal fracture. These diseases can cause pain or neurological dysfunction, thus leading to a significant reduction in the patient’s quality of life and ability to work [1] In recent years, the prevalence of spinal diseases has been increasing due to the aging population [2]. Approximately 266 million people worldwide are diagnosed with symptomatic spinal degenerative disease [3].

At present, the main treatment options for spinal diseases in the clinic are conservative and surgical treatment. For most patients with acute spinal injury, early surgical treatment is needed. Conservative treatment can be chosen for early-stage chronic degenerative spinal diseases; when the effect of conservative treatment is poor or cannot achieve the desired effect, surgical treatment can be chosen again [2]. Spinal fusion has become one of the common surgical methods for spinal diseases [4], because this method can effectively eliminate pain, relieve neurological symptoms, and stabilize the spine [5, 6]. Almost 500,000 patients undergo spinal fusions annually in the United States to treat degenerative disc disease and other spinal pathologies [7]. However, fusion failure is a common adverse outcome of surgery that can cause pain, neurological symptoms, spinal deformity and reduce internal fixation stability [4].

Previous studies have reported several factors that may affect spinal fusion, such as obesity (BMI ≥ 25 kg/m^2^), smoking, graft type, vitamin D deficiency, surgical methods, and without the use of bone morphogenetic protein-2 (BMP-2). However, the results are still controversial. Niu et al.’s report suggests that patients who use BMP-2 have better fusion results than patients who do not use BMP-2 [8–13]. However, many other observational studies have not found a significant correlation between the use of BMP-2 and successful fusion [14–19]. Moreover, Zhang et al. reported that vitamin D deficiency could decrease spine fusion rates [20, 21], while Ravindra et al. found that there was no significant difference in spine fusion rates between vitamin D-deficient and non-vitamin D deficient patients [22].

To the best of our knowledge, there is no systematic review of all the risk factors that may affect spinal fusion. Therefore, we carried out a meta-analysis of risk factors reported in the literature. We also graded the evidence to better identify the risk factors affecting spinal fusion.

## Methods

### Standard protocol approvals, registrations, and patient consent

The review protocol was appropriately registered with PROSPERO (https://www.crd.york.ac.uk/prospero/) and reporting was conducted in strict accordance with guidelines from Cochrane Handbook, MOOSE (Meta-Analysis of Observational Studies in Epidemiology) [23], PRISMA (Preferred Reporting Items for Systematic Reviews and Meta-Analyses) [24] and AMSTAR (Assessing the methodological quality of systematic reviews) Guidelines [25]. The MOOSE checklist is detailed in S1 Checklist.

### Search strategy

We conducted searches on three electronic databases (PubMed, EMBASE and Cochrane Library) for English articles published prior to January 6, 2023. These studies identified the risk factors affecting spinal fusion. In instances where multiple studies reported on the same cohort, priority was given to the most recently published study or the study encompassing the largest cohort size for inclusion in our analysis. We combined "spinal fusion", "fusion rate", and "risk factors" as keywords and searched PubMed and Cochrane Library using Medical Subject Terms (MESH), and Embase databases using Embase subject heading (Embase). The search terms included ("spinal surgery" or "spinal fusion" or "joint fusion") and ("fusion rate" or "fixation rate") and ("obesity" or "electric stimulation therapy" or "smoking" or "osteoporosis" or "vitamin D") (S1 Table).

Initial retrieval of citations was processed through Endnote X9, where duplicates were merged, identified, and subsequently removed through a manual process. The preliminary assessment of the literature involved an examination of titles and abstracts to screen for relevance to our study criteria. This was followed by a meticulous independent review of the full texts of preliminarily selected studies by the research team to confirm their suitability for our meta-analysis. This rigorous selection process culminated in the inclusion of 39 studies for comprehensive analysis.

### Selection criteria

Following the preliminary article screening, two investigators independently conducted a review and verification of the articles. Any disagreements were amicably resolved through discussion or by seeking the opinion of a third evaluator. Articles were considered eligible if they satisfied the following criteria based on population, intervention, comparison, outcome, and study design (PICOS) principles

1. Population: Patients with spinal diseases who have undergone spinal fusion surgery.
2. Intervention: Assess changeable patients and possible risk factors associated with surgery, including smoking, graft type, pedicle screw type, diabetes, vitamin D deficiency, number of fused levels, fusion column, and minimally invasive surgery (MIS), without the use of BMP-2.
3. Comparison: Analyzing the differences in modifiable risk elements among subjects with or without exposure.
4. Outcome: Identifying and quantifying related risk factors through the calculation of odds ratios (ORs) and their 95% confidence intervals (CIs).
5. Study design: Prospective or retrospective cohort study.

Exclusions were applied to literature reviews, animal experiments, non-English literature, and randomized controlled trials (RCTs). Furthermore, studies lacking sufficient data were also excluded.

### Data extraction and quality assessment

Two authors extracted data using a predesigned data extraction sheet. The specific extracted content was obtained by the relevant authors by reading the full text of the article and the contents of the table. When the data was incomplete or missing, we tried to contact the corresponding author of the article to obtain the relevant data. Differences between researchers in the process of extracting data were resolved through discussion or negotiation with third parties. The following data were extracted: first author, publication year, country, type and site of surgery, observation period, sample size, study design, female proportion, mean age, measurements of fusion, mean follow-up period, significant variables.

Two authors evaluated each qualified study independently by the Newcastle‒Ottawa Scale (NOS) [26], which encompasses 3 domains, including patient representation, exposure and outcome determination, and follow-up adequacy, with an overall score of 9 for each study. The NOS scores were then stratified into three qualitative tiers reflecting study quality: low (0–5 points), moderate (6–7 points), and high (8–9 points, indicative of a minimal bias risk) [27]. The quality evaluation results of the study included in this meta-analysis are shown in S2 Table.

### Evaluation of the strength of evidence

The grading of the strength of evidence in the identified associations for observational cohort studies was conducted utilizing a set of modified criteria [28]. When the P value of Egger’s test was greater than 0.05, the total sample size was over 500, and interstudy heterogeneity I^2^ was less than 50%, the association was deemed high-quality (class I) evidence. If two out of the three conditions were satisfied, the association was classified as class II (medium-quality) evidence. Meeting one of these three conditions resulted in a class III (medium-quality) evidence correlation. Failure to meet any of these three conditions indicated class IV (low-quality) evidence (S3 Table).

### Statistical analysis

All of our analyses were performed using Stata software (Stata version 16.0, College Station, Texas, USA). We analyzed the risk factors affecting spinal fusion, including patient-related factors (e.g., smoking, diabetes, and vitamin D deficiency). surgery-related risk factors (e.g., allograft, without the use of BMP-2, conventional pedicle screw (CPS) fixation, and posterolateral fusion, MIS, number of fused levels). The odds in each group were computed as *p/(1-p)* where *p* represents the proportion with exposure. The odds ratio (OR) was determined by dividing the odds in the fusion failure group by the odds in the comparator group. In the meta-analyses, study-specific log odds ratios were utilized as the outcome, and the aggregated estimates were then transformed into OR. If the OR > 1, it indicates a higher probability of fusion failure in the exposed group as opposed to the non-exposed group. Forest plots were utilized to present the ORs of individual studies as well as the pooled OR. The heterogeneity between studies was determined using the Cochrane Q test and I^2^ test, with heterogeneity considered significant when I^2^ > 50% [29]. Sensitivity analyses were conducted to evaluate the robustness of the findings by systematically excluding individual studies and subsequently pooling the estimates from the remaining studies through meta-analysis. Egger’s test was used to evaluate publication bias for each risk factor by analyzing the relationship between the effect estimates and their variances. A P value of < 0.1 was deemed to signify a significant distinction [30]. All statistical tests were bidirectional, and *P* < 0.05 was considered statistically significant.

## Results

### Literature search and study characteristics

**Fig 1:**
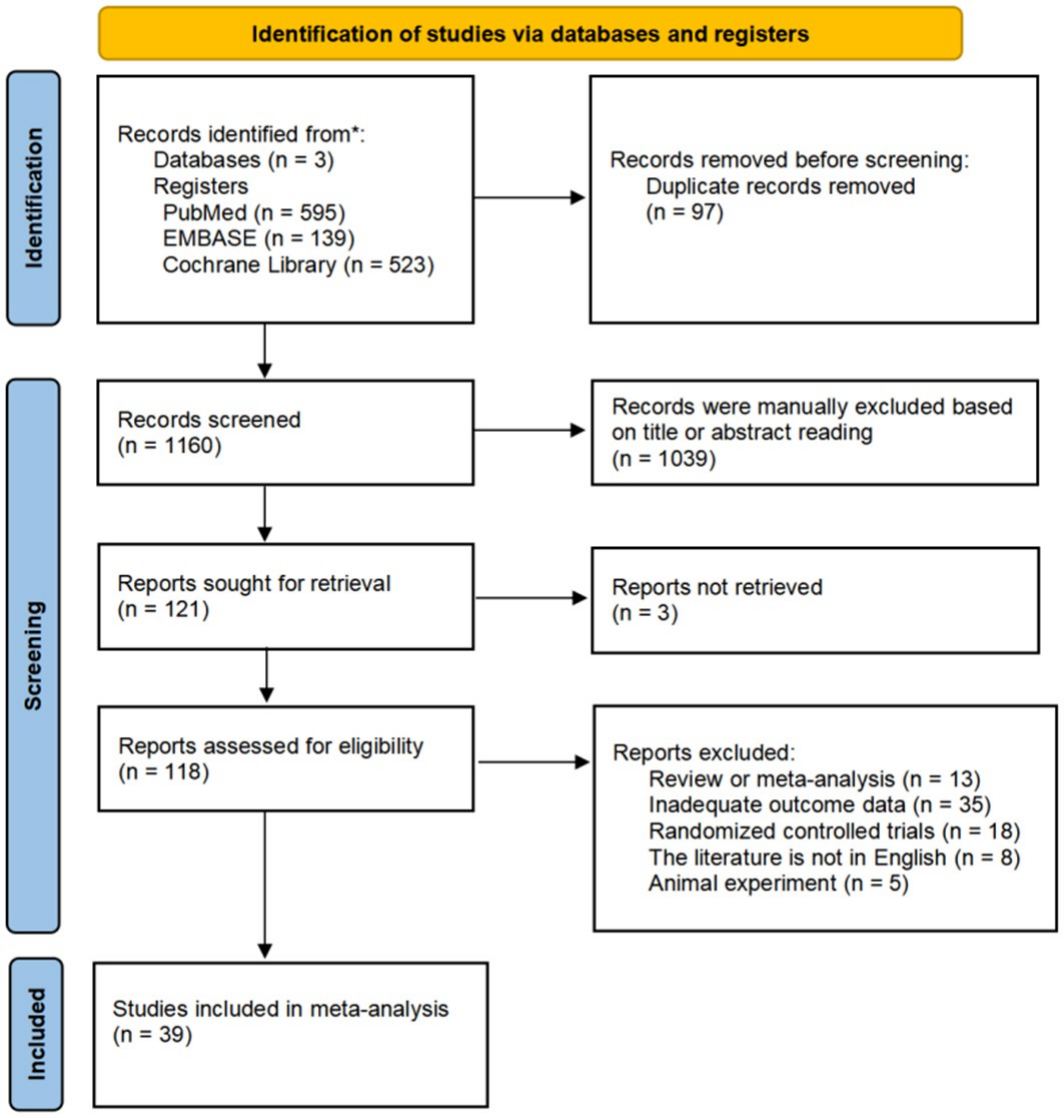
Flowchart of the study selection.

Out of 1,257 studies identified through a systematic literature search, 97 duplicate records were excluded, and 1,039 irrelevant studies were excluded after reviewing their titles and abstracts. Next, we excluded 3 citations for which three could not be obtained in full-text form, and 118 studies were selected for review of the full paper. After a full-text review, we excluded 79 studies that did not have access to patient outcome data, non-population-based cohorts, meta-analyses, systematic reviews, RCTs, and non-English literature (S4 Table). Finally, this meta-analysis included 39 cohort studies [8–22, 31–54], comprising 7,145 participants satisfied the inclusion criteria (Fig 1).

Table 1 displays the baseline characteristics of the studies included in the analysis. All studies were published between 1996 and 2022, and 24 (60%) of the studies were published in 2013 or later [9, 12–15, 17, 19–22, 37–41, 43, 45, 46, 48–51, 53, 54]. The studies involved 11 countries with an average sample size of 183, and the average follow-up time was 31 months (range 6–183 months). Out of the studies analyzed, 30 (76.9%) studies achieved an NOS score of ≥ 8 (S2 Table) [9–12, 14–16, 20–22, 32–38, 40–42, 44–49, 51–54]. In 25 (64%) [10–13, 15–21, 34–37, 39–41, 43, 46, 48, 49, 51, 53, 54] studies, computed tomography (CT) scans were used to assess fusion. The standards for spinal fusion were defined in 27 (69%) studies [10, 15–22, 31, 32, 34, 37–39, 41–44, 46–50, 52–54].

**Table 1:** Characteristics of studies included in meta-analysis.

**First author**	**Year**	**Country**	**Observation Period**	**Study Design**	**Sample size**	**Female %**	**Mean age**	**Measurements of fusion**	**Mean Follow-up period**
Glassman, S. D.	2000	USA	1992–1996	Retrospective	357	43.0	43.9	3D-CT	24 months
Bose, B.	2001	USA	NR	Retrospective	106	55.7	50.1	X-ray	≥12 months
Samartzis, D.	2003	USA	NR	Retrospective	80	36.3	49.0	Radio graphs	16 months
Glassman, S. D.	2003	USA	NR	Retrospective	137	60.5	60.3	CT	12 months
Gerszten, P. C.	2011	USA	2005–2007	Retrospective	99	42.8	42.8	MRI and CT	24 months
Hoffmann, M. F.	2012	USA	2003–2009	Retrospective	1398	58.9	60.0	CT and X-ray and MRI	183 months
Luszczyk, M.	2013	USA	NR	Retrospective	573	NR	NR	Radio graphs	24 months
Urrutia, J.	2013	Chile	2004–2010	Retrospective	47	76.6	46.5	CT	50.1 months
Frenkel, M. B.	2013	USA	1997–2012	Retrospective	45	57.7	50.0	X-ray and CT	161 months
Adams, C. L.	2014	Australia	2007–2010	Retrospective	70	50.0	55.4	Radio graphical	12 months
Tan, B.	2015	China	2007–2010	Retrospective	146	41.1	64.7	Radio graphs	24 months
Yang, Y.	2016	China	2011–2014	Retrospective	132	32.6	44.2	CT	24 months
Zhang, Y. H.	2017	China	2007–2015	Retrospective	32	NR	6.80	CT	45 months
Phan, K.	2018	Australia	NR	Retrospective	137	52.6	56.7	CT	12 months
Weng, F.	2018	China	2015–2017	Retrospective	80	71.3	65.6	NR	6 months
Nourian, A. A.	2019	USA	NR	Retrospective	93	67.0	65.0	CT	>12 months
Niu, S.	2020	NR	2007–2017	Retrospective	927	59.0	63.8	CT	6 months
Wu, F. L.	2020	China	NR	Retrospective	50	48.0	61.2	MRI and CT	30 months
Son, H. J.	2021	USA	2009–2019	Retrospective	121	76.0	68.2	CT	>12 months
Tan, Y.	2021	Japan	2017–2020	Retrospective	98	64.3	69.5	CT	33.5 months
Tannoury, C.	2021	USA	2008–2019	Retrospective	220	62.8	66.0	CT	12 months
Wang, H.	2021	China	NR	Retrospective	153	66.8	50.1	CT	40.8 months
Li, Z.	2022	China	2017–2019	Retrospective	77	52.0	44.6	CT	12 months
Bishop, R. C.	1996	USA	1991–1995	Prospective	132	54.6	45.2	X-ray	31 months
Tuli, S. K.	2004	USA	1995–1999	Prospective	57	47.4	52.2	Radio graphs	6 months
Suchomel, P.	2004	Czech	1998–2000	Prospective	79	37.9	47.8	X-rays	48 months
Burkus, J. K.	2004	USA	NR	Prospective	46	NR	NR	Radio assessments	24 months
Cammisa Jr, F. P.	2004	USA	NR	Prospective	120	49.0	48.0	X-ray	24.5 months
Burkus, J. K.	2005	USA	1998–2001	Prospective	131	61.1	41.5	Radio graphs and CT	≥24 months
Joseph, V.	2007	Canada	2003–2005	Prospective	33	39.4	49.7	CT	25 months
Frantzén, J.	2011	Finland	1996–1998	Prospective	17	70.5	49.4	CT and X- ray and MRI	132 months
Wu, Z. X.	2012	China	2004–2009	Prospective	157	61.2	62.1	Radio graphs	43 months
Ravindra, V. M.	2015	USA	2011–2012	Prospective	133	44.0	57.0	CT	>12 months
Burkus, J. K.	2017	USA	NR	Prospective	710	NR	NR	X-ray	24 months
Moazzeni, K.	2018	Iran	2014–2015	Prospective	96	62.5	57.8	CT and X-ray	12 months
Ravindra, V. M.	2019	USA	2011–2012	Prospective	58	41.4	57.1	X-ray	≥12 months
Srour, R.	2020	France	2017–2018	Prospective	53	50.9	65.0	CT	12 months
Hyun, S. J.	2021	Korea	NR	Prospective	76	44.7	63.4	X-ray and CT	12 months
Zhang, W.	2022	China	2018–2020	Prospective	69	61.5	54.6	CT	6 months
**First author**	**Year**	**Fusion definition**		**significant variables**	**Fusion rate (%)**		**Surgical sites**	**Surgical types**	
Glassman, S. D.	2000	NR		Smoking	Smokers: 79.3%, Nonsmokers: 85.8%		Lumbar	Posterior fusion	
Bose, B.	2001	Trabecular bony bridging across the disk space and lack of motion on flexion extension.		Smoking	Nonsmoking: 96.67%, smoking: 97.83%		Cervical	ACDF	
Samartzis, D.	2003	A bony bridge incorporated the graft and the adjacent end plates and no radiolucencies or motion.		Autograft, allograft	Allograft: 94.3%; autograft: 100%		Cervical	ACDF	
Glassman, S. D.	2003	NR		IDDM, NIDDM	NIDDM: 78.26%/IDDM: 74.28%, Control: 94.59%		Lumbar	PLIF	
Gerszten, P. C.	2011	Bridging bone that traversed from end plate to end plate, with no motion detected on flexion extension lateral radio graphs.		rhBMP-2	With rhBMP-2: 95.5%, Without rhBMP-2: 92.5%		Lumbar	Interbody fusions in the lumbosacral spine	
Hoffmann, M. F.	2012	NR		DBM with rhBMP-2	rhBMP-2: 95.7%/DBM: 86.9, Autograft: 84.8%		Lumbar	PLIF, TLIF	
Luszczyk, M.	2013	Bony trabecular bridging between the graft and vertebral body, and motion was absent.		Smoking	Smoking: 91%, control: 91.6%		Cervical	ACDF	
Urrutia, J.	2013	Trabeculae crossing the graft-vertebral body interface on both sides of the graft either.		Smoke	Smoke: 91.7%		Lumbar	Circumferential lumbar spinal fusion	
Frenkel, M. B.	2013	The presence of motion on flexion-extension radio graphs.		rhBMP-2	With rhBMP-2: 100%, Without rhBMP-2: 83%		Cervical	Anterior cervical fusion	
Adams, C. L.	2014	NR		rhBMP-2, LBC	rhBMP-2: 94.1%, LBC: 89.5%		Lumbar	PLIF or TLIF	
Tan, B.	2015	NR		rhBMP-2, ICBG	rhBMP-2: 87.7%, ICBG: 74%		Cervical	ACDF	
Yang, Y.	2016	Evidence of continuous bridging bone between the adjacent end plates of the involved motion segment, radiolucent lines at 50% or less of the graft vertebra interfaces.		Two-level or single level ACDF	Single level: 94.6%, Two-level: 92.7%		Cervical	ACDF	
Zhang, Y. H.	2017	The lack of hardware failure and presence of continuous bridging trabecular bone between the dorsal elements of the C1 and C2 on CT scans.		Structural allograft or autograft	Allograft: 94%, autograft: 100%		Cervical	Atlantoaxial fusion	
Phan, K.	2018	Bridging trabecular formation across the intervertebral disk space with the absence of radiolucency spanning more than half of the implant.		Smoking	Smoking: 69.6%, no smoking: 85.1%		Lumbar	ALIF	
Weng, F.	2018	Complete fusion and remodeling after intervention with newly-formed trabeculae; complete bone block after intervention.		EPS, CPS	EPS: 90%, CPS: 50%		Lumbar	Lumbar short-segment fixation and fusion	
Nourian, A. A.	2019	NR		LLIF with rhBMP-2	1-level LLIF with rhBMP-2: 92%, 2-level LLIF with rhBMP-2: 86%		Lumbar	LLIF	
Niu, S.	2020	NR		RhBMP-2	RhBMP-2: 92.5%, no rhBMP-2: 71.4%		Lumbar	TLIF	
Wu, F. L.	2020	NR		MIDLF, MI-TLIF	MIDLF: 94%, MI-TLIF: 88%		Lumbar	MIDLF, MI-TLIF	
Son, H. J.	2021	Lenke grade B and BSF grade-3.		ICBG, E.BMP-2	ICBG: 97.1%, E.BMP-2: 100%		Lumbar	LIF and PLF	
Tan, Y.	2021	NR		Simultaneous single-position O-arm-navigated OLIF and PPS, MI-PLIF/TLIF	OLIF and PPS: 96.8%, MI-PLIF/TLIF: 94.2%		Lumbar	MIS-ATP-lumbar fusions	
Tannoury, C.	2021	Examining consecutive sagittal and coronal cuts for continuous bony bridges.		Smoke, L5-S1	Smoke: 95.3%, L5-S1: 95.2%		Lumbar	OLIF and PPS, MI-PLIF/TLIF	
Wang, H.	2021	Mental ROM less than 3° in X-ray and continuous bone bridge demonstrated in CT imaging.		Male, smoke	Smoke: 87.84%		Cervical	CDR and ACDF	
Li, Z.	2022	Unilateral or bilateral grade I or II fusion.		Modified facet joint fusion or posterolateral fusion	MFF: 94.3%, PLF: 76.2%		Lumbar	Modified facet joint fusion or posterolateral fusion	
Bishop, R. C.	1996	Bony trabeculae were seen crossing the involved interspace.		Autograft tricortical iliac and the allograft tricortical iliac	Single-level ACDF: (autograft: 97%, allograft: 87%), multiple level interbody fusion: (autograft: 100%, allograft: 89%)		Cervical	Single-level or multiple-Level ACDF	
Tuli, S. K.	2004	Presence of any trabeculae bridging between the vertebral body and allograft at the upper and lower aspects.		One level or two-level corpectomy	Cephalad aspect of the graft-host interface: 92%, caudad aspect: 93%, one-level corpectomy: 86%, two-level corpectomy: 100%		Cervical	Cervical decompressive corpectomy and reconstruction	
Suchomel, P.	2004	Complete bridging of trabeculae between adjacent vertebral bodies and bone graft.		Number of fused levels, smoke, autologous, allogenic bone grafts	Autografts: 94.6%, allografts: 85.5%, smoke: 92.4%		Cervical	One- or two-level ACDF	
Burkus, J. K.	2004	NR		rhBMP-2, ICBG	rhBMP-2-treated: 100%, auto graft-treated: 68.4%		Lumbar	ALIF	
Cammisa Jr, F. P.	2004	NR		Grafton^®^, auto graft	Grafton^®^ side: 52%, auto graft side: 54%		Lumbar	Posterolateral spine fusion	
Burkus, J. K.	2005	(1) The presence of bridging trabecular bone connecting vertebral bodies through or around dowels, (2) no radiolucent area involving > 50% of the interface between dowels and end plates.		rhBMP-2/ACS, ICBG	rhBMP-2-treated: 98.5%, autograft-treated: 76.1%		Lumbar	Single-level ALIF	
Joseph, V.	2007	The presence of bridging bone through the cage or external to it.		rhBMP-2	With BMP: 94.4%, Without BMP: 89.4%		Lumbar	PLIF and TLIF	
Frantzén, J.	2011	Bridging of bone between the transverse processes in addition to the incorporation of bone between the transverse processes.		BAG	BAG: 90%, Autologous Bone: 100%		Lumbar	Posterolateral spondylodesis	
Wu, Z. X.	2012	Clear trabecular bone bridging across the segment to be fused, translation of 3 mm or less and angulation of < 5° on flexion-extension radio graphs.		EPS, CPS	EPS: 92.5%, CPS: 80.5%		Lumbar	Transpedicle fixation	
Ravindra, V. M.	2015	The presence of bone trabeculation, without evidence of instrumentation loosening or breakage, and no observed motion between the graft and instrumentation.		Vitamin D	84%		spine	Spinal fusion	
Burkus, J. K.	2017	NR		rhBMP-2, ICBG	rhBMP-2-treated: 99.4%, autograft-treated: 87.2%		Cervical	Single-level anterior cervical arthrodesis	
Moazzeni, K.	2018	Bridging bone remodeling across the transverse processes between the adjacent vertebrae.		Diabetic and non-diabetic patients after lumbar fusion	DM: 53%, control: 78%		Lumbar	Bilateral facet fusion	
Ravindra, V. M.	2019	The presence of trabeculated bone, without evidence of hardware loosening or failure, and no observed motion between vertebral segments on X-rays.		Low vitamin D	Normal vitamin D: 76.47%, low vitamin D: 75.60%		Cervical	Anterior, posterior, or combined spinal fusion	
Srour, R.	2020	Any sign of bony fusion inside or posterior to the device when viewing the postoperative CT scan.		Facet arthrodesis with or without PLIF	Facet arthrodesis with PLIF: 75.7%, non-PLIF: 88.7%		Lumbar	Facet osteosynthesis	
Hyun, S. J.	2021	Less than 5 degrees of angular motion on flexion and extension radio graphs.		rhBMP-2	DBM with rhBMP-2: 82.85%, DBM: 78.12%		Lumbar	TLIF	
Zhang, W.	2022	Bridging bone bonding with both adjacent vertebral bodies.		Vitamin K2 + Vitamin D3	Vitamin K2 + Vitamin D3: 91.18%, control: 71.43%		Lumbar	TLIF or PLIF	

Abbreviations: ACDF = Anterior cervical discectomy and fusion; ACS = absorbable collagen sponge; ALIF = Anterior lumbar interbody fusion; BAG = bioactive glass; BMI = body mass index; BSF = Brantigan, Steffee and Fraser; CDR = Cervical disc replacement; CPS = conventional pedicle screws; CT = computed tomography; DBM = demineralized bone matrix; DM = diabetes mellitus; E.BMP-2 = E.coli-derived rhBMP-2; EPS = expandable pedicle screws; HA = hydroxyapatite; HO = heterotopic ossification; ICAG = iliac crest autograft; ICBG = iliac crest bone graft; IDDM = insulin-dependent diabetes mellitus; JOA = Japanese Orthopedic Association; LBC = local bone graft; LBP = low back pain; LLIF = Lateral lumbar interbody fusion; MFF = modified facet joint fusion; MIDLF = midline lumbar fusion; MI-PLIF/TLIF = Minimally invasive posterior or transforaminal lumbar interbody fusion; MIS-ATP = minimally invasive antepsoas; MI-TLIF = minimally invasive transforaminal lumbar interbody fusion; MRI = magnetic resonance imaging; NDI = Neck Disability Index; NIDDM = non-insulin dependent diabetes mellitus; NR = not reported; OLIF and PPS = Oblique lateral interbody fusion and percutaneous pedicle screw; OP-1 = Osteogenic Protein-1; PLF = posterolateral fusion; PLIF = posterior lumbar interbody fusion; RCT = Randomized Controlled Trial; rhBMP-2 = recombinant human bone morphogenetic protein-2; ROM = range of motion; RTC = rectangular titanium cage; TLIF = transforaminal lumbar interbody fusion; VAS = visual analog scale; W-TLIF = TLIF through Wiltse approach.

Fusion rates of spinal surgery ranged from 65% to 100%, and the combined random-effect model fusion rate was 89.2% (95% CI, 87.4% to 91.1%; I^2^ = 86.9%, P < 0.001) (S1 Fig). Therefore, to explore the source of between-study heterogeneity, we stratified by some baseline study-level factors (all P < 0.001). Among these, we found fusion rates that were significantly different; for example, the combined fusion rate of studies with a female proportion below 50% was found to be 85.1% (95% CI, 79.4% to 90.8%; I^2^ = 91.7%, P < 0.001), which was significantly less than that in other studies. In addition, in the stratified analysis of surgical sites and surgical methods, we found that the fusion rate of cervical surgery was 92% (95% CI, 89.7% to 94.3%; I^2^ = 73.7%, P < 0.001), which was Higher than the fusion rate of lumbar surgery. And in lumbar fusion surgery, the fusion rate of lateral approach was significantly higher than that of other approaches (Rate, 95.3%; 95% CI, 90.8% to 99.7%; I^2^ = 70.2%, P = 0.035) (S5 Table).

### Risk factors and strength of evidence

**Fig 2:**
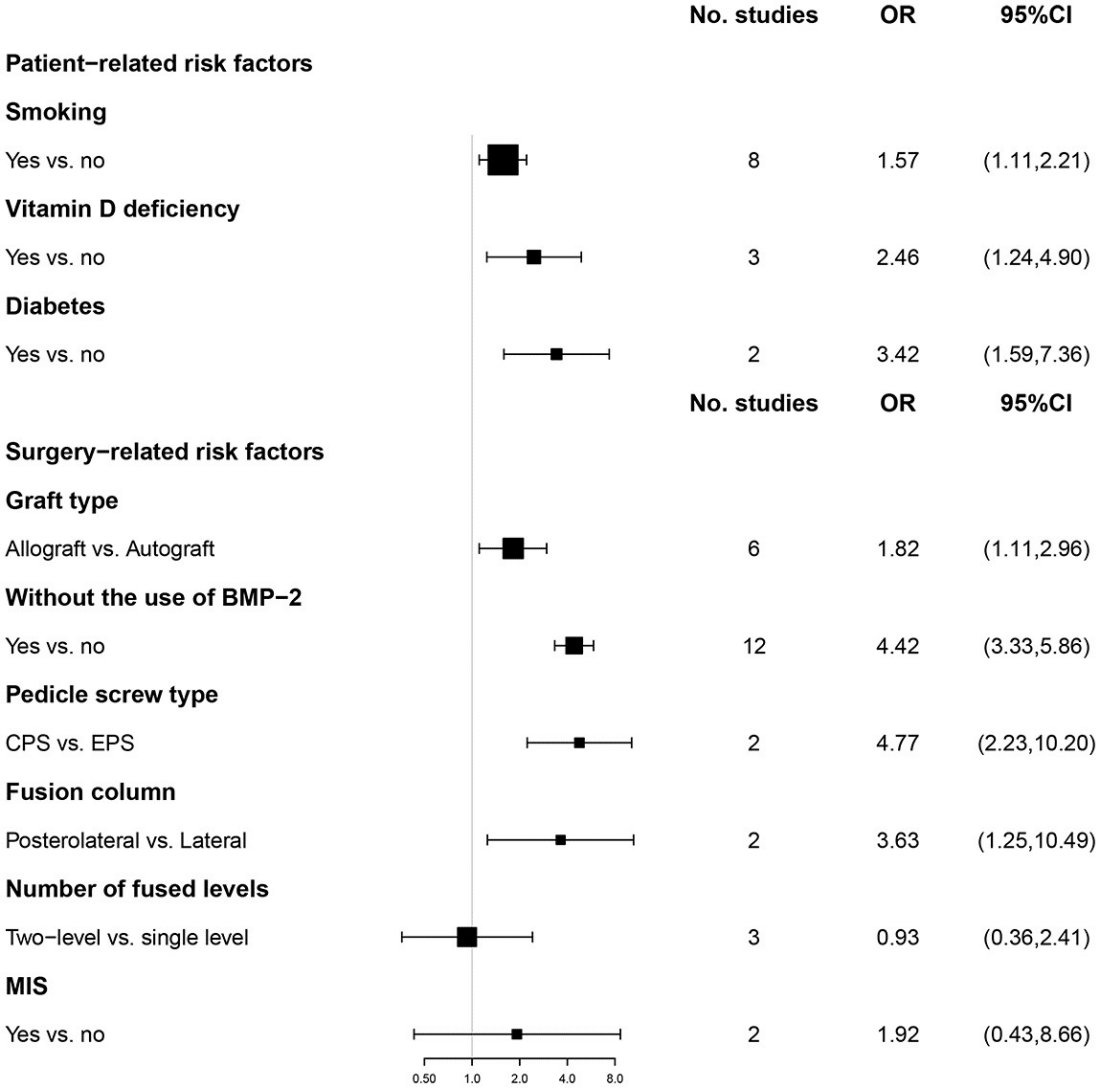
Meta-analyses of the association between patient-related risk factors and surgery-related risk factors.

Our study included the effects of patient-related and surgery-related risk factors (Fig 2) on spinal fusion. High-quality (Class I) evidence showed that patients with a smoking habit and without the use of BMP-2 were at higher risk for fusion failure. Medium-quality (Class II or III) evidence showed that fusion failure was significantly associated with vitamin D deficiency, diabetes, allograft, CPS fixation, and posterolateral fusion. Additionally, moderate-quality (Class II) evidence revealed nonsignificant correlations between MIS or the number of fused levels (two-level versus single-level) and fusion failure (Table 2 and S6 Table).

**Table 2:** Significant and non-significant risk factors associated with spinal fusion failure.

**Significant factors**	**No. of Studies**	**No. of Patients**	**OR (95% CI)**	**I** ^**2**^ **, %**	**P value**	**Egger’s test P value**
Smoking						
No			Ref.			
Yes	8	1672	1.57 (1.11 to 2.21)	0.0	0.010	0.702
Graft type						
Autograft			Ref.			
Allograft	6	460	1.82 (1.11 to 2.96)	25.7	0.018	0.024
Without the use of BMP-2						
No			Ref.			
Yes	12	3802	4.42 (3.33 to 5.86)	36.2	0.000	0.593
Vitamin D deficiency						
No			Ref.			
Yes	3	260	2.46 (1.24 to 4.90)	13.5	0.010	0.822
Pedicle screw type						
EPS			Ref.			
CPS	2	237	4.77 (2.23 to 10.20)	47.5	0.000	/
Diabetes						
No			Ref.			
Yes	2	233	3.42 (1.59 to 7.36)	0.0	0.002	/
Fusion column						
Lateral			Ref.			
Posterolateral	2	130	3.63 (1.25 to 10.49)	0.0	0.017	/
**Non-significant factors**	**No. of Studies**	**No. of Patients**	**OR (95% CI)**	**I** ^**2**^ **, %**	**P value**	**Egger’s test P value**
Number of fused levels						
Single			Ref.			
Two	3	282	0.93 (0.36 to 2.41)	19.6	0.887	0.025
MIS						
No			Ref.			
Yes	2	148	1.92 (0.43 to 8.66)	0.0	0.396	/

Abbreviations: BMP-2, bone morphogenetic protein-2; CI, confidence interval; CPS, conventional pedicle screws; EPS, expandable pedicle screws; MIS, minimally invasive surgery; OR, odds ratio; Ref, Reference group;

### Patient-related risk factors

#### Smoking

**Fig 3:**
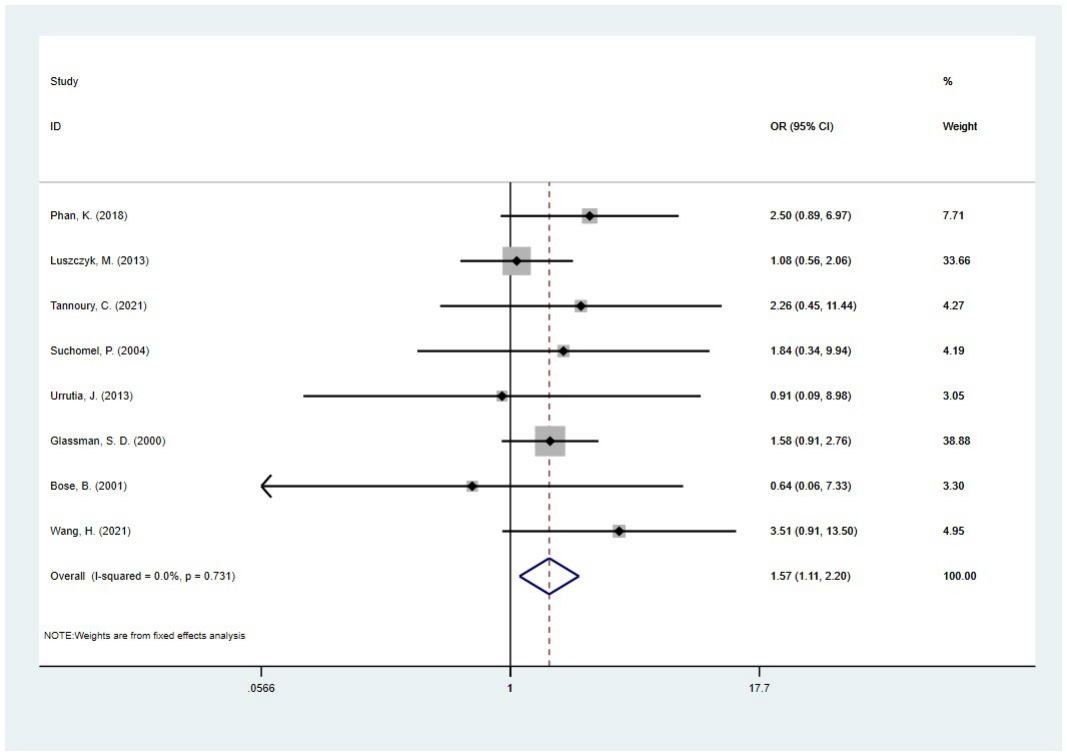
Odds ratio (OR) for association between smoking and fusion rate.

This meta-analysis showed that patients who smoked were at higher risk for fusion failure. The combined OR of 8 studies [32, 36, 38, 41, 44, 46, 48, 49] was 1.57 (95% CI, 1.11 to 2.21; I^2^ = 0.0%) (Fig 3). The trim-and-fill method was used to assess the robustness of the results, and we did not find potentially missing studies (S6 Table).

#### Diabetes

**Fig 4:**
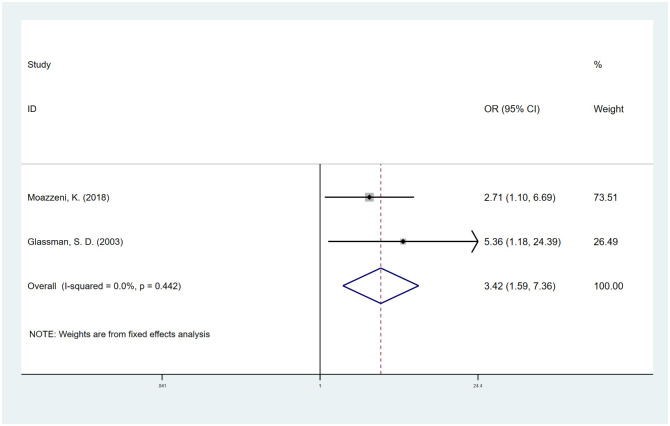
Odds ratio (OR) for association between diabetes and fusion rate.

We included 2 studies [35, 39] that evaluated the effect of diabetes on spinal fusion, and the combined OR was 3.42 (95% CI, 1.59 to 7.36; I^2^ = 0.0%) (Fig 4). We used the trim-and-fill method to adjust the publication bias and found that there was only one missing potential study in the funnel plots. The OR corrected for publication bias was 2.71 (95% CI, 1.36 to 5.41), which was largely consistent with our results (S6 Table).

#### Vitamin D deficiency

**Fig 5:**
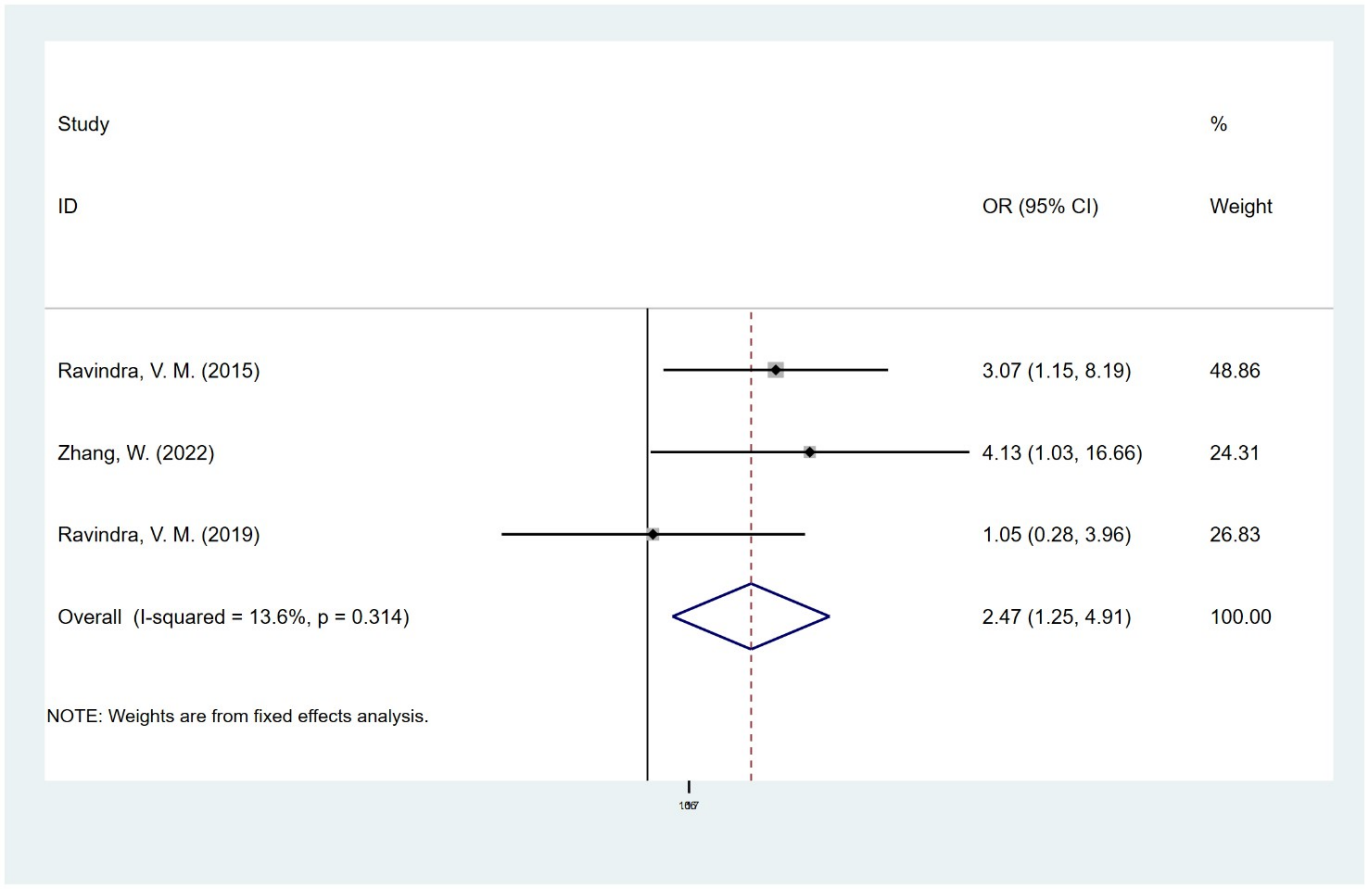
Odds ratio (OR) for association between vitamin D deficiency and fusion rate.

Three studies [20–22] reported the effect of vitamin D deficiency on spinal fusion. In our outcome, the risk of fusion failure in patients with vitamin D deficiency was significantly higher than that in patients without vitamin D deficiency (OR, 2.46; 95% CI, 1.24 to 4.90) (Fig 5), and we did not observe significant heterogeneity (I^2^ = 13.5%). We used the trim-and-fill method to adjust the publication bias and did not find missing potential studies (S6 Table).

### Surgery-related risk factors

#### BMP-2

**Fig 6:**
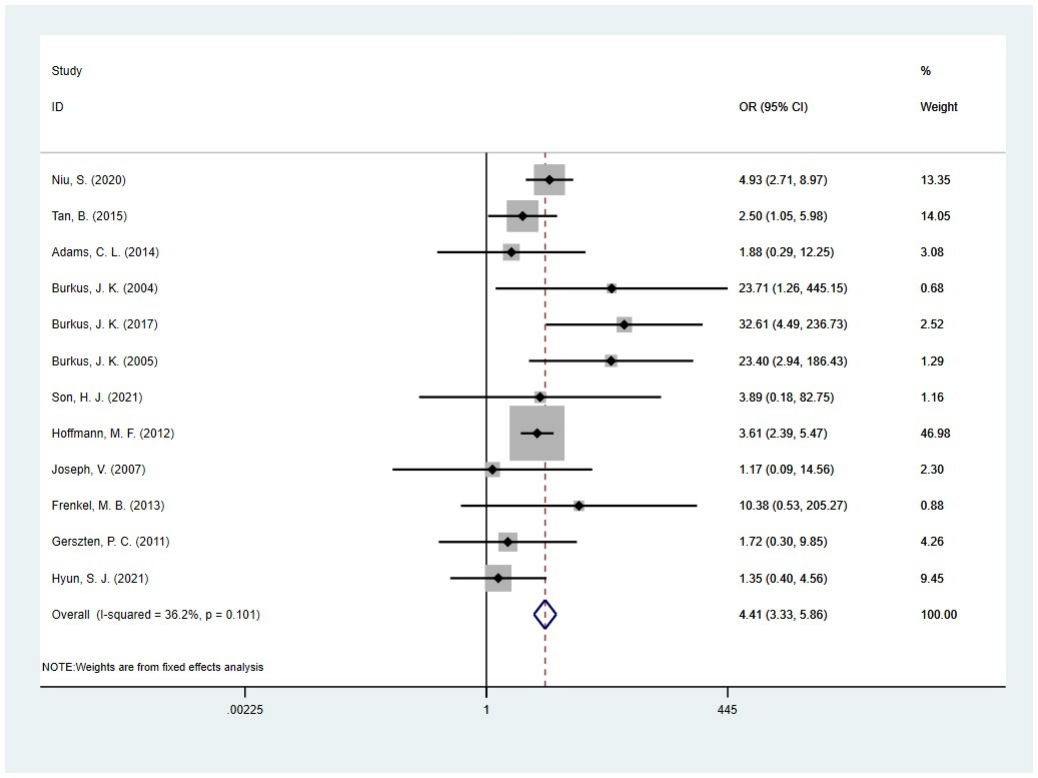
Odds ratio (OR) for association between without the use of BMP-2 (yes vs. no) and fusion rate.

We included 12 studies [8–12, 14–19, 45] that evaluated the effect of BMP-2 on spinal fusion (Fig 6). We found high-quality (Class I) evidence for a significant association between fusion failure and without the use of BMP-2 versus the use of BMP-2 (OR, 4.41; 95% CI, 3.33 to 5.86; I^2^ = 36.2%) (Table 2 and S6 Table). From our analysis, we found that the pooled OR was not significantly affected after removing any single study (lowest OR, 3.46; 95% CI, 2.49 to 4.79, highest OR, 3.88; 95% CI, 2.61 to 5.76) (S7 Table). The trim-and-fill method was used to adjust the publication bias, revealing a solitary absent potential study in the funnel plots. The OR corrected for publication bias was 3.58 (95% CI, 2.70 to 4.75), which was basically consistent with our results (S6 Table).

#### Graft type

**Fig 7:**
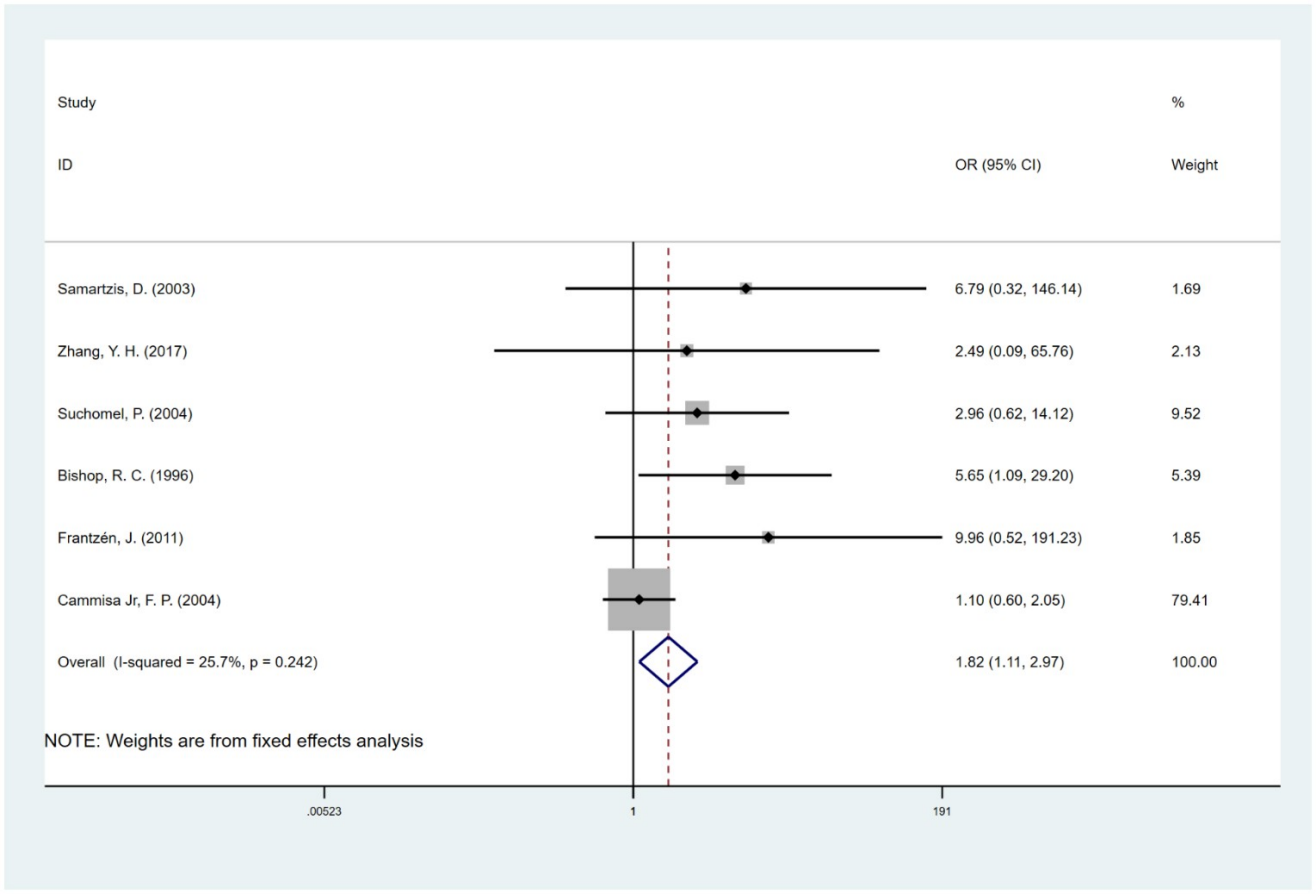
Odds ratio (OR) for association between graft type (allograft vs. autograft) and fusion rate.

The combined results of 6 studies [31, 33, 34, 42, 44, 53] showed that compared with autografts, the risk of fusion failure of allografts was higher (OR, 1.82; 95% CI, 1.11 to 2.97; I^2^ = 25.7%) (Fig 7). However, after adjusting for publication bias by the trim-and-fill method, the pooled OR was 1.28 (95% CI, 0.79 to 2.07) (S6 Table), which was different from our results.

#### Pedicle screw type

**Fig 8:**
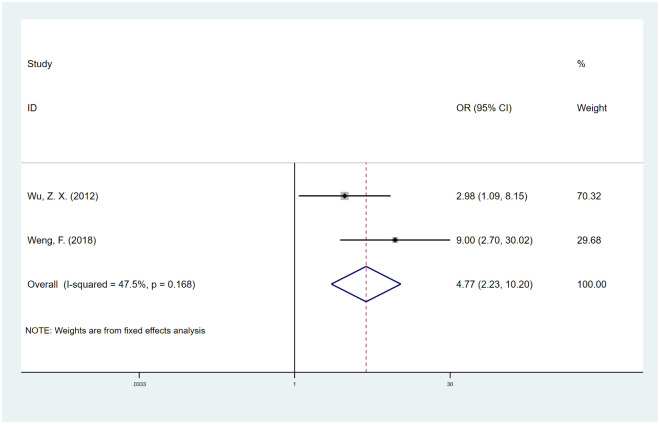
Odds ratio (OR) for association between pedicle screw type (CPS vs. EPS) and fusion rate.

Data from 2 studies [50, 52] suggested that there was a higher risk of fusion failure with CPS fixation than with expandable pedicle screw (EPS) fixation (OR, 4.77; 95% CI, 2.23 to 10.20; I^2^ = 47.5%) (Fig 8). We used the trim-and-fill method to adjust the publication bias, and the OR corrected for publication bias was 2.98 (95% CI, 1.56 to 5.81), which was essentially in line with our results (S6 Table).

#### Fusion column

**Fig 9:**
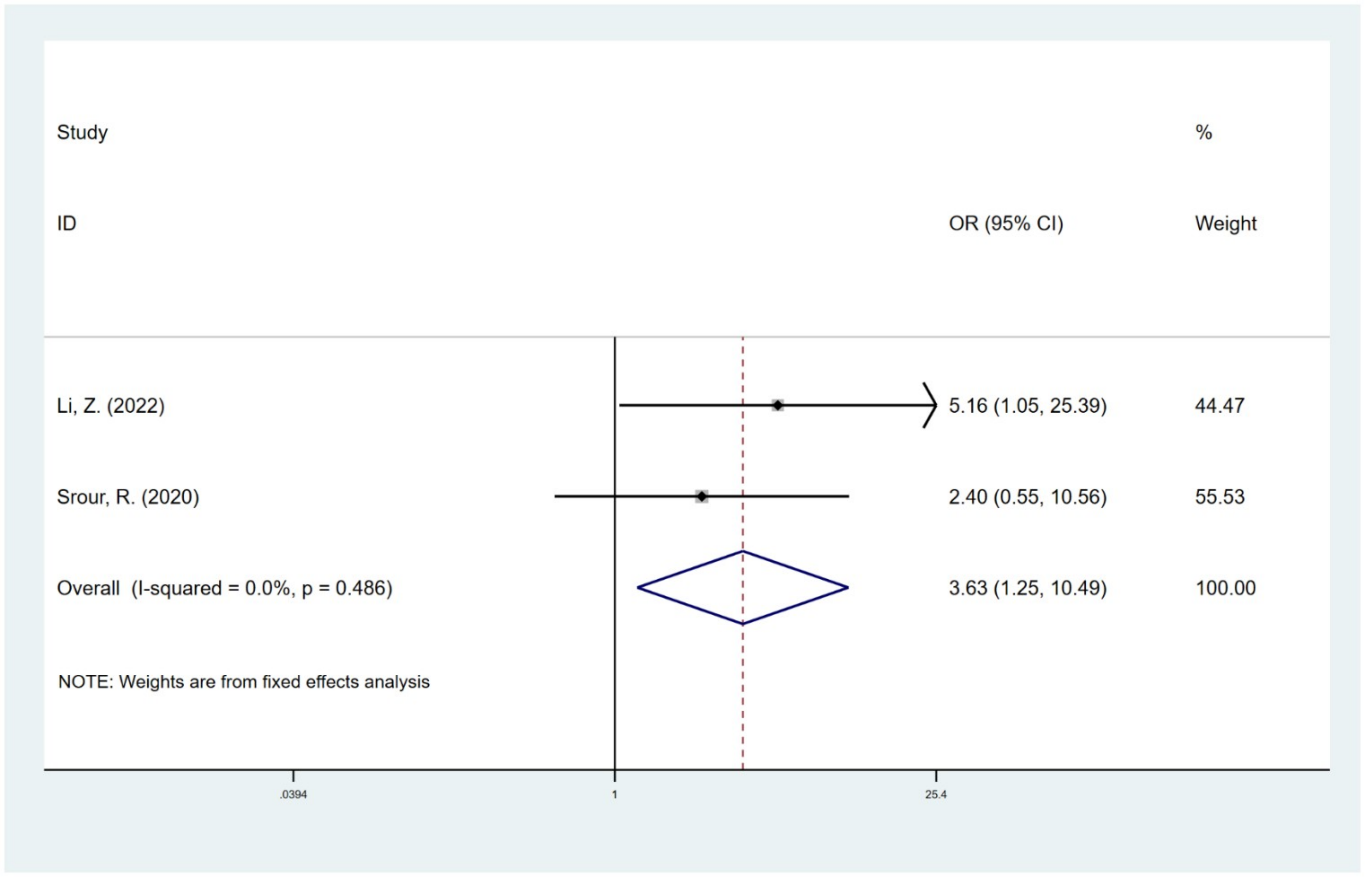
Odds ratio (OR) for association between fusion column (posterolateral vs. lateral) and fusion rate.

The combined results of 2 studies [37, 43] suggested that compared with posterolateral fusion, lateral fusion may increase the risk of fusion failure (OR, 3.63; 95% CI, 1.25 to 10.49; I^2^ = 0.0%) (Fig 9). The trim-and-fill method was employed to evaluate the robustness of the outcome, and only one potential study was identified. The OR corrected for publication bias was 2.40 (95% CI, 0.98 to 5.88), which diverges from the outcomes obtained in our study (S6 Table).

#### Number of fused levels

**Fig 10:**
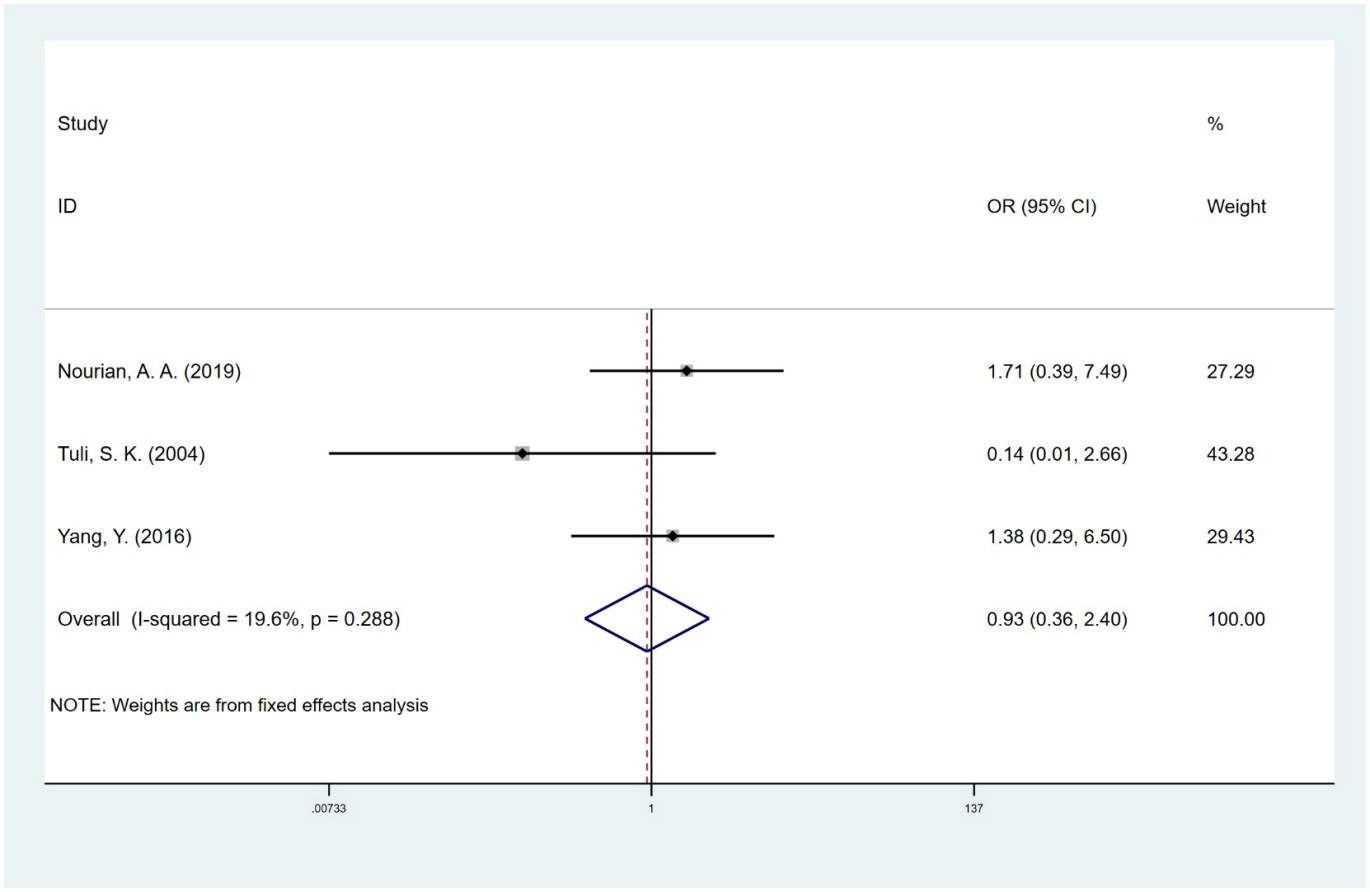
Odds ratio (OR) for association between number of fused levels (two vs. single) and fusion rate.

We included 3 studies [40, 47, 54] that evaluated the effect of the number of fused levels on spinal fusion. Their results showed that there was no significant association between fusion failure and two-level fusions versus single-level fusion (OR, 0.93; 95% CI, 0.36 to 2.41; I^2^ = 19.6%) (Fig 10). We used the trim-and-fill method to adjust the publication bias and did not find missing potential studies (S6 Table).

#### MIS

**Fig 11:**
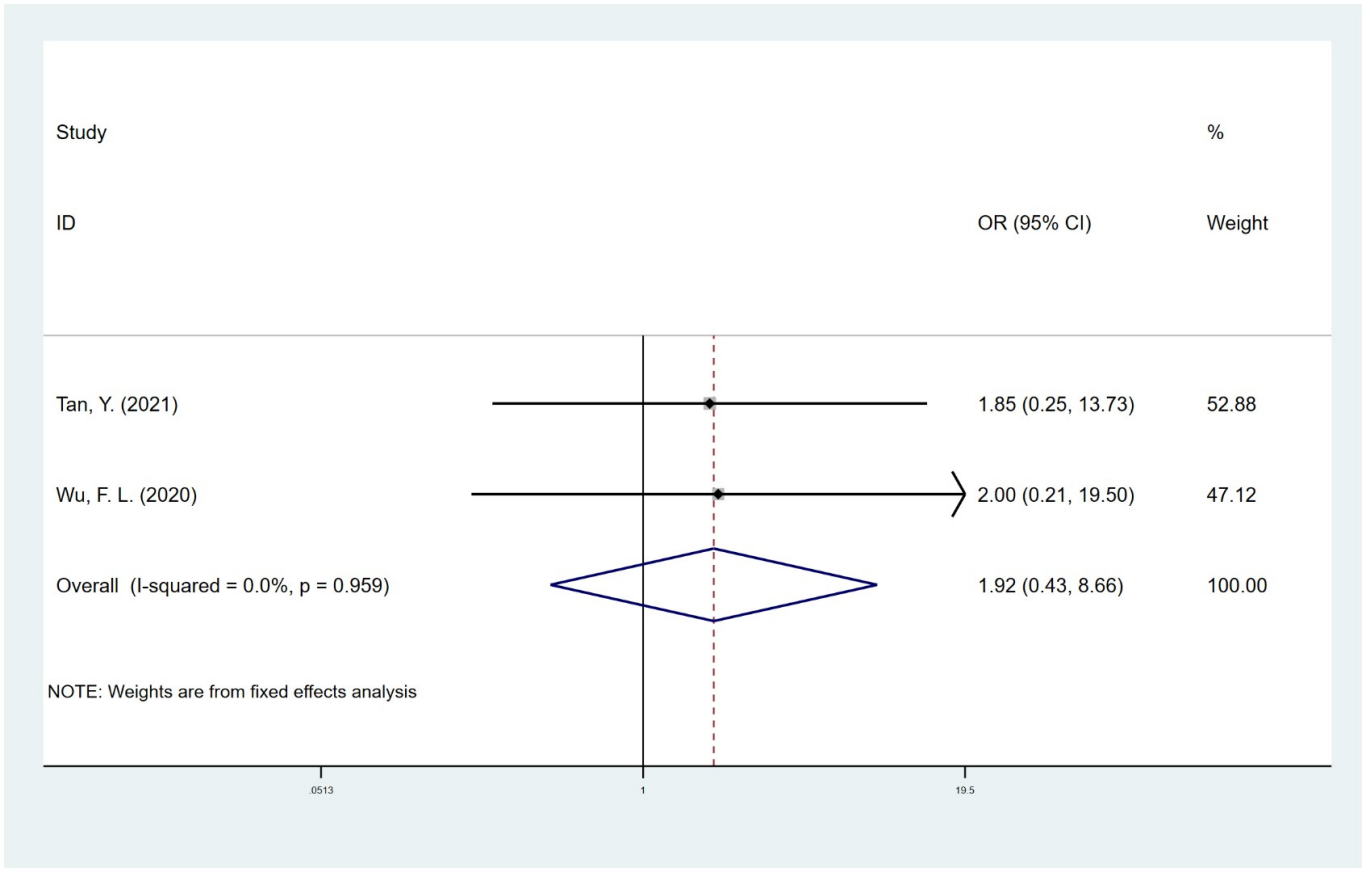
Odds ratio (OR) for association between MIS and fusion rate.

Two studies [13, 51] reported the effect of MIS on spinal fusion; however, no significant correlation was observed between the two (OR, 1.92; 95% CI, 0.43 to 8.66; I^2^ = 0.0%) (Fig 11). The trim-and-fill method was employed to address publication bias, resulting in the identification of only one potential study that was missing. The OR corrected for publication bias by the trim-and-fill method was 1.85 (95% CI, 0.53 to 6.46), which was basically consistent with our results (S6 Table).

### Sensitivity analyses and publication bias

We used leave-one-out sensitivity analysis to evaluate the stability of the results for factors reported by more than two articles. The results showed that the pooled ORs all remained similar across these analyses for both patient-related and surgery-related risk factors (S7 Table). Furthermore, funnel plots were employed to evaluate the potential presence of publication bias associated with these risk factors (S2–S10 Figs) and we didn’t find any obvious bias.

## Discussion

### Principal findings

This meta-analysis was designed to identify risk factors affecting spinal fusion and to grade the level of evidence, and a total of 39 studies were included. We identified 3 patient-related risk factors, including smoking, diabetes, and vitamin D deficiency, and four surgery-related risk factors, including allografting, without the use of BMP-2, CPS fixation, and posterolateral fusion.

The meta-analysis revealed that MIS or the number of fused levels (two-level versus single-level) was not significantly linked to fusion failure. However, we cannot dismiss these factors as potential risk factors, as some studies have demonstrated a significant association with a low fusion rate [13, 47, 51]. Hence, it is advisable to carry out additional clinical studies on these variables.

### Potential mechanisms

The underlying mechanisms of various factors affecting spinal fusion have not been clarified until now. Smoking has been shown to impair skeletal healing and metabolism. Experiments have shown that nicotine can reduce neovascularization and inhibit osteoblast differentiation, resulting in bone healing defects [55–57]. In our study, diabetes was one of the significant risk factors for spinal fusion (OR, 3.42; 95% CI, 1.59 to 7.36). Diabetes is a multiorgan disease, and its complications may lead to multisystem organ failure, resulting in poor surgical outcomes [58]. Additionally, studies have confirmed that vitamin D levels are significantly associated with bone mineral density; thus, vitamin D deficiency may result in bone nonunion or prolonged fusion time [20, 21]. This is consistent with our findings (OR, 2.47; 95% CI, 1.25 to 4.91).

BMP-2 is an osteoinductive growth factor that belongs to the transforming growth factor-β (TGF-β) superfamily; it can stimulate pluripotent cells to form bone, and it is the only bone inducer with level I clinical evidence [8, 11, 59]. BMP-2 was introduced in the medical scenario to promote bone healing with the proposal of less morbidity compared to the usual methods of bone graft harvest [59]. Niu et al. reported that patients who used BMP-2 had better fusion rates than patients who were without the use of BMP-2 [8–13]. Moreover, we found that even if there are some complications, autografts remain the gold standard for interbody grafts in spinal fusion [44]. It has been demonstrated that autografts contain viable osteoblasts and osteogenic precursor cells that can contribute to the formation of new bone, thus improving the fusion rate [60]. However, allografts are considered to have high osteoconductive properties [61], weak osteoinductive potential, and non-osteogenic properties [62, 63]. Therefore, autografts provide better conditions for bone fusion and a higher fusion rate than allografts, which was also fully reflected in our research.

Additionally, Wu et al. and Weng et al. showed that compared with EPS fixation, CPS fixation has lower stability than internal fixation [50, 52]. EPS can fix the vertical axial section through the front expansive effect [64], thus forming triangular support [65] and significantly enhancing screw bonding [66]; in parallel, the surrounding bone trabecula is appropriately compressed, which consequently enhances both bone density and the stability of internal fixation [67]. Hence, the fusion rate of EPS fixation surpasses that of CPS fixation.

### Implications

Our study comprehensively shows the risk factors that may affect spinal fusion, and the identification of these factors can help clinicians to conduct a more comprehensive preoperative risk assessment of patients and early intervention, while developing appropriate surgical strategies for patients to reduce the risk of fusion failure. Therefore, conducting extensive prospective cohort studies is essential to validate these findings.

### Strengths

The advantages of our study are as follows. First, to the best of our knowledge, this is the first meta-analysis to assess all the risk factors that may affect spinal fusion. It provides the latest and most comprehensive evidence of risk factors affecting spinal fusion, including smoking, diabetes, vitamin D deficiency, allograft, without the use of BMP-2, CPS fixation, and posterolateral fusion. Second, to maximize the retrieval of original literature meeting the inclusion criteria and mitigate publication bias in the combined results, we developed an extensive database search strategy encompassing PubMed, Cochrane Library, and Embase, without imposing any date restrictions. Third, we also calculated the pooled fusion rate of spinal surgery by a random effect model and analyzed some factors at the baseline study level. Fourth, we assessed the strength of correlation for each risk factor (from Class I to Class IV) by considering factors such as sample size, Egger’s P value, and heterogeneity. Lastly, we employed a range of rigorous methods to assess the robustness of our findings, such as sensitivity analysis and the trim-and-fill method.

### Limitations

The study has the following limitations despite its abovementioned strengths. First, our data sources were based on cohort studies, and the related risk factors that lead to an increase in the risk of fusion failure are diverse and complex; they were to some extent subject to selection bias. Second, few studies were involved in the analysis of some of the risk factors, making it difficult to accurately assess their relationship with spinal fusion, highlighting the need for future high-quality large cohort studies. Finally, given the absence of established gold standards or guidelines for quantitatively evaluating the strength of risk factor meta-analysis evidence, we employed three criteria (Egger’s P value, sample size, and I^2^ statistics) to classify the level of evidence intensity in accordance with existing literature.

## Conclusions

In conclusion, the current meta-analysis showed conspicuous risk factors affecting spinal fusion, including three patient-related risk factors (smoking, vitamin D deficiency, diabetes) and four surgery-related risk factors (without the use of BMP-2, allograft, CPS fixation, and posterolateral fusion). These findings may help clinicians strengthen awareness for early intervention in patients at high risk of developing fusion failure.

## Supporting information

S1 Checklist PRISMA checklist. (DOCX)

S2 Checklist PRISMA 2020 checklist. (DOCX)

S1 Table General search strategies for PubMed, Embase and Cochrane Library. (DOCX)

S2 Table Methodological quality score of the included studies based on the Newcastle—Ottawa Scale (NOS) tool. (DOCX)

S3 Table Grading evidence based on Egger’s P value, sample size and heterogeneity. (DOCX)

S4 Table List of included and excluded studies. (DOCX)

S5 Table Fusion rates by study-level factors. (DOCX)

S6 Table Sensitivity analysis for significant and non-significant factors and class of evidence. (DOCX)

S7 Table Sensitivity analysis for fusion rate associated with patient-related factors and surgery-related factors. (DOCX)

S1 Fig Forest plot for pooled fusion rate. (TIF)

S2 Fig Funnel plots for meta-analysis of association between smoking and fusion rate. (TIF)

S3 Fig Funnel plots for meta-analysis of association between graft type (allograft vs. autograft) and fusion rate. (TIF)

S4 Fig Funnel plots for meta-analysis of association between number of fused levels (two vs. single) and fusion rate. (TIF)

S5 Fig Funnel plots for meta-analysis of association between without the use of BMP-2 (yes vs. no) and fusion rate. (TIF)

S6 Fig Funnel plots for meta-analysis of association between vitamin D deficiency and fusion rate. (TIF)

S7 Fig Funnel plots for meta-analysis of association between pedicle screw type (CPS vs. EPS) and fusion rate. (TIF)

S8 Fig Funnel plots for meta-analysis of association between diabetes and fusion rate. (TIF)

S9 Fig Funnel plots for meta-analysis of association between MIS and fusion rate. (TIF)

S10 Fig Funnel plots for meta-analysis of association between fusion column (posterolateral vs. lateral) and fusion rate. (TIF)
