# Supplementary material for: Risk factors affecting spinal fusion: A meta-analysis of 39 cohort studies
Source: PLoS One. 2024 Jun 7;19(6):e0304473. doi: 10.1371/journal.pone.0304473 (PMC11161075; doi:10.1371/journal.pone.0304473)
Supplement: S1 Table — (DOCX) [file pone.0304473.s003.docx]

**S1 Table.** General Search Strategies for PubMed, Embase and Cochrane Library.

**Text S1 Search strategy**

**Database: PubMed from inception to Present> (Search date: January 6, 2023)**

**Search Strategy:**

--------------------------------------------------------------------------------

***Spine surgery terms (P):***

#1 "Spinal fusion"[Mesh]

#2 (Spine fusion or Spinal Fusion or spinal fusion surgery or spinal arthrodes or cervical fusion or lumbar fusion or lumbosacral fusion or interbody fusion or posterolateral fusion or anterior fusion or posterior fusion or lateral fusion or transforaminal interbody fusion or cervical arthrodes or lumbar arthrodes or lumbosacral arthrodes or interbody arthrodes or posterolateral arthrodes or disc fusion) [Title/Abstract]

#3 #1 or #2

***Factors term (I):***

#4 "Obesity"[Mesh]

#5 "Electric Stimulation Therapy"[Mesh]

#6 "Smoke"[Mesh]

#7 "Osteoporosis"[Mesh]

#8 "Vitamin D"[Mesh]

#9 (Old age or women or obesity or BMI or weight or electrical stimulation or electrical current stimulation or direct current stimulation or electromagnetic field stimulation or pulsed electromagnetic field stimulation or pulsing electromagnetic field or capacitive couple or capacitive coupling or capacitive coupled or smoke* or smoking or tobacco or osteoporosis or bisphosphonates or etidronate or clodronate or ibandronate or alendronate or risedronate or pamidronate or zoledronate or parathyroid hormone or teriparatide or diabetes or diabetes mellitus or DM or vitamin D or ergocalciferols or vitamin D deficiency or cholecalciferol or calcifediol or 25-hydroxyvitamin D or hypertension or Bone Morphogenetic Protein 2 or BMP-2 or worker compensation status or fusion cage height or cage height or polymethylmethacrylate or biocompatible osteoconductive polymer or NSAID or NSAIDs or nonsteroidal anti-inflammatory drugs or History of Cardiac Disease) [Title/Abstract]

#10 #4-9/or

***Fusion terms (O):***

#11 (Fusion rate or fusion rates or fixation rate or fixation rates or arthrodesis rate) [Title/Abstract]

***Final search results: Combining Fusion and Factors and Spine surgery terms:***

#12 #3 and #10 and #11 (595)

**Text S2 Search strategy**

**Database: EMBASE from inception to Present> (Search date: January 6, 2023)**

**Search Strategy:**

--------------------------------------------------------------------------------

***Spine surgery terms（P）:***

#1 ‘Spine fusion’/exp

#2 (Spine fusion or Spinal Fusion or spinal fusion surgery or spinal arthrodes or cervical fusion or lumbar fusion or lumbosacral fusion or interbody fusion or posterolateral fusion or anterior fusion or posterior fusion or lateral fusion or transforaminal interbody fusion or cervical arthrodes or lumbar arthrodes or lumbosacral arthrodes or interbody arthrodes or posterolateral arthrodes or disc fusion):ab,ti

#3 #1 or #2

***Factors term (I):***

#4 ‘Obesity’/exp

#5 ‘Electrotherapy’/exp

#6 ‘Smoke’/exp

#7 ‘Osteoporosis’/exp

#8 ‘Vitamin D’/exp

#9 (Old age or women or obesity or BMI or weight or electrical stimulation or electrical current stimulation or direct current stimulation or electromagnetic field stimulation or pulsed electromagnetic field stimulation or pulsing electromagnetic field or capacitive couple or capacitive coupling or capacitive coupled or smoke* or smoking or tobacco or osteoporosis or bisphosphonates or etidronate or clodronate or ibandronate or alendronate or risedronate or pamidronate or zoledronate or parathyroid hormone or teriparatide or diabetes or diabetes mellitus or DM or vitamin D or ergocalciferols or vitamin D deficiency or cholecalciferol or calcifediol or 25-hydroxyvitamin D or hypertension or Bone Morphogenetic Protein 2 or BMP-2 or worker compensation status or fusion cage height or cage height or polymethylmethacrylate or biocompatible osteoconductive polymer or NSAID or NSAIDs or nonsteroidal anti-inflammatory drugs or History of Cardiac Disease):ab,ti

#10 #4-9/or

***Fusion terms (O):***

#11 (Fusion rate or fusion rates or fixation rate or fixation rates or arthrodesis rate):ab,ti

***Final search results: Combining Fusion and Factors and Spine surgery terms:***

#12 #3 and #10 and #11 (139)

**Text S3 Search strategy**

**Database: Cochrane Library from inception to Present> (Search date: January 6, 2023)**

**Search Strategy:**

--------------------------------------------------------------------------------

***Spine surgery terms（P）:***

#1 MeSH descriptor: [Spinal fusion] explode all trees

#2 (Spine fusion or Spinal Fusion or spinal fusion surgery or spinal arthrodes or cervical fusion or lumbar fusion or lumbosacral fusion or interbody fusion or posterolateral fusion or anterior fusion or posterior fusion or lateral fusion or transforaminal interbody fusion or cervical arthrodes or lumbar arthrodes or lumbosacral arthrodes or interbody arthrodes or posterolateral arthrodes or disc fusion):ti,ab,kw (Word variations have been searched)

#3 #1 or #2

***Factors term (I):***

#4 MeSH descriptor: [Obesity] explode all trees

#5 MeSH descriptor: [Electric Stimulation Therapy] explode all trees

#6 MeSH descriptor: [Tobacco use] explode all trees

#7 MeSH descriptor: [Osteoporosis] explode all trees

#8 MeSH descriptor: [Vitamin D] explode all trees

#9 (Old age or women or obesity or BMI or weight or electrical stimulation or electrical current stimulation or direct current stimulation or electromagnetic field stimulation or pulsed electromagnetic field stimulation or pulsing electromagnetic field or capacitive couple or capacitive coupling or capacitive coupled or smoke or smoking or tobacco use or osteoporosis or bisphosphonates or etidronate or clodronate or ibandronate or alendronate or risedronate or pamidronate or zoledronate or parathyroid hormone or teriparatide or diabetes or diabetes mellitus or DM or vitamin D or ergocalciferols or vitamin D deficiency or cholecalciferol or calcifediol or 25 hydroxyvitamin D or hypertension or Bone Morphogenetic Protein 2 or BMP 2 or worker compensation status or fusion cage height or cage height or polymethylmethacrylate or biocompatible osteoconductive polymer or NSAID or NSAIDs or nonsteroidal anti-inflammatory drugs or History of Cardiac Disease):ti,ab,kw (Word variations have been searched)

#10 #4-9/or

***Fusion terms (O):***

#11 (Fusion rate or fusion rates or fixation rate or fixation rates or arthrodesis rate):ti,ab,kw (Word variations have been searched)

***Final search results: Combining Fusion and Factors and Spine surgery terms:***

#12 #3 and #10 and #11 (523)
