# Supplementary material for: Risk factors affecting spinal fusion: A meta-analysis of 39 cohort studies
Source: PLoS One. 2024 Jun 7;19(6):e0304473. doi: 10.1371/journal.pone.0304473 (PMC11161075; doi:10.1371/journal.pone.0304473)
Supplement: S2 Table — (DOCX) [file pone.0304473.s004.docx]

**S2 Table.** Methodological quality score of the included studies based on the Newcastle–Ottawa Scale (NOS) tool.

| **Author** | **year** | **Study Design** | **Selection** | | | | **Comparability** | **Exposure/Outcome** | | | **Total Score** | **Risk  of Bias** |
| --- | --- | --- | --- | --- | --- | --- | --- | --- | --- | --- | --- | --- |
|  |  |  | **Representative­ness of cohort *** | Selection of control cohort * | Ascertainment of exposure * | Outcome not present at start * | Comparability of cohorts ** | Assessment of outcome * | Length of follow-up * | Adequacy of follow-up * | **Total score 9*** |  |
| Glassman, S. D. | 2000 | Retrospective study | * | * | * | * | ** | * | * | * | 9 | Low |
| Bose, B. | 2001 | Retrospective study | * | * | * | * | ** | * | * |  | 8 | Low |
| Samartzis, D. | 2003 | Retrospective study | * | * | * | * | * | * | * | * | 8 | Low |
| Glassman, S. D. | 2003 | Retrospective study | * | * | * | * | ** | * | * | * | 9 | Low |
| Gerszten, P. C. | 2011 | Retrospective study | * | * | * | * | ** | * | * |  | 8 | Low |
| Hoffmann, M. F. | 2012 | Retrospective study | * | * | * | * | * | * | * | * | 8 | Low |
| Luszczyk, M. | 2013 | Retrospective study | * | * | * | * | * | * | * | * | 8 | Low |
| Urrutia, J. | 2013 | Retrospective study | * | * | * | * | ** | * | * | * | 9 | Low |
| Frenkel, M. B. | 2013 | Retrospective study | * | * | * | * | ** | * | * | * | 9 | Low |
| Adams, C. L. | 2014 | Retrospective cohort study | * | * | * | * | ** | * | * |  | 8 | Low |
| Tan, B. | 2015 | Retrospective study | * | * | * | * | ** | * | * |  | 8 | Low |
| Yang, Y. | 2016 | Retrospective study | * | * | * | * | ** | * | * | * | 9 | Low |
| Zhang, Y. H. | 2017 | Retrospective study | * | * | * | * | ** | * | * | * | 9 | Low |
| Phan, K. | 2018 | Retrospective study | * | * | * | * | ** | * | * | * | 9 | Low |
| Weng, F. | 2018 | Retrospective study | * | * | * | * | ** | * |  |  | 7 | High |
| Nourian, A. A. | 2019 | Retrospective study | * | * | * | * | * | * | * | * | 8 | Low |
| Niu, S. | 2020 | Retrospective study | * | * | * | * | ** | * |  | * | 8 | Low |
| Wu, F. L. | 2020 | Retrospective study | * | * | * | * | * | * | * | * | 8 | Low |
| Son, H. J. | 2021 | Retrospective study | * | * | * | * | * | * | * |  | 7 | High |
| Tan, Y. | 2021 | Retrospective study | * | * | * | * | * | * | * |  | 7 | High |
| Tannoury, C. | 2021 | Retrospective study | * | * | * | * | * | * | * | * | 8 | Low |
| Wang, H. | 2021 | Retrospective study | * | * | * | * | * | * | * |  | 8 | Low |
| Li, Z. | 2022 | Retrospective study | * | * | * | * | ** | * | * | * | 9 | Low |
| Bishop, R. C. | 1996 | Prospective study | * | * | * | * | * | * | * |  | 7 | High |
| Tuli, S. K. | 2004 | Prospective study | * | * | * | * | ** | * |  | * | 8 | Low |
| Suchomel, P. | 2004 | Prospective study | * | * | * | * | * | * | * | * | 8 | Low |
| Burkus, J. K. | 2004 | Prospective study | * | * | * | * | * | * | * |  | 7 | High |
| Cammisa Jr, F. P. | 2004 | Prospective study | * | * | * | * | ** | * | * | * | 9 | Low |
| Burkus, J. K. | 2005 | Prospective study | * | * | * | * | * | * | * | * | 8 | Low |
| Joseph, V. | 2007 | Prospective study | * | * | * | * | * | * | * |  | 7 | High |
| Frantzén, J. | 2011 | Prospective study | * | * | * | * | * | * | * | * | 8 | Low |
| Wu, Z. X. | 2012 | Prospective study | * | * | * | * | * | * | * | * | 8 | Low |
| Ravindra, V. M. | 2015 | Prospective study | * | * | * | * | ** | * | * | * | 9 | Low |
| Burkus, J. K. | 2017 | Prospective study | * | * | * | * | * | * | * | * | 8 | Low |
| Moazzeni, K. | 2018 | Prospective study | * | * | * | * | * | * | * |  | 7 | High |
| Ravindra, V. M. | 2019 | Prospective study | * | * | * | * | ** | * | * | * | 9 | Low |
| Srour, R. | 2020 | Prospective study | * | * | * | * | * | * | * |  | 7 | High |
| Hyun, S. J. | 2021 | Prospective study | * | * | * | * | * | * | * |  | 7 | High |
| Zhang, W. | 2022 | Prospective study | * | * | * | * | ** | * |  | * | 8 | Low |
