# Supplementary material for: Risk factors affecting spinal fusion: A meta-analysis of 39 cohort studies
Source: PLoS One. 2024 Jun 7;19(6):e0304473. doi: 10.1371/journal.pone.0304473 (PMC11161075; doi:10.1371/journal.pone.0304473)
Supplement: S3 Table — (DOCX) [file pone.0304473.s005.docx]

**S3 Table.** Grading Evidence Based on Egger’s *P* value, Sample Size and Heterogeneity.

| **Evidence** | **Definition** |
| --- | --- |
| Class 1 (High-quality) evidence | Defined as three conditions of Egger’s P value of the random effects model >0.1, a total population >500, and lower between-study heterogeneity I^2^<50% are met simultaneously. |
| Class II (Moderate-quality) evidence | Defined as two of the three conditions of Egger’s P value of the random effects model >0.1, a pooled population >500, and higher between-study heterogeneity I^2^ <50% are met. |
| Class III (Moderate-quality) evidence | Defined as one of the three conditions of Egger’s P value of the random effects model >0.1, a pooled population >500, and lower between-study heterogeneity I^2^<50% are met. |
| Class IV (Low-quality) evidence | Defined as none of the three conditions of Egger’s P value of the random effects model >0.1, a pooled population >500, and higher between-study heterogeneity I^2^<50% are met. |
