# Supplementary material for: Risk factors affecting spinal fusion: A meta-analysis of 39 cohort studies
Source: PLoS One. 2024 Jun 7;19(6):e0304473. doi: 10.1371/journal.pone.0304473 (PMC11161075; doi:10.1371/journal.pone.0304473)
Supplement: S4 Table — (DOCX) [file pone.0304473.s006.docx]

**S4 Table.** List of Included and Excluded Studies.

| Included and excluded reasons | No. studies | References. |
| --- | --- | --- |
| Included studies | 39 | [1-39] |
| Review or meta-analysis | 13 | [40-52] |
| Inadequate outcome data | 35 | [53-87] |
| Randomized controlled trial | 18 | [88-105] |
| The literature is not in English | 8 | [106-113] |
| Animal experiment | 5 | [114-118] |

REFERENCES

1. Adams CL, Ogden K, Robertson IK, Broadhurst S, Edis D. Effectiveness and safety of recombinant human bone morphogenetic protein-2 versus local bone graft in primary lumbar interbody fusions. Spine (Phila Pa 1976). 2014;39(2):164-71. Epub 2013/10/25. doi: 10.1097/brs.0000000000000089. PubMed PMID: 24153173.

2. Bishop RC, Moore KA, Hadley MN. Anterior cervical interbody fusion using autogeneic and allogeneic bone graft substrate: a prospective comparative analysis. J Neurosurg. 1996;85(2):206-10. Epub 1996/08/01. doi: 10.3171/jns.1996.85.2.0206. PubMed PMID: 8755747.

3. Bose B. Anterior cervical instrumentation enhances fusion rates in multilevel reconstruction in smokers. J Spinal Disord. 2001;14(1):3-9. Epub 2001/03/10. doi: 10.1097/00002517-200102000-00002. PubMed PMID: 11242268.

4. Burkus JK. Bone morphogenetic proteins in anterior lumbar interbody fusion: old techniques and new technologies. Invited submission from the Joint Section Meeting on Disorders of the Spine and Peripheral Nerves, March 2004. Journal of neurosurgery Spine. 2004;1(3):254‐60. PubMed PMID: CN-01713120.

5. Burkus JK, Dryer RF, Arnold PM, Foley KT. Clinical and Radiographic Outcomes in Patients Undergoing Single-level Anterior Cervical Arthrodesis: A Prospective Trial Comparing Allograft to a Reduced Dose of rhBMP-2. Clin Spine Surg. 2017;30(9):E1321-e32. Epub 2016/06/29. doi: 10.1097/bsd.0000000000000409. PubMed PMID: 27352370.

6. Burkus JK, Sandhu HS, Gornet MF, Longley MC. Use of rhBMP-2 in combination with structural cortical allografts: clinical and radiographic outcomes in anterior lumbar spinal surgery. Journal of bone and joint surgery American volume. 2005;87(6):1205‐12. doi: 10.2106/JBJS.D.02532. PubMed PMID: CN-00514382.

7. Cammisa FP, Jr., Lowery G, Garfin SR, Geisler FH, Klara PM, McGuire RA, et al. Two-year fusion rate equivalency between Grafton DBM gel and autograft in posterolateral spine fusion: a prospective controlled trial employing a side-by-side comparison in the same patient. Spine (Phila Pa 1976). 2004;29(6):660-6. Epub 2004/03/12. doi: 10.1097/01.brs.0000116588.17129.b9. PubMed PMID: 15014276.

8. Frantzén J, Rantakokko J, Aro HT, Heinänen J, Kajander S, Gullichsen E, et al. Instrumented spondylodesis in degenerative spondylolisthesis with bioactive glass and autologous bone: a prospective 11-year follow-up. J Spinal Disord Tech. 2011;24(7):455-61. Epub 2011/09/13. doi: 10.1097/BSD.0b013e31822a20c6. PubMed PMID: 21909036.

9. Frenkel MB, Cahill KS, Javahary RJ, Zacur G, Green BA, Levi AD. Fusion rates in multilevel, instrumented anterior cervical fusion for degenerative disease with and without the use of bone morphogenetic protein. J Neurosurg Spine. 2013;18(3):269-73. Epub 2013/01/29. doi: 10.3171/2012.12.Spine12607. PubMed PMID: 23350532.

10. Gerszten PC, Tobler WD, Nasca RJ. Retrospective analysis of L5-S1 axial lumbar interbody fusion (AxiaLIF): a comparison with and without the use of recombinant human bone morphogenetic protein-2. Spine J. 2011;11(11):1027-32. Epub 2011/11/30. doi: 10.1016/j.spinee.2011.10.006. PubMed PMID: 22122835.

11. Glassman SD, Alegre G, Carreon L, Dimar JR, Johnson JR. Perioperative complications of lumbar instrumentation and fusion in patients with diabetes mellitus. Spine J. 2003;3(6):496-501. Epub 2003/11/12. doi: 10.1016/s1529-9430(03)00426-1. PubMed PMID: 14609695.

12. Glassman SD, Anagnost SC, Parker A, Burke D, Johnson JR, Dimar JR. The effect of cigarette smoking and smoking cessation on spinal fusion. Spine (Phila Pa 1976). 2000;25(20):2608-15. Epub 2000/10/18. doi: 10.1097/00007632-200010150-00011. PubMed PMID: 11034645.

13. Hoffmann MF, Jones CB, Sietsema DL. Adjuncts in posterior lumbar spine fusion: comparison of complications and efficacy. Arch Orthop Trauma Surg. 2012;132(8):1105-10. Epub 2012/05/09. doi: 10.1007/s00402-012-1529-0. PubMed PMID: 22562366.

14. Hyun SJ, Yoon SH, Kim JH, Oh JK, Lee CH, Shin JJ, et al. A prospective, multi-center, double-blind, randomized study to evaluate the efficacy and safety of the synthetic bone graft material dbm gel with rhbmp-2 versus dbm gel used during the tlif procedure in patients with lumbar disc disease. Journal of Korean Neurosurgical Society. 2021;64(4):562‐74. doi: 10.3340/jkns.2020.0331. PubMed PMID: CN-02292564.

15. Joseph V, Rampersaud YR. Heterotopic bone formation with the use of rhBMP2 in posterior minimal access interbody fusion: a CT analysis. Spine (Phila Pa 1976). 2007;32(25):2885-90. Epub 2008/02/05. doi: 10.1097/BRS.0b013e31815b7596. PubMed PMID: 18246013.

16. Li Z, Li Z, Chen X, Han X, Li K, Li S. Comparison between modified facet joint fusion and posterolateral fusion for the treatment of lumbar degenerative diseases: a retrospective study. BMC surgery. 2022;22(1):29. doi: 10.1186/s12893-022-01468-4. PubMed PMID: CN-02365060.

17. Luszczyk M, Smith JS, Fischgrund JS, Ludwig SC, Sasso RC, Shaffrey CI, et al. Does smoking have an impact on fusion rate in single-level anterior cervical discectomy and fusion with allograft and rigid plate fixation? Clinical article. J Neurosurg Spine. 2013;19(5):527-31. Epub 2013/09/03. doi: 10.3171/2013.7.Spine13208. PubMed PMID: 23992432.

18. Moazzeni K, Kazemi KA, Khanmohammad R, Eslamian M, Rostami M, Faghih-Jouibari M. Comparison of Surgical Outcome Between Diabetic Versus Nondiabetic Patients After Lumbar Fusion. Int J Spine Surg. 2018;12(4):528-32. Epub 2018/10/03. doi: 10.14444/5064. PubMed PMID: 30276114; PubMed Central PMCID: PMCPMC6159760.

19. Niu S, Anastasio AT, Faraj RR, Rhee JM. Evaluation of Heterotopic Ossification After Using Recombinant Human Bone Morphogenetic Protein-2 in Transforaminal Lumbar Interbody Fusion: A Computed Tomography Review of 996 Disc Levels. Global Spine J. 2020;10(3):280-5. Epub 2020/04/22. doi: 10.1177/2192568219846074. PubMed PMID: 32313793; PubMed Central PMCID: PMCPMC7160810.

20. Nourian AA, Harrington J, Pulido PA, McCauley JC, Bruffey JD, Eastlack RK. Fusion Rates of Lateral Lumbar Interbody Fusion Using Recombinant Human Bone Morphogenetic Protein-2. Global Spine J. 2019;9(4):398-402. Epub 2019/06/21. doi: 10.1177/2192568218797097. PubMed PMID: 31218198; PubMed Central PMCID: PMCPMC6562218.

21. Phan K, Fadhil M, Chang N, Giang G, Gragnaniello C, Mobbs RJ. Effect of Smoking Status on Successful Arthrodesis, Clinical Outcome, and Complications After Anterior Lumbar Interbody Fusion (ALIF). World Neurosurg. 2018;110:e998-e1003. Epub 2017/12/11. doi: 10.1016/j.wneu.2017.11.157. PubMed PMID: 29223523.

22. Ravindra VM, Godzik J, Dailey AT, Schmidt MH, Bisson EF, Hood RS, et al. Vitamin D Levels and 1-Year Fusion Outcomes in Elective Spine Surgery: A Prospective Observational Study. Spine. 2015;40(19):1536-41. doi: 10.1097/BRS.0000000000001041.

23. Ravindra VM, Guan J, Holland CM, Dailey AT, Schmidt MH, Godzik J, et al. Vitamin D status in cervical spondylotic myelopathy: Comparison of fusion rates and patient outcome measures. Journal of Neurosurgical Sciences. 2019;63(1):36-41. doi: 10.23736/S0390-5616.16.03846-7.

24. Samartzis D, Shen FH, Matthews DK, Yoon ST, Goldberg EJ, An HS. Comparison of allograft to autograft in multilevel anterior cervical discectomy and fusion with rigid plate fixation. Spine J. 2003;3(6):451-9. Epub 2003/11/12. doi: 10.1016/s1529-9430(03)00173-6. PubMed PMID: 14609689.

25. Son HJ, Choi SH, Lee MK, Kang CN. Efficacy and safety of Escherichia coli-derived recombinant human bone morphogenetic protein-2 in additional lumbar posterolateral fusion: minimum 1-year follow-up. Spine J. 2021;21(8):1340-6. Epub 2021/04/14. doi: 10.1016/j.spinee.2021.04.007. PubMed PMID: 33848691.

26. Srour R, Gdoura Y, Delaitre M, Mortada J, Benali MA, Millot F, et al. Facet Arthrodesis with the FFX Device: One-Year Results from a Prospective Multicenter Study. Int J Spine Surg. 2020;14(6):996-1002. Epub 2021/02/10. doi: 10.14444/7149. PubMed PMID: 33560260; PubMed Central PMCID: PMCPMC7872413.

27. Suchomel P, Barsa P, Buchvald P, Svobodnik A, Vanickova E. Autologous versus allogenic bone grafts in instrumented anterior cervical discectomy and fusion: a prospective study with respect to bone union pattern. European spine journal. 2004;13(6):510‐5. doi: 10.1007/s00586-003-0667-z. PubMed PMID: CN-00527573.

28. Tan B, Wang H, Dong J, Yuan Z, Wang D, Wang F. Comparison of rhBMP-2 versus Autogenous Iliac Crest Bone Graft for 2-Level Anterior Cervical Discectomy and Fusion for Cervical Spondylotic Myelopathy. Med Sci Monit. 2015;21:3159-65. Epub 2015/10/21. doi: 10.12659/msm.894656. PubMed PMID: 26479708; PubMed Central PMCID: PMCPMC4617188.

29. Tan Y, Tanaka M, Sonawane S, Uotani K, Oda Y, Fujiwara Y, et al. Comparison of Simultaneous Single-Position Oblique Lumbar Interbody Fusion and Percutaneous Pedicle Screw Fixation with Posterior Lumbar Interbody Fusion Using O-arm Navigated Technique for Lumbar Degenerative Diseases. J Clin Med. 2021;10(21). Epub 2021/11/14. doi: 10.3390/jcm10214938. PubMed PMID: 34768459; PubMed Central PMCID: PMCPMC8584546.

30. Tannoury C, Bhale R, Vora M, Saade A, Kortbawi R, Orlando G, et al. Pseudarthrosis Following Lumbar and Lumbosacral Fusion Using the Antepsoas Technique. Spine (Phila Pa 1976). 2021;46(24):1690-5. Epub 2021/09/03. doi: 10.1097/brs.0000000000004115. PubMed PMID: 34474451.

31. Tuli SK, Tuli J, Chen P, Woodard EJ. Fusion rate: a time-to-event phenomenon. J Neurosurg Spine. 2004;1(1):47-51. Epub 2004/08/05. doi: 10.3171/spi.2004.1.1.0047. PubMed PMID: 15291020.

32. Urrutia J, Molina M. Fresh-frozen femoral head allograft as lumbar interbody graft material allows high fusion rate without subsidence. Orthop Traumatol Surg Res. 2013;99(4):413-8. Epub 2013/04/20. doi: 10.1016/j.otsr.2013.03.010. PubMed PMID: 23597870.

33. Wang H, Meng Y, Liu H, Wang X, Hong Y. The impact of smoking on outcomes following anterior cervical fusion-nonfusion hybrid surgery: a retrospective single-center cohort study. BMC Musculoskelet Disord. 2021;22(1):612. Epub 2021/07/11. doi: 10.1186/s12891-021-04501-4. PubMed PMID: 34243728; PubMed Central PMCID: PMCPMC8272305.

34. Weng F, Wang J, Yang L, Zeng J, Chu Y, Tian Z. Application value of expansive pedicle screw in the lumbar short-segment fixation and fusion for osteoporosis patients. Exp Ther Med. 2018;16(2):665-70. Epub 2018/08/17. doi: 10.3892/etm.2018.6248. PubMed PMID: 30112031; PubMed Central PMCID: PMCPMC6090430.

35. Wu FL, Dang L, Zhou H, Yu M, Wei F, Jiang L, et al. Two-Year Outcomes of Midline lumbar Fusion Versus Minimally Invasive Transforaminal Lumbar Interbody Fusion in the Treatment of L4-L5 Degenerative Disease. Biomed Environ Sci. 2020;33(11):839-48. Epub 2021/03/28. doi: 10.3967/bes2020.114. PubMed PMID: 33771237.

36. Wu ZX, Gong FT, Liu L, Ma ZS, Zhang Y, Zhao X, et al. A comparative study on screw loosening in osteoporotic lumbar spine fusion between expandable and conventional pedicle screws. Archives of orthopaedic and trauma surgery. 2012;132(4):471‐6. doi: 10.1007/s00402-011-1439-6. PubMed PMID: CN-00971299.

37. Yang Y, Ma L, Hong Y, Liu H, Song Y, Liu L, et al. The application of zero-profile implant in two-level and single level anterior cervical discectomy and fusion for the treatment of cervical spondylosis: A comparative study. International Journal of Clinical and Experimental Medicine. 2016;9(8):15667-77.

38. Zhang W, Li L, Zhou X, Li K, Liu C, Lin X, et al. Concurrent Treatment with Vitamin K2 and D3 on Spine Fusion in Patients with Osteoporosis-Associated Lumbar Degenerative Disorders. Spine. 2022;47(4):352‐60. doi: 10.1097/BRS.0000000000004309. PubMed PMID: CN-02365382.

39. Zhang YH, Shen L, Shao J, Chou D, Song J, Zhang J. Structural Allograft versus Autograft for Instrumented Atlantoaxial Fusions in Pediatric Patients: Radiologic and Clinical Outcomes in Series of 32 Patients. World Neurosurg. 2017;105:549-56. Epub 2017/06/19. doi: 10.1016/j.wneu.2017.06.048. PubMed PMID: 28624564.

40. Dipaola CP, Bible JE, Biswas D, Dipaola M, Grauer JN, Rechtine GR. Survey of spine surgeons on attitudes regarding osteoporosis and osteomalacia screening and treatment for fractures, fusion surgery, and pseudoarthrosis. Spine J. 2009;9(7):537-44. Epub 2009/03/31. doi: 10.1016/j.spinee.2009.02.005. PubMed PMID: 19328744.

41. Faundez A, Tournier C, Garcia M, Aunoble S, Le Huec JC. Bone morphogenetic protein use in spine surgery-complications and outcomes: a systematic review. Int Orthop. 2016;40(6):1309-19. Epub 2016/03/11. doi: 10.1007/s00264-016-3149-8. PubMed PMID: 26961193.

42. Fischer CR, Hanson G, Eller M, Lehman RA. A Systematic Review of Treatment Strategies for Degenerative Lumbar Spine Fusion Surgery in Patients With Osteoporosis. Geriatr Orthop Surg Rehabil. 2016;7(4):188-96. Epub 2016/11/17. doi: 10.1177/2151458516669204. PubMed PMID: 27847678; PubMed Central PMCID: PMCPMC5098690.

43. Fretes N, Vellios E, Sharma A, Ajiboye RM. Radiographic and functional outcomes of bisphosphonate use in lumbar fusion: a systematic review and meta-analysis of comparative studies. European Spine Journal. 2020;29(2):272-81. doi: 10.1007/s00586-019-06090-2.

44. Galimberti F, Lubelski D, Healy AT, Wang T, Abdullah KG, Nowacki AS, et al. A Systematic Review of Lumbar Fusion Rates With and Without the Use of rhBMP-2. Spine (Phila Pa 1976). 2015;40(14):1132-9. Epub 2015/05/09. doi: 10.1097/brs.0000000000000971. PubMed PMID: 25955186.

45. Heida K, Jr., Ebraheim M, Siddiqui S, Liu J. Effects on clinical outcomes of grafts and spacers used in transforaminal lumbar interbody fusion: a critical review. Orthop Surg. 2013;5(1):13-7. Epub 2013/02/20. doi: 10.1111/os.12026. PubMed PMID: 23420741; PubMed Central PMCID: PMCPMC6583142.

46. Hirsch BP, Unnanuntana A, Cunningham ME, Lane JM. The effect of therapies for osteoporosis on spine fusion: A systematic review. Spine Journal. 2013;13(2):190-9. doi: 10.1016/j.spinee.2012.03.035.

47. Hofstetter CP, Hofer AS, Levi AD. Exploratory meta-analysis on dose-related efficacy and morbidity of bone morphogenetic protein in spinal arthrodesis surgery. J Neurosurg Spine. 2016;24(3):457-75. Epub 2015/11/28. doi: 10.3171/2015.4.Spine141086. PubMed PMID: 26613283.

48. Hsieh PC, Chung AS, Brodke D, Park JB, Skelly AC, Brodt ED, et al. Autologous Stem Cells in Cervical Spine Fusion. Global Spine Journal. 2021;11(6):950-65. doi: 10.1177/2192568220948479.

49. Li Y, Zheng LM, Zhang ZW, He CJ. The Effect of Smoking on the Fusion Rate of Spinal Fusion Surgery: A Systematic Review and Meta-Analysis. World Neurosurg. 2021;154:e222-e35. Epub 2021/07/13. doi: 10.1016/j.wneu.2021.07.011. PubMed PMID: 34252631.

50. Li Z, Wang S, Xu G, Hu X, Han L, Zhao Y. Synergy effect of Sr and rhBMP-2: A potential solution to osteolysis caused by rhBMP-2. Med Hypotheses. 2020;144:109895. Epub 2020/06/12. doi: 10.1016/j.mehy.2020.109895. PubMed PMID: 32526512.

51. Lytle EJ, Lawless MH, Paik G, Tong D, Soo TM. The minimally effective dose of bone morphogenetic protein in posterior lumbar interbody fusion: a systematic review and meta-analysis. Spine J. 2020;20(8):1286-304. Epub 2020/04/28. doi: 10.1016/j.spinee.2020.04.012. PubMed PMID: 32339767.

52. Manzur M, Virk SS, Jivanelli B, Vaishnav AS, McAnany SJ, Albert TJ, et al. The rate of fusion for stand-alone anterior lumbar interbody fusion: a systematic review. Spine J. 2019;19(7):1294-301. Epub 2019/03/16. doi: 10.1016/j.spinee.2019.03.001. PubMed PMID: 30872148.

53. Deutsch H, Haid R, Rodts G, Jr., Mummaneni PV. The decision-making process: allograft versus autograft. Neurosurgery. 2007;60(1 Supp1 1):S98-102. Epub 2007/01/06. doi: 10.1227/01.Neu.0000249221.50085.Ad. PubMed PMID: 17204893.

54. Divi SN, Goyal DKC, Galetta MS, Fang T, Padua FG, Reyes AA, et al. How Does Body Mass Index Influence Outcomes in Patients after Lumbar Fusion? Spine. 2020;45(8):555-61. doi: 10.1097/BRS.0000000000003313.

55. Dorward IG, Buchowski JM, Stoker GE, Zebala LP. Posterior Cervical Fusion With Recombinant Human Bone Morphogenetic Protein-2: Complications and Fusion Rate at Minimum 2-Year Follow-Up. Clin Spine Surg. 2016;29(6):E276-81. Epub 2016/05/04. doi: 10.1097/BSD.0b013e318286fa7e. PubMed PMID: 27137152.

56. Eleswarapu A, Rowan FA, Le H, Wick JB, Roberto RF, Javidan Y, et al. Efficacy, Cost, and Complications of Demineralized Bone Matrix in Instrumented Lumbar Fusion: Comparison With rhBMP-2. Global Spine J. 2021;11(8):1223-9. Epub 2020/08/05. doi: 10.1177/2192568220942501. PubMed PMID: 32748702; PubMed Central PMCID: PMCPMC8453673.

57. Elgafy H, Wetzell B, Gillette M, Semaan H, Rowland A, Balboa CA, et al. Lumbar spine fusion outcomes using a cellular bone allograft with lineage-committed bone-forming cells in 96 patients. BMC Musculoskelet Disord. 2021;22(1):699. Epub 2021/08/19. doi: 10.1186/s12891-021-04584-z. PubMed PMID: 34404368; PubMed Central PMCID: PMCPMC8369686.

58. Epstein NE. An argument for traditional posterior cervical fusion techniques: evidence from 35 cases. Surg Neurol. 2008;70(1):45-51; discussion -2. Epub 2008/02/26. doi: 10.1016/j.surneu.2007.10.023. PubMed PMID: 18295832.

59. Erinç S, Talmaç MA, Kemah B, Özdemir MH. The effect of modic changes on the fusion rates of posterior interbody fusion surgery modic changes and posterior interbody fusion. J Neurosurg Sci. 2021. Epub 2021/08/04. doi: 10.23736/s0390-5616.21.05386-8. PubMed PMID: 34342195.

60. Franco SC, Munoz FAL. Utility of teriparatide in the aged spine surgery. Osteoporosis international. 2017;28:S234‐. doi: 10.1007/s00198-017-3950-2. PubMed PMID: CN-01409678.

61. Ge M, Zhang Y, Ying H, Feng C, Li Y, Tian J, et al. Comparison of hidden blood loss and clinical efficacy of percutaneous endoscopic transforaminal lumbar interbody fusion and minimally invasive transforaminal lumbar interbody fusion. Int Orthop. 2022;46(9):2063-70. Epub 2022/06/21. doi: 10.1007/s00264-022-05485-z. PubMed PMID: 35723702; PubMed Central PMCID: PMCPMC9372117.

62. Ghasemi AA. Transforaminal lumbar interbody fusion versus instrumented posterolateral fusion In degenerative spondylolisthesis: An attempt to evaluate the superiority of one method over the other. Clin Neurol Neurosurg. 2016;150:1-5. Epub 2016/08/27. doi: 10.1016/j.clineuro.2016.08.017. PubMed PMID: 27565009.

63. Glassman SD, Carreon L, Djurasovic M, Campbell MJ, Puno RM, Johnson JR, et al. Posterolateral lumbar spine fusion with INFUSE bone graft. Spine J. 2007;7(1):44-9. Epub 2007/01/02. doi: 10.1016/j.spinee.2006.06.381. PubMed PMID: 17197332.

64. Groff MW, Dailey AT, Ghogawala Z, Resnick DK, Watters WC, 3rd, Mummaneni PV, et al. Guideline update for the performance of fusion procedures for degenerative disease of the lumbar spine. Part 12: pedicle screw fixation as an adjunct to posterolateral fusion. J Neurosurg Spine. 2014;21(1):75-8. Epub 2014/07/02. doi: 10.3171/2014.4.Spine14277. PubMed PMID: 24980589.

65. Hall JM, Wolfe PN, Choma TJ, Goldstein CL. Adverse events following lumbar spine fusion: the combined effect of age, smoking and vitamin D. Spine Journal. 2016;16(10):S192. doi: 10.1016/j.spinee.2016.07.098.

66. Hamilton DK, Smith JS, Reames DL, Williams BJ, Shaffrey CI. Use of recombinant human bone morphogenetic protein-2 as an adjunct for instrumented posterior arthrodesis in the occipital cervical region: An analysis of safety, efficacy, and dosing. J Craniovertebr Junction Spine. 2010;1(2):107-12. Epub 2011/05/17. doi: 10.4103/0974-8237.77674. PubMed PMID: 21572631; PubMed Central PMCID: PMCPMC3075826.

67. Hassanzadeh H, Cancienne J, Werner BC, Yang S, Singla A, Shen FH, et al. Super obesity (BMI >50kg/m2) and complications after posterior lumbar spine fusion. Spine Journal. 2015;15(10):195S. doi: 10.1016/j.spinee.2015.07.260.

68. Hoffmann MF, Jones CB, Sietsema DL. Recombinant Human Bone Morphogenetic Protein-2 in Posterolateral Spinal Fusion: What's the Right Dose? Asian Spine J. 2016;10(3):457-64. Epub 2016/06/25. doi: 10.4184/asj.2016.10.3.457. PubMed PMID: 27340524; PubMed Central PMCID: PMCPMC4917763.

69. Hood B, Hamilton DK, Smith JS, Dididze M, Shaffrey C, Levi AD. The use of allograft and recombinant human bone morphogenetic protein for instrumented atlantoaxial fusions. World Neurosurg. 2014;82(6):1369-73. Epub 2013/01/23. doi: 10.1016/j.wneu.2013.01.083. PubMed PMID: 23336983.

70. Hsieh PC, Buser Z, Skelly AC, Brodt ED, Brodke D, Meisel HJ, et al. Allogenic Stem Cells in Spinal Fusion: A Systematic Review. Global Spine J. 2019;9(1 Suppl):22s-38s. Epub 2019/06/04. doi: 10.1177/2192568219833336. PubMed PMID: 31157144; PubMed Central PMCID: PMCPMC6512196.

71. Hu B, Song Y, Yang X. Comparison of titanium and n-HA/PA66 cages after anterior cervical fusion with single-level corpectomy over 8-year follow-up. Global spine journal. 2018;8(1):236S‐. doi: 10.1177/2192568218771072. PubMed PMID: CN-01606033.

72. Im SK, Lee JH, Lee KY, Yoo SJ. Effectiveness and Feasibility of Injectable Escherichia coli-Derived Recombinant Human Bone Morphogenetic Protein-2 for Anterior Lumbar Interbody Fusion at the Lumbosacral Junction in Adult Spinal Deformity Surgery: A Clinical Pilot Study. Orthop Surg. 2022;14(7):1350-8. Epub 2022/05/29. doi: 10.1111/os.13303. PubMed PMID: 35633034; PubMed Central PMCID: PMCPMC9251273.

73. Ishihara M, Taniguchi S, Kawashima K, Adachi T, Paku M, Tani Y, et al. Bone Fusion Morphology after Circumferential Minimally Invasive Spine Surgery Using Lateral Lumbar Interbody Fusion and Percutaneous Pedicle Screws without Bone Grafting in the Thoracic Spine: A Retrospective Study. Medicina (Kaunas). 2022;58(4). Epub 2022/04/24. doi: 10.3390/medicina58040496. PubMed PMID: 35454335; PubMed Central PMCID: PMCPMC9031519.

74. Jensen L, Peroutka R, Shaffrey M, Mason MacEnski M, Murar J. Trabecular metalbeta cervical interbody fusion device versus bone graft for one-level plated anterior cervical fusion: a prospective study. Spine journal. 2011;11(10 SUPPL. 1):122S‐3S. doi: 10.1016/j.spinee.2011.08.301. PubMed PMID: CN-01021046.

75. Jiao J, Li J, Luo Y, Zhang W. Clinical and radiographic outcomes of hybrid graft in patients with Modic changes undergoing transforaminal lumbar interbody fusion. J Orthop Surg Res. 2021;16(1):486. Epub 2021/08/13. doi: 10.1186/s13018-021-02652-7. PubMed PMID: 34380501; PubMed Central PMCID: PMCPMC8356436.

76. Ni B, Zhou F, Xie N, Guo X, Yang L, Guo Q, et al. Transarticular screw and C1 hook fixation for os odontoideum with atlantoaxial dislocation. World Neurosurg. 2011;75(3-4):540-6. Epub 2011/05/24. doi: 10.1016/j.wneu.2010.07.021. PubMed PMID: 21600509.

77. Park MS, Ju YS, Moon SH, Kim TH, Oh JK, Lim JK, et al. Repeat decompression and fusions following posterolateral fusion versus posterior/transforaminal lumbar interbody fusion for lumbar spondylosis: a national database study. Sci Rep. 2019;9(1):4926. Epub 2019/03/22. doi: 10.1038/s41598-019-41366-z. PubMed PMID: 30894618; PubMed Central PMCID: PMCPMC6426932.

78. Perdomo-Pantoja A, Shamoun F, Holmes C, Ishida W, Ramhmdani S, Cottrill E, et al. A retrospective cohort analysis of the effects of renin-angiotensin system inhibitors on spinal fusion in ACDF patients. Spine J. 2019;19(8):1354-61. Epub 2019/05/07. doi: 10.1016/j.spinee.2019.04.017. PubMed PMID: 31059820.

79. Pimenta L, Marchi L, Oliveira L, Coutinho E, Amaral R. A prospective, randomized, controlled trial comparing radiographic and clinical outcomes between stand-alone lateral interbody lumbar fusion with either silicate calcium phosphate or rh-BMP2. Journal of neurological surgery Part A, Central European neurosurgery. 2013;74(6):343‐50. doi: 10.1055/s-0032-1333420. PubMed PMID: CN-00909394.

80. Roh YH, Lee JC, Cho HK, Jang HD, Choi SW, Shin BJ. Comparative Study of Radiological and Clinical Outcomes in Patients Undergoing Minimally Invasive Lateral Lumbar Interbody Fusion Using Demineralized Bone Matrix Alone or with Low-Dose Escherichia coli-Derived rhBMP-2. World Neurosurg. 2022;158:e557-e65. Epub 2021/11/15. doi: 10.1016/j.wneu.2021.11.028. PubMed PMID: 34775087.

81. Schafer E, Bazydlo M, Schultz L, Park P, Chang V, Easton RW, et al. Rates and risk factors associated with 90-day readmission following cervical spine fusion surgery: analysis of the Michigan Spine Surgery Improvement Collaborative (MSSIC) registry. Spine Journal. 2020;20(5):708-16. doi: 10.1016/j.spinee.2020.01.003.

82. Schizas C, Triantafyllopoulos D, Kosmopoulos V, Tzinieris N, Stafylas K. Posterolateral lumbar spine fusion using a novel demineralized bone matrix: a controlled case pilot study. Arch Orthop Trauma Surg. 2008;128(6):621-5. Epub 2007/11/06. doi: 10.1007/s00402-007-0495-4. PubMed PMID: 17978826.

83. Schmitt PJ, Kelleher JP, Ailon T, Heller JE, Kasliwal MK, Shaffrey CI, et al. Long-Segment Fusion for Adult Spinal Deformity Correction Using Low-Dose Recombinant Human Bone Morphogenetic Protein-2: A Retrospective Review of Fusion Rates. Neurosurgery. 2016;79(2):212-21. Epub 2015/12/26. doi: 10.1227/neu.0000000000001194. PubMed PMID: 26702838.

84. Sclafani JA, Bergen SR, Staples M, Liang K, Raiszadeh R. Arthrodesis rate and patient reported outcomes after anterior lumbar interbody fusion utilizing a plasma-sprayed titanium coated PEEK interbody implant: a retrospective, observational analysis. International journal of spine surgery. 2017;11(1):17‐23. doi: 10.14444/4004. PubMed PMID: CN-01340835.

85. Seki S, Hirano N, Kawaguchi Y, Nakano M, Yasuda T, Suzuki K, et al. Teriparatide versus low-dose bisphosphonates before and after surgery for adult spinal deformity in female Japanese patients with osteoporosis. European spine journal. 2017:1‐7. doi: 10.1007/s00586-017-4959-0. PubMed PMID: CN-01333571.

86. Slosar PJ, Josey R, Reynolds J. Accelerating lumbar fusions by combining rhBMP-2 with allograft bone: a prospective analysis of interbody fusion rates and clinical outcomes. Spine J. 2007;7(3):301-7. Epub 2007/05/08. doi: 10.1016/j.spinee.2006.10.015. PubMed PMID: 17482113.

87. Truong VT, Sunna T, Al-Shakfa F, Mc Graw M, Boubez G, Shedid D, et al. Impact of obesity on complications and surgical outcomes of adult degenerative scoliosis with long-segment spinal fusion. Neurochirurgie. 2022;68(3):289-92. doi: 10.1016/j.neuchi.2021.12.005.

88. Burkus JK, Gornet MF, Dickman CA, Zdeblick TA. Anterior lumbar interbody fusion using rhBMP-2 with tapered interbody cages. Journal of spinal disorders. 2002;15(5):337‐49. PubMed PMID: CN-00410770.

89. Cho JH, Lee JH, Yeom JS, Chang BS, Yang JJ, Koo KH, et al. Efficacy of Escherichia coli-derived recombinant human bone morphogenetic protein-2 in posterolateral lumbar fusion: an open, active-controlled, randomized, multicenter trial. Spine journal. 2017;17(12):1866‐74. doi: 10.1016/j.spinee.2017.06.023. PubMed PMID: CN-01615775.

90. Choi HY, Hyun SJ, Lee CH, Youn JH, Ryu MY, Kim KJ. Safety and Efficacy of Recombinant Human Bone Morphogenetic Protein-2 in Multilevel Posterolateral Lumbar Fusion in a Prospective, Randomized, Controlled Trial. Neurospine. 2022;19(3):838‐46. doi: 10.14245/ns.2244464.232. PubMed PMID: CN-02470263.

91. Delawi D, Dhert WJ, Rillardon L, Gay E, Prestamburgo D, Garcia-Fernandez C, et al. A prospective, randomized, controlled, multicenter study of osteogenic protein-1 in instrumented posterolateral fusions: report on safety and feasibility. Spine. 2010;35(12):1185‐91. doi: 10.1097/BRS.0b013e3181d3cf28. PubMed PMID: CN-00767401.

92. Delawi D, Jacobs W, van Susante JL, Rillardon L, Prestamburgo D, Specchia N, et al. OP-1 Compared with Iliac Crest Autograft in Instrumented Posterolateral Fusion: a Randomized, Multicenter Non-Inferiority Trial. Journal of bone and joint surgery American volume. 2016;98(6):441‐8. doi: 10.2106/JBJS.O.00209. PubMed PMID: CN-01168074.

93. Dimar JR, Glassman SD, Burkus JK, Pryor PW, Hardacker JW, Carreon LY. Clinical and radiographic analysis of an optimized rhBMP-2 formulation as an autograft replacement in posterolateral lumbar spine arthrodesis. Journal of bone and joint surgery American volume. 2009;91(6):1377‐86. doi: 10.2106/JBJS.H.00200. PubMed PMID: CN-00697255.

94. Dimar JR, Glassman SD, Burkus KJ, Carreon LY. Clinical outcomes and fusion success at 2 years of single-level instrumented posterolateral fusions with recombinant human bone morphogenetic protein-2/compression resistant matrix versus iliac crest bone graft. Spine. 2006;31(22):2534‐9; discussion 40. doi: 10.1097/01.brs.0000240715.78657.81. PubMed PMID: CN-00572895.

95. Hu MH, Tseng YK, Chung YH, Wu NY, Li CH, Lee PY. The efficacy of oral vitamin D supplements on fusion outcome in patients receiving elective lumbar spinal fusion-a randomized control trial. BMC musculoskeletal disorders. 2022;23(1):996. doi: 10.1186/s12891-022-05948-9. PubMed PMID: CN-02499693.

96. Hurlbert RJ, Alexander D, Bailey S, Mahood J, Abraham E, McBroom R, et al. rhBMP-2 for posterolateral instrumented lumbar fusion: a multicenter prospective randomized controlled trial. Spine. 2013;38(25):2139‐48. doi: 10.1097/BRS.0000000000000007. PubMed PMID: CN-00910919.

97. Martínez-Gutiérrez O, Peña-Martínez V, Camacho-Ortiz A, Vilchez-Cavazos F, Simental-Mendía M, Tamez-Mata Y, et al. Spondylodiscitis treated with freeze-dried bone allograft alone or combined with autograft: a randomized and blinded trial. Journal of orthopaedic surgery (Hong Kong). 2021;29(2):23094990211019101. doi: 10.1177/23094990211019101. PubMed PMID: CN-02287210.

98. Schröder J, Grosse-Dresselhaus F, Schul C, Wassmann H. PMMA versus titanium cage after anterior cervical discectomy - a prospective randomized trial. Zentralblatt fur Neurochirurgie. 2007;68(1):2‐7. doi: 10.1055/s-2006-942184. PubMed PMID: CN-00585902.

99. Thalgott JS, Fogarty ME, Giuffre JM, Christenson SD, Epstein AK, Aprill C. A prospective, randomized, blinded, single-site study to evaluate the clinical and radiographic differences between frozen and freeze-dried allograft when used as part of a circumferential anterior lumbar interbody fusion procedure. Spine (Phila Pa 1976). 2009;34(12):1251-6. Epub 2009/05/05. doi: 10.1097/BRS.0b013e3181a005d7. PubMed PMID: 19412139.

100. Thomé C, Leheta O, Krauss JK, Zevgaridis D. A prospective randomized comparison of rectangular titanium cage fusion and iliac crest autograft fusion in patients undergoing anterior cervical discectomy. Journal of neurosurgery Spine. 2006;4(1):1‐9. doi: 10.3171/spi.2006.4.1.1. PubMed PMID: CN-00555452.

101. Tian Y, Liu X. Clinical outcomes of two minimally invasive transforaminal lumbar interbody fusion (TLIF) for lumbar degenerative diseases. European journal of orthopaedic surgery & traumatology : orthopedie traumatologie. 2016;26(7):745‐51. doi: 10.1007/s00590-016-1755-1. PubMed PMID: CN-01719833.

102. Vaccaro AR, Anderson DG, Patel T, Fischgrund J, Truumees E, Herkowitz HN, et al. Comparison of OP-1 Putty (rhBMP-7) to iliac crest autograft for posterolateral lumbar arthrodesis: a minimum 2-year follow-up pilot study. Spine. 2005;30(24):2709‐16. doi: 10.1097/01.brs.0000190812.08447.ba. PubMed PMID: CN-00561814.

103. Vaccaro AR, Patel T, Fischgrund J, Anderson DG, Truumees E, Herkowitz HN, et al. A pilot study evaluating the safety and efficacy of OP-1 Putty (rhBMP-7) as a replacement for iliac crest autograft in posterolateral lumbar arthrodesis for degenerative spondylolisthesis. Spine. 2004;29(17):1885‐92. doi: 10.1097/01.brs.0000137062.79201.98. PubMed PMID: CN-00527684.

104. Vaccaro AR, Whang PG, Patel T, Phillips FM, Anderson DG, Albert TJ, et al. The safety and efficacy of OP-1 (rhBMP-7) as a replacement for iliac crest autograft for posterolateral lumbar arthrodesis: minimum 4-year follow-up of a pilot study. Spine journal. 2008;8(3):457‐65. doi: 10.1016/j.spinee.2007.03.012. PubMed PMID: CN-00702024.

105. Xue H, Tu Y, Cai M. Comparison of unilateral versus bilateral instrumented transforaminal lumbar interbody fusion in degenerative lumbar diseases. Spine journal. 2012;12(3):209‐15. doi: 10.1016/j.spinee.2012.01.010. PubMed PMID: CN-00970376.

106. 阿尖措, 王喜民, 李占银, 许志华. 解剖型表面微孔钛合金椎间融合器的生物相容性. 中国组织工程研究. 2016;20(48):7185-91. doi: 10.3969/j.issn.2095-4344.2016.48.006.

107. 莫建强. 解剖型表面微孔钛合金椎间融合器治疗腰椎退变性疾病的疗效研究 [硕士]: 苏州大学; 2009.

108. 盛珺, 刘达, 郑伟, 周江军, 伍红桦, 徐伟, et al. 唑来膦酸联合骨水泥钉道强化椎弓根螺钉用于骨质疏松腰椎融合术的疗效. 解放军医学杂志. 2018;43(12):1044-8. doi: 10.11855/j.issn.0577-7402.2018.12.09.

109. Feng C, Bo Y, Duo Z, Qincan C, Qi L. Cortical bone trajectory in elderly patients with osteoporosis of lumbar disease. Chinese journal of tissue engineering research. 2022;26(3):419‐23. doi: 10.12307/2022.066. PubMed PMID: CN-02301048.

110. Ferrete-Barroso AM, González-Díaz R, Losada-Viñas JI. Anterior cervical arthrodesis using a vertebral body autograft. Rev Esp Cir Ortop Traumatol. 2015;59(3):172-8. Epub 2014/12/03. doi: 10.1016/j.recot.2014.09.003. PubMed PMID: 25450161.

111. Hu W, Kan S, Cao Z, Jiang Z, Zhang X, Zhu R. Artificial tiger bone and calfor applied in intervertebral fusion after degenerative lumbar spondylolisthesis. Chinese journal of tissue engineering research. 2018;22(36):5741‐5. doi: 10.3969/j.issn.2095-4344.0615. PubMed PMID: CN-02162164.

112. Song CZ, Jin HX. Biocompatibility of carbon fiber interbody fusion cage versus titanium mesh cage in treatment of lumbar disc degeneration. Chinese journal of tissue engineering research. 2015;19(12):1909‐13. doi: 10.3969/j.issn.2095-4344.2015.12.020. PubMed PMID: CN-01129387.

113. Vincentelli AF, Szadkowski M, Vardon D, Litrico S, Fuentès S, Steib J-P, et al. Description des utilisations de rhBMP 2 dans la chirurgie du rachis : étude rétrospective multicentrique française. Revue de Chirurgie Orthopédique et Traumatologique. 2016;102(7). doi: 10.1016/j.rcot.2016.08.192.

114. Drakopoulos P, Flevas DA, Galanopoulos IP, Lepetsos P, Zafeiris C. Off-Label Use of Teriparatide in Spine. Cureus. 2021;13(7):e16522. Epub 2021/08/26. doi: 10.7759/cureus.16522. PubMed PMID: 34430132; PubMed Central PMCID: PMCPMC8376240.

115. Glaeser JD, Salehi K, Kanim LE, Ju DG, Hyuk Yang J, Behrens PH, et al. Electrospun, synthetic bone void filler promotes human MSC function and BMP-2 mediated spinal fusion. J Biomater Appl. 2020;35(4-5):532-43. Epub 2020/07/07. doi: 10.1177/0885328220937999. PubMed PMID: 32627633; PubMed Central PMCID: PMCPMC7885703.

116. Glassman SD, Dimar JR, Carreon LY, Campbell MJ, Puno RM, Johnson JR. Initial fusion rates with recombinant human bone morphogenetic protein-2/compression resistant matrix and a hydroxyapatite and tricalcium phosphate/collagen carrier in posterolateral spinal fusion. Spine (Phila Pa 1976). 2005;30(15):1694-8. Epub 2005/08/12. doi: 10.1097/01.brs.0000172157.39513.80. PubMed PMID: 16094268.

117. Glazer PA, Spencer UM, Alkalay RN, Schwardt J. In vivo evaluation of calcium sulfate as a bone graft substitute for lumbar spinal fusion. Spine J. 2001;1(6):395-401. Epub 2003/11/01. doi: 10.1016/s1529-9430(01)00108-5. PubMed PMID: 14588295.

118. Hsu EL, Sonn K, Kannan A, Bellary S, Yun C, Hashmi S, et al. Dioxin Exposure Impairs BMP-2-Mediated Spinal Fusion in a Rat Arthrodesis Model. J Bone Joint Surg Am. 2015;97(12):1003-10. Epub 2015/06/19. doi: 10.2106/jbjs.N.01311. PubMed PMID: 26085534.
