# Supplementary material for: Risk factors affecting spinal fusion: A meta-analysis of 39 cohort studies
Source: PLoS One. 2024 Jun 7;19(6):e0304473. doi: 10.1371/journal.pone.0304473 (PMC11161075; doi:10.1371/journal.pone.0304473)
Supplement: S5 Table — (DOCX) [file pone.0304473.s007.docx]

**S5 Table.** Fusion Rate by Study-level Factors.

| Variable | Rate, % | 95% CI | Degree of heterogeneity  (I² statistics; %) | *P* value | No. of included Studies | *P* for interaction |
| --- | --- | --- | --- | --- | --- | --- |
| **Total** | **89.2** | **87.4-91.1** | **86.9** | **<0.001** | **39** | **<0.001** |
| Study design |  |  |  |  |  | <0.001 |
| Retrospective | 92.0 | 90.2-93.8 | 81.2 | <0.001 | 23 |  |
| Prospective | 84.2 | 79.7-88.6 | 88.9 | <0.001 | 16 |  |
| Regions |  |  |  |  |  | <0.001 |
| China | 89.6 | 86.0-93.3 | 71.0 | 0.001 | 9 |  |
| USA | 89.3 | 86.4-92.1 | 91.1 | <0.001 | 19 |  |
| others | 88.6 | 85.1-92.2 | 82.3 | <0.001 | 11 |  |
| Sample size |  |  |  |  |  | <0.001 |
| <100 | 89.7 | 87.0-92.4 | 75.1 | <0.001 | 21 |  |
| 100-500 | 90.1 | 89.1-91.1 | 93.7 | <0.001 | 14 |  |
| >500 | 92.1 | 91.2-93.0 | 0 | 0.593 | 4 |  |
| Study year |  |  |  |  |  | <0.001 |
| <2015 | 89.3 | 86.3-92.2 | 86.2 | <0.001 | 19 |  |
| 2015-2020 | 87.0 | 82.8-91.2 | 84.1 | <0.001 | 10 |  |
| >2020 | 91.4 | 88.4-94.5 | 81.9 | <0.001 | 10 |  |
| Mean follow-up period | | |  |  |  | <0.001 |
| <24 months | 88.7 | 85.7-91.0 | 86.1 | <0.001 | 18 |  |
| 24-48 months | 88.5 | 85.1-91.5 | 90.2 | <0.001 | 16 |  |
| >48 months | 92.6 | 91.4-93.9 | 0 | 0.509 | 5 |  |
| Female % |  |  |  |  |  | <0.001 |
| <50 | 85.1 | 79.4-90.8 | 91.7 | <0.001 | 13 |  |
| ≥50 | 91.1 | 89.2-93.1 | 80.6 | <0.001 | 22 |  |
| NR | 91.6 | 89.4-91.9 | 37.3 | 0.188 | 4 |  |
| Surgical sites |  |  |  |  |  | <0.001 |
| Lumbar | 87.8 | 85.2-90.5 | 89.9 | <0.001 | 25 |  |
| Cervical | 92.0 | 89.7-94.3 | 73.7 | <0.001 | 13 |  |
| Surgical types |  |  |  |  |  | <0.001 |
| TLIF or PLIF | 85.5 | 81.2-89.7 | 92.4 | <0.001 | 12 |  |
| LLIF | 95.3 | 90.8-99.7 | 70.2 | 0.035 | 3 |  |
| ALIF | 86.3 | 81.1-91.5 | 42.6 | 0.175 | 3 |  |
| ACDF | 92.3 | 90.1-94.5 | 71.3 | <0.001 | 11 |  |
| Others | 88.2 | 83.5-92.9 | 85.4 | <0.001 | 10 |  |

Abbreviations: ACDF, Anterior cervical discectomy and fusion; ALIF, Anterior lumbar interbody fusion; CI, confidence interval; LLIF, Lateral lumbar interbody fusion; NR, not reported; PLIF, posterior lumbar interbody fusion; TLIF, transforaminal lumbar interbody fusion.
