# Supplementary material for: Risk factors affecting spinal fusion: A meta-analysis of 39 cohort studies
Source: PLoS One. 2024 Jun 7;19(6):e0304473. doi: 10.1371/journal.pone.0304473 (PMC11161075; doi:10.1371/journal.pone.0304473)
Supplement: S6 Table — (DOCX) [file pone.0304473.s008.docx]

**S6 Table.** Sensitivity Analysis for Significant and Non-significant Factors and Class of Evidence.

| **Significant factors** | **Sample size** | **OR (95% CI)** | **T-F adjusted OR (95% CI)** | **Filled studies** | **Class of Evidence** |
| --- | --- | --- | --- | --- | --- |
| Smoking |  |  |  |  |  |
| No |  | Ref. |  |  |  |
| Yes | 1672 | 1.57 (1.11 to 2.21) | 1.57 (1.11 to 2.21) | 0 | Ⅰ |
|  |  |  |  |  |  |
| Graft type |  |  |  |  |  |
| Autograft |  | Ref. |  |  |  |
| Allograft | 460 | 1.82 (1.11 to 2.96) | 1.28 (0.79 to 2.07) | 3 | Ⅲ |
|  |  |  |  |  |  |
| Without the use of BMP-2 |  |  |  |  |  |
| No |  | Ref. |  |  |  |
| Yes | 3802 | 4.42 (3.33 to 5.86) | 3.58 (2.70 to 4.75) | 1 | Ⅰ |
|  |  |  |  |  |  |
| Vitamin D deficiency |  |  |  |  |  |
| No |  | Ref. |  |  |  |
| Yes | 260 | 2.46 (1.24 to 4.90) | 2.46 (1.24 to 4.90) | 0 | Ⅱ |
|  |  |  |  |  |  |
| Pedicle screw type |  |  |  |  |  |
| EPS |  | Ref. |  |  |  |
| CPS | 237 | 4.77 (2.23 to 10.20) | 2.98 (1.56 to 5.71) | 1 | Ⅲ |
|  |  |  |  |  |  |
| Diabetes |  |  |  |  |  |
| No |  | Ref. |  |  |  |
| Yes | 233 | 3.42 (1.59 to 7.36) | 2.71 (1.36 to 5.41) | 1 | Ⅲ |
|  |  |  |  |  |  |
| Fusion column |  |  |  |  |  |
| Lateral |  | Ref. |  |  |  |
| Posterolateral | 130 | 3.63 (1.25 to 10.49) | 2.40 (0.98 to 5.88) | 1 | Ⅲ |
| **Non-significant factors** | **Sample size** | **OR (95% CI)** | **T-F adjusted OR (95% CI)** | **Filled studies** | **Class of Evidence** |
| Number of fused levels |  |  |  |  |  |
| Single |  | Ref. |  |  |  |
| Two | 282 | 0.93 (0.36 to 2.41) | 0.93 (0.36 to 2.41) | 0 | Ⅲ |
|  |  |  |  |  |  |
| MIS |  |  |  |  |  |
| No |  | Ref. |  |  |  |
| Yes | 148 | 1.92 (0.43 to 8.66) | 1.85 (0.53 to 6.46) | 1 | Ⅲ |

Abbreviations: BMP-2, bone morphogenetic protein-2; CI, confidence interval; CPS, conventional pedicle screws; EPS, expandable pedicle screws; MIS, minimally invasive surgery; OR, odds ratio; Ref, Reference group; T-F, trim and fill method.
