# Supplementary material for: Risk factors affecting spinal fusion: A meta-analysis of 39 cohort studies
Source: PLoS One. 2024 Jun 7;19(6):e0304473. doi: 10.1371/journal.pone.0304473 (PMC11161075; doi:10.1371/journal.pone.0304473)
Supplement: S7 Table — (DOCX) [file pone.0304473.s009.docx]

**S7 Table.** Sensitivity analysis for fusion rates associated with patient-related and surgery-related factors.

| **Study omitted** | **Year** | **Pooled OR (95% CI)** | **Weight** | **Heterogeneity estimate,**  **I², % (P value)** |
| --- | --- | --- | --- | --- |
| **Patient-related factors** |  |  |  |  |
| **Vitamin D deficiency** |  |  |  |  |
| Ravindra et al | 2015 | 2.01(0.77,5.26) | 48.86 | 48.80%,0.162 |
| Zhang et al | 2022 | 2.10(0.95,4.62) | 24.31 | 38.50%,0.202 |
| Ravindra et al | 2019 | 3.39(1.52,7.56) | 26.83 | 0.00%,0.733 |
| **Overall** |  | **2.46(1.24,4.90)** |  | **13.50%,0.010** |
|  |  |  |  |  |
| **Smoking** |  |  |  |  |
| Phan et al | 2018 | 1.47(1.01,2.13) | 11.54 | 0.00%,0.750 |
| Luszczyk et al | 2013 | 1.81(1.20,2.74) | 28.81 | 0.00%,0.851 |
| Tannoury et al | 2021 | 1.53(1.07,2.19) | 4.67 | 0.00%,0.655 |
| Suchomel et al | 2004 | 1.55(1.08,2.21) | 4.29 | 0.00%,0.632 |
| Urrutia et al | 2013 | 1.58(1.11,2.25) | 2.31 | 0.00%,0.656 |
| Glassman et al | 2000 | 1.55(0.99,2.43) | 39.7 | 0.00%,0.627 |
| Bose et al | 2001 | 1.59(1.12,2.26) | 2.12 | 0.00%,0.700 |
| Wang et al | 2021 | 1.47(1.03,2.12) | 6.57 | 0.00%,0.819 |
| **Overall** |  | **1.57(1.11,2.11)** |  | **0.00%,0.010** |
|  |  |  |  |  |
| **Surgery-related factors** |  |  |  |  |
| **Without the use of BMP-2** |  |  |  |  |
| Niu et al | 2020 | 3.46(2.49,4.79) | 22.86 | 33.10%,0.134 |
| Tan et al | 2015 | 3.94(2.91,5.33) | 10.82 | 33.60%,0.130 |
| Adams et al | 2014 | 3.81(2.85,5.09) | 2.34 | 35.40%,0.116 |
| Burkus et al | 2004 | 3.68(2.76,4.91) | 0.95 | 30.90%,0.153 |
| Burkus et al | 2017 | 3.58(2.68,4.78) | 2.08 | 11.70%,0.332 |
| Burkus et al | 2005 | 3.62(2.71,4.83) | 1.90 | 22.80%,0.226 |
| Son et al | 2021 | 3.75(2.81,4.99) | 0.87 | 37.50%,0.100 |
| Hoffmann et al | 2012 | 3.88(2.61,5.76) | 47.77 | 37.30%,0.101 |
| Joseph et al | 2007 | 3.80(2.85,5.07) | 1.27 | 34.20%,0.125 |
| Frenkel et al | 2013 | 3.71(2.79,4.95) | 0.92 | 35.70%,0.113 |
| Gerszten et al | 2011 | 3.83(2.87,5.12) | 2.69 | 34.30%,0.124 |
| Hyun et al | 2021 | 3.98(2.96,5.34) | 5.53 | 23.90%,0.216 |
| **Overall** |  | **4.42(3.33,5.86)** |  | **36.50%,0.000** |
|  |  |  |  |  |
| **Allograft** |  |  |  |  |
| Samartzis et al | 2003 | 1.59(0.94,2.69) | 2.85 | 31.40%,0.212 |
| Zhang et al | 2017 | 1.64(0.97,2.77) | 2.46 | 31.50%,0.158 |
| Suchomel et al | 2004 | 1.54(0.89,2.67) | 10.94 | 34.10%,0.194 |
| Bishop et al | 1996 | 1.45(0.84,2.50) | 9.89 | 6.80%,0.368 |
| Frantzén et al | 2011 | 1.57(0.93,2.65) | 3.06 | 23.20%,0.267 |
| Cammisa Jr et al | 2004 | 4.47(1.72,11.64) | 70.80 | 0.00%,0.936 |
| **Overall** |  | **1.82(1.11,2.96)** |  | **25.70%,0.018** |
|  |  |  |  |  |
| **Two-level fusion** |  |  |  |  |
| Nourian et al | 2019 | 0.80(0.21,3.12) | 45.80 | 49.20%,0.160 |
| Tuli et al | 2004 | 1.55(0.53,4.51) | 12.83 | 0.00%,0.845 |
| Yang et al | 2016 | 0.99(0.27,3.65) | 41.37 | 58.50%,0.120 |
| **Overall** |  | **0.93(0.36,2.41)** |  | **20.50%,0.887** |

Abbreviations: BMP-2, bone morphogenetic protein-2; CI, confidence interval; MIS, minimally invasive surgery; OR, odds ratio.
