# Supplementary figures and images for: Risk factors affecting spinal fusion: A meta-analysis of 39 cohort studies
Source: PLoS One. 2024 Jun 7;19(6):e0304473. doi: 10.1371/journal.pone.0304473 (PMC11161075; doi:10.1371/journal.pone.0304473)

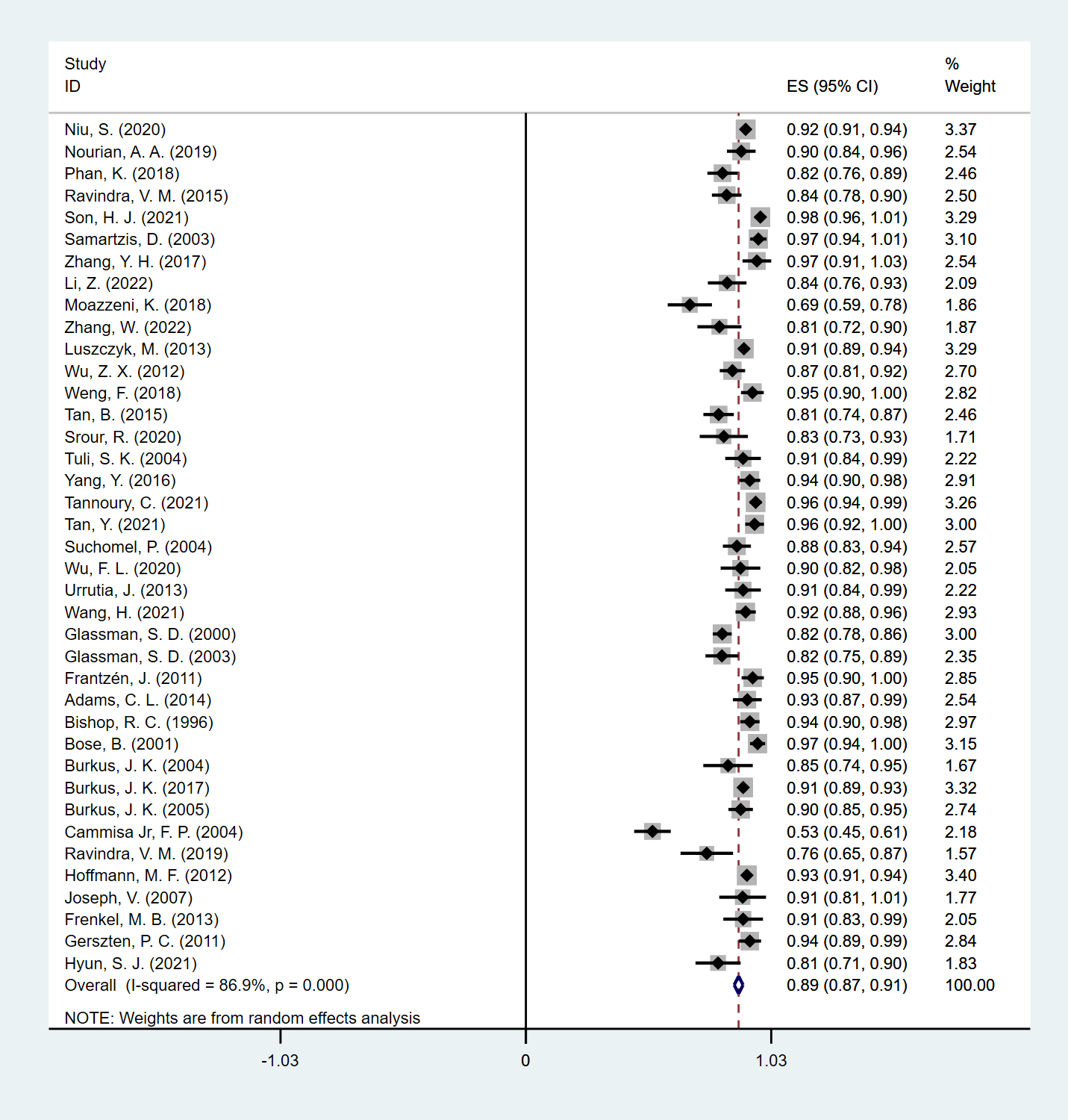

Supplement: S1 Fig — (TIF) [file pone.0304473.s010.tif]

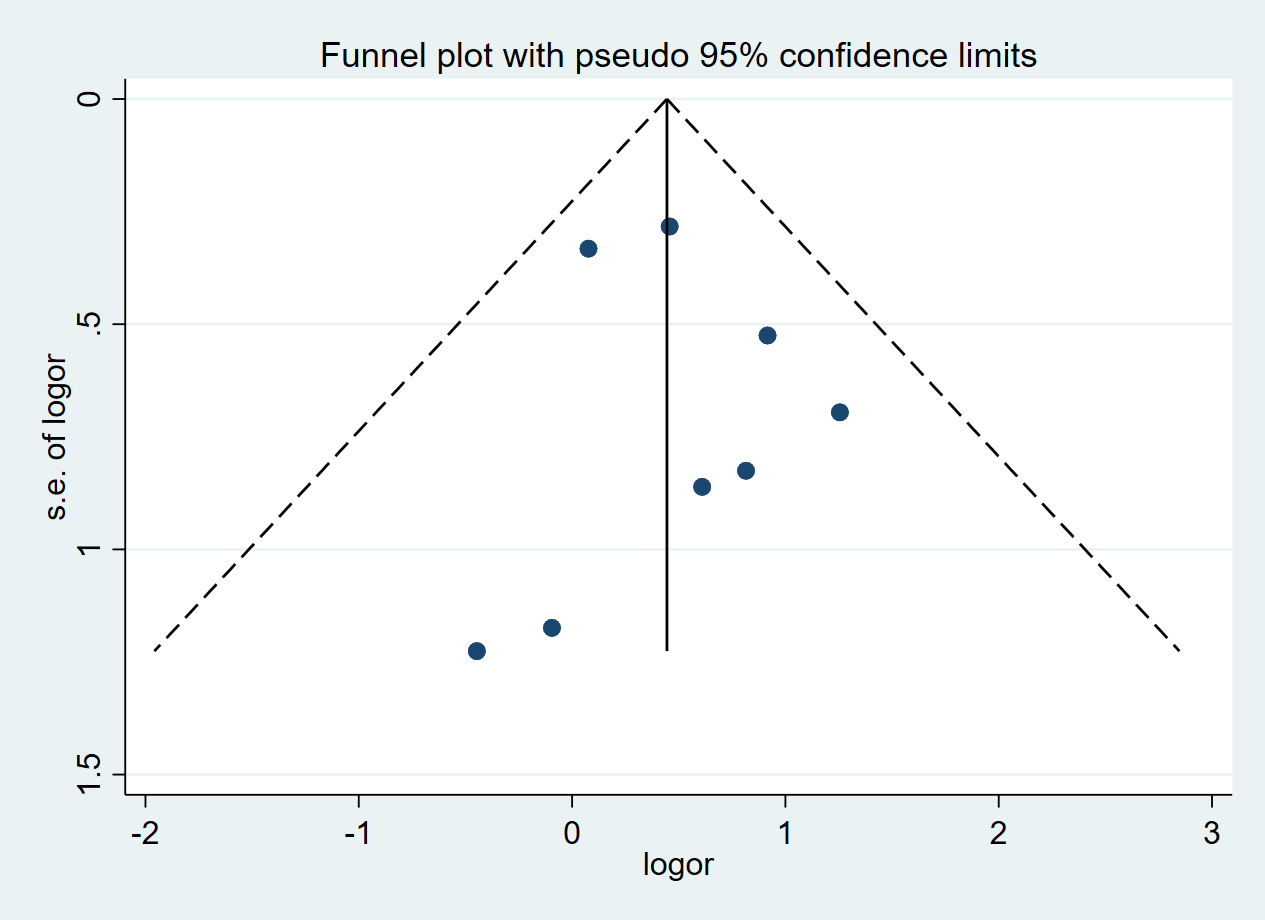

Supplement: S2 Fig — (TIF) [file pone.0304473.s011.tif]

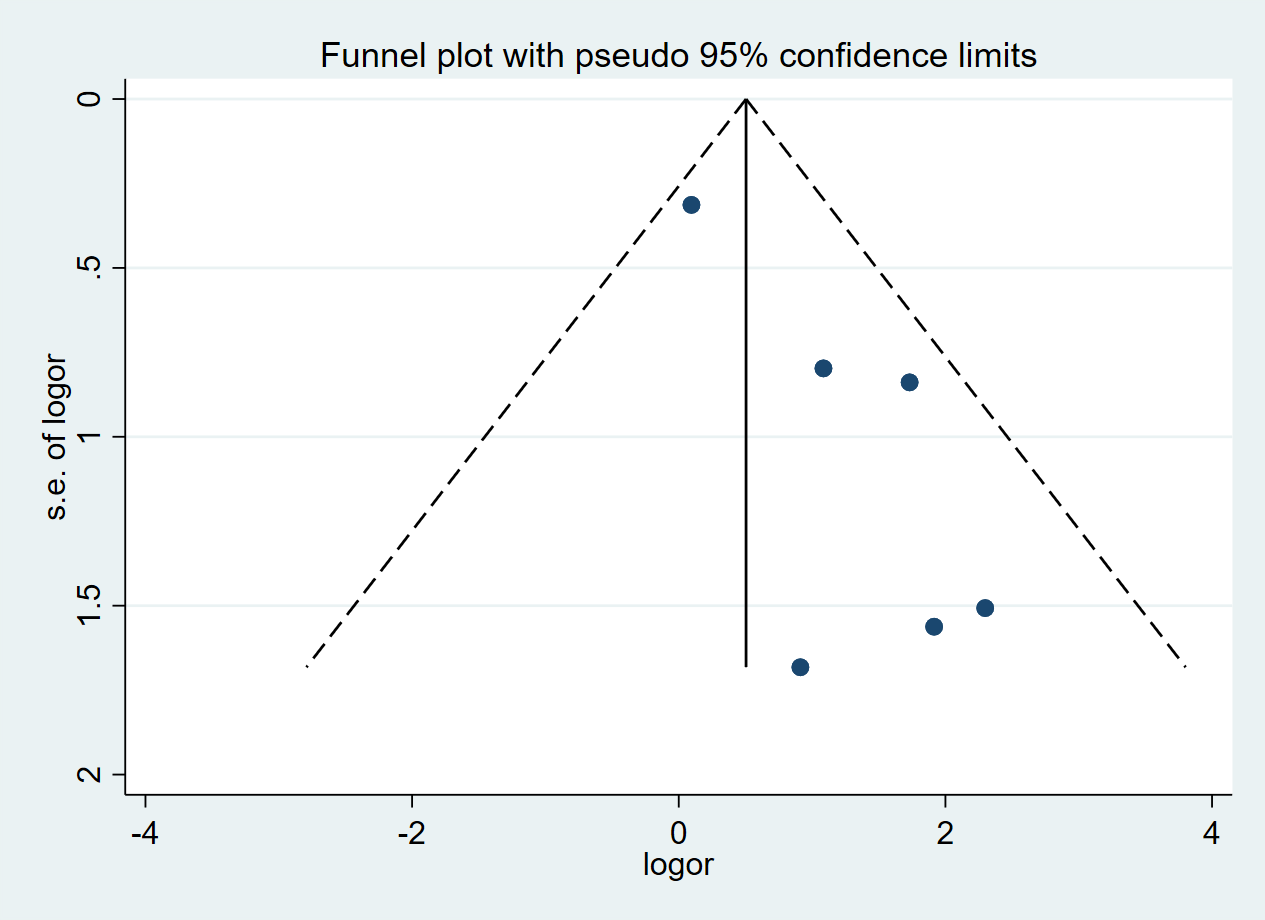

Supplement: S3 Fig — (TIF) [file pone.0304473.s012.tif]

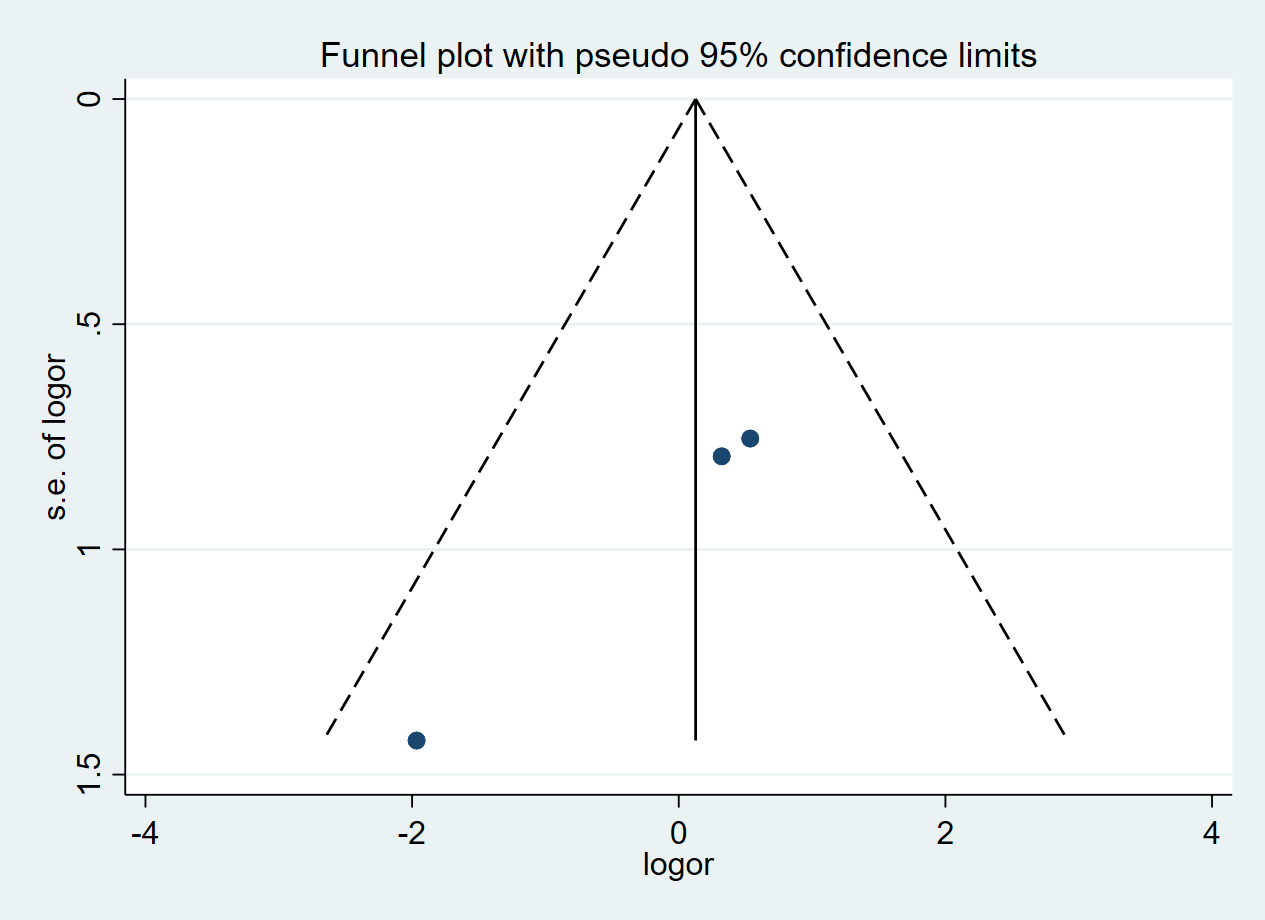

Supplement: S4 Fig — (TIF) [file pone.0304473.s013.tif]

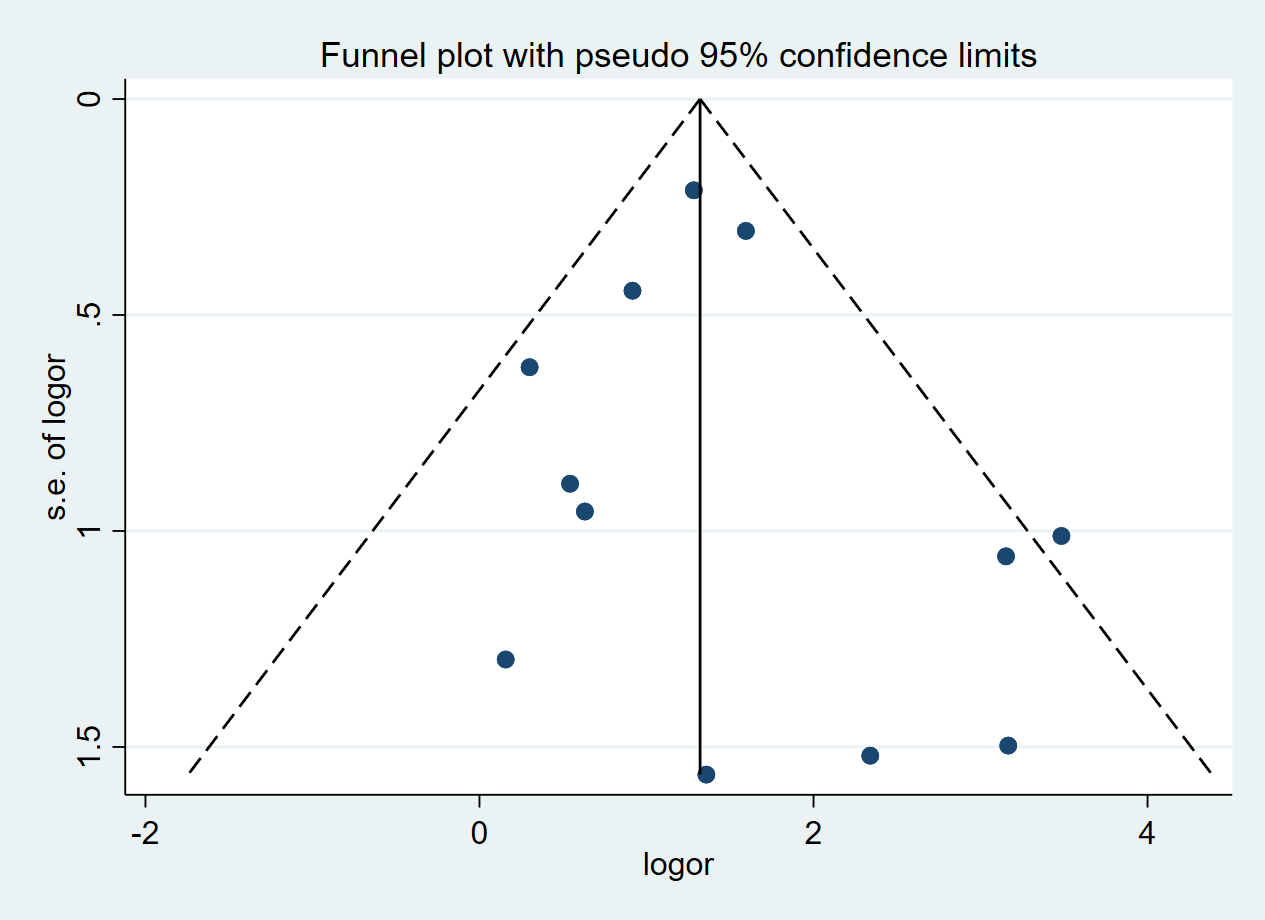

Supplement: S5 Fig — (TIF) [file pone.0304473.s014.tif]

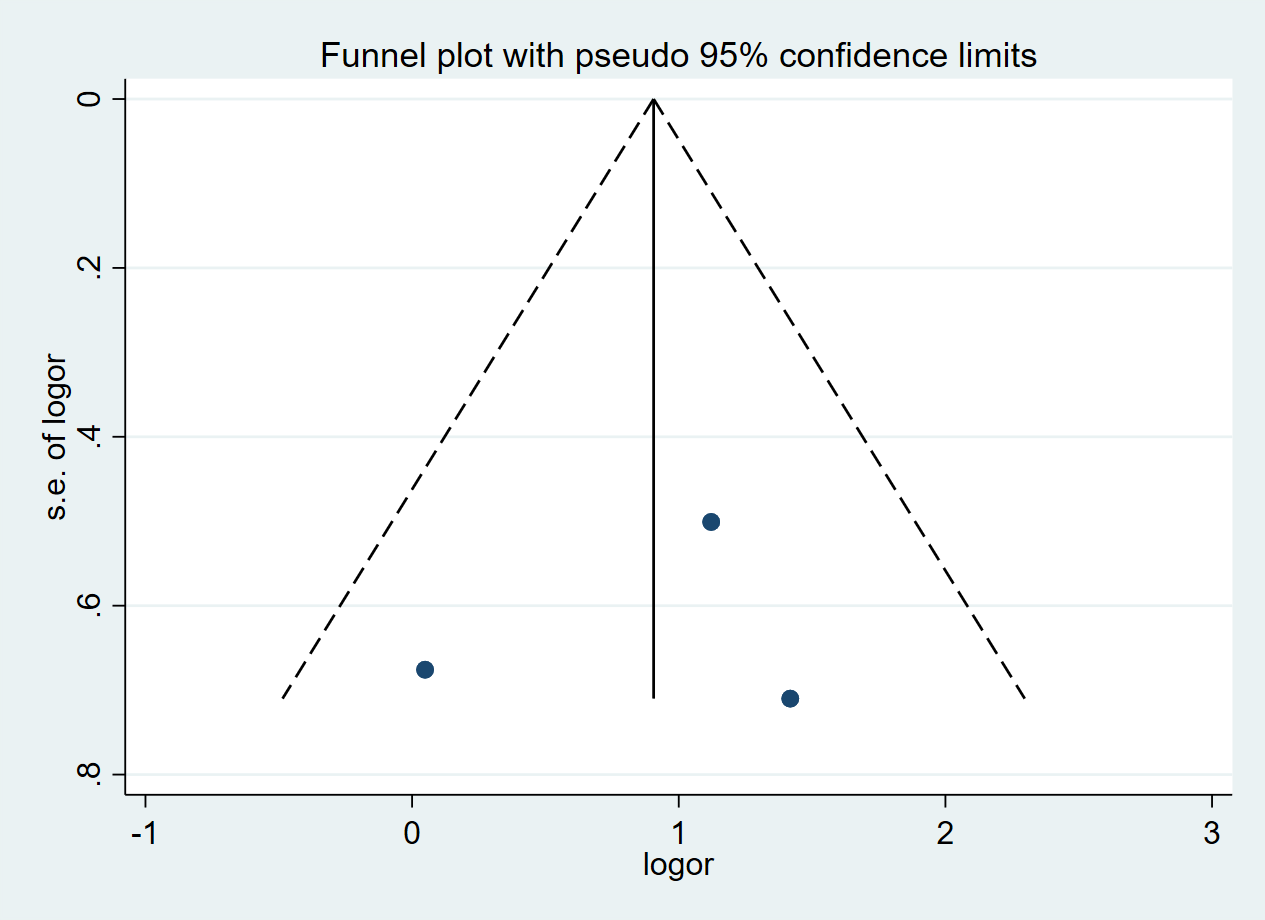

Supplement: S6 Fig — (TIF) [file pone.0304473.s015.tif]

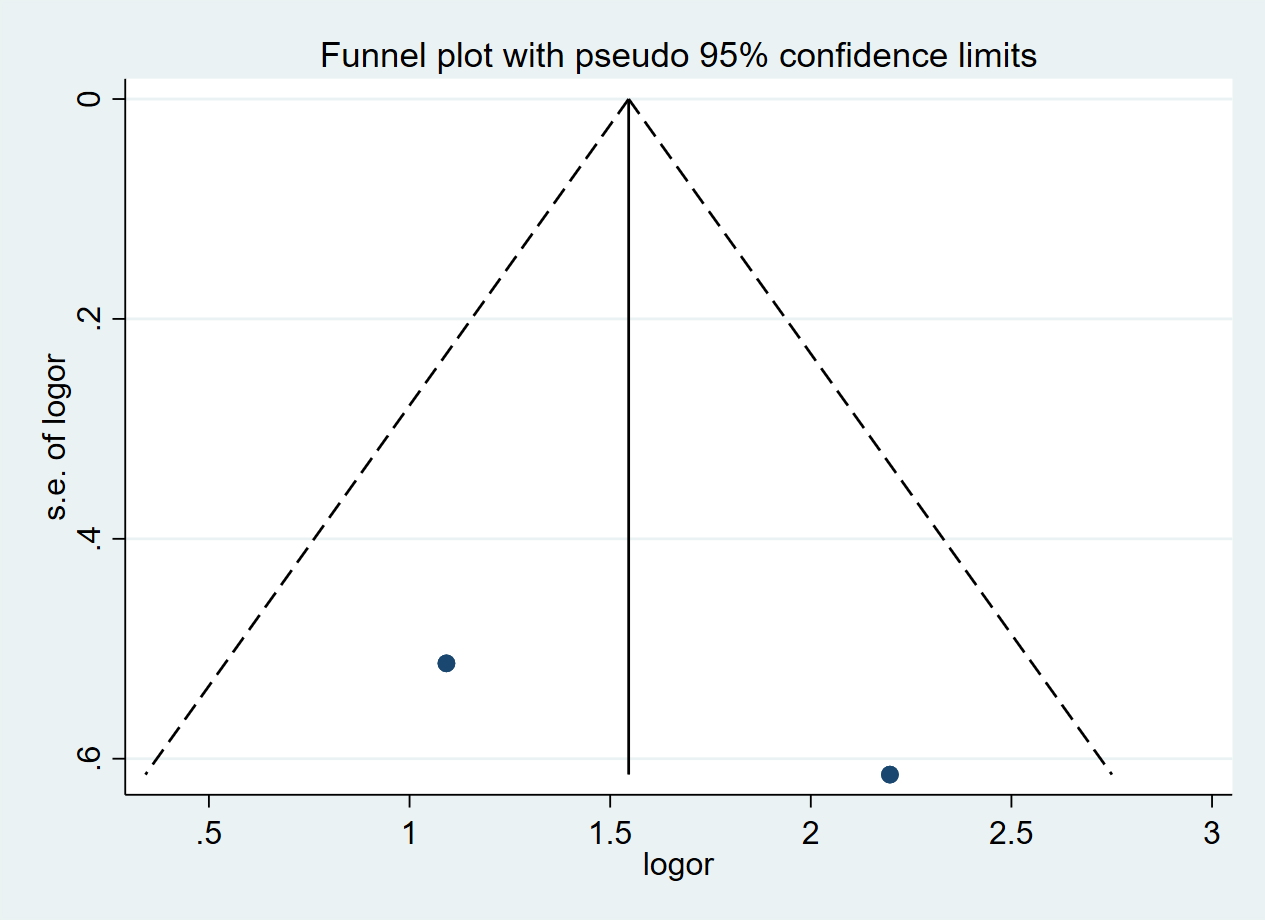

Supplement: S7 Fig — (TIF) [file pone.0304473.s016.tif]

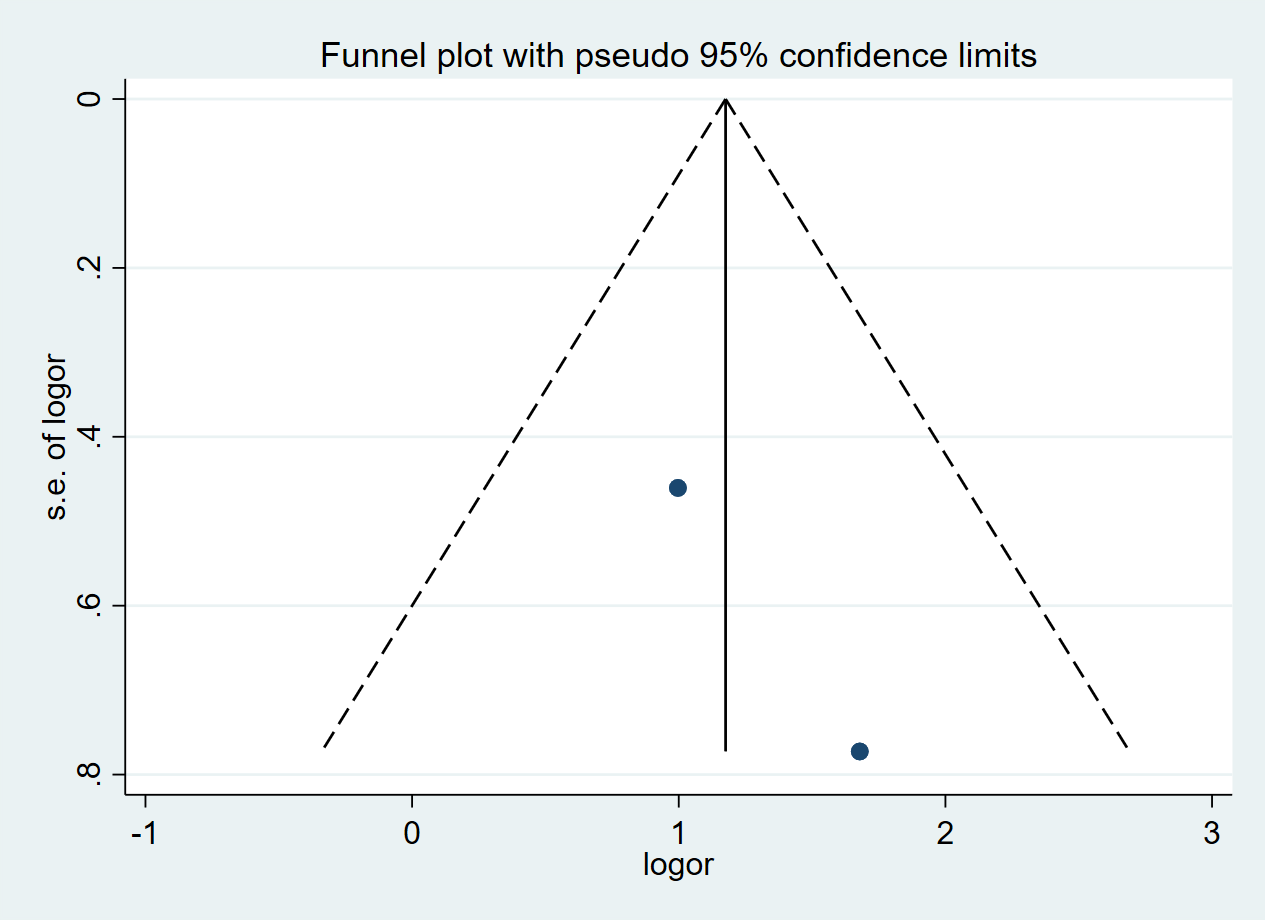

Supplement: S8 Fig — (TIF) [file pone.0304473.s017.tif]

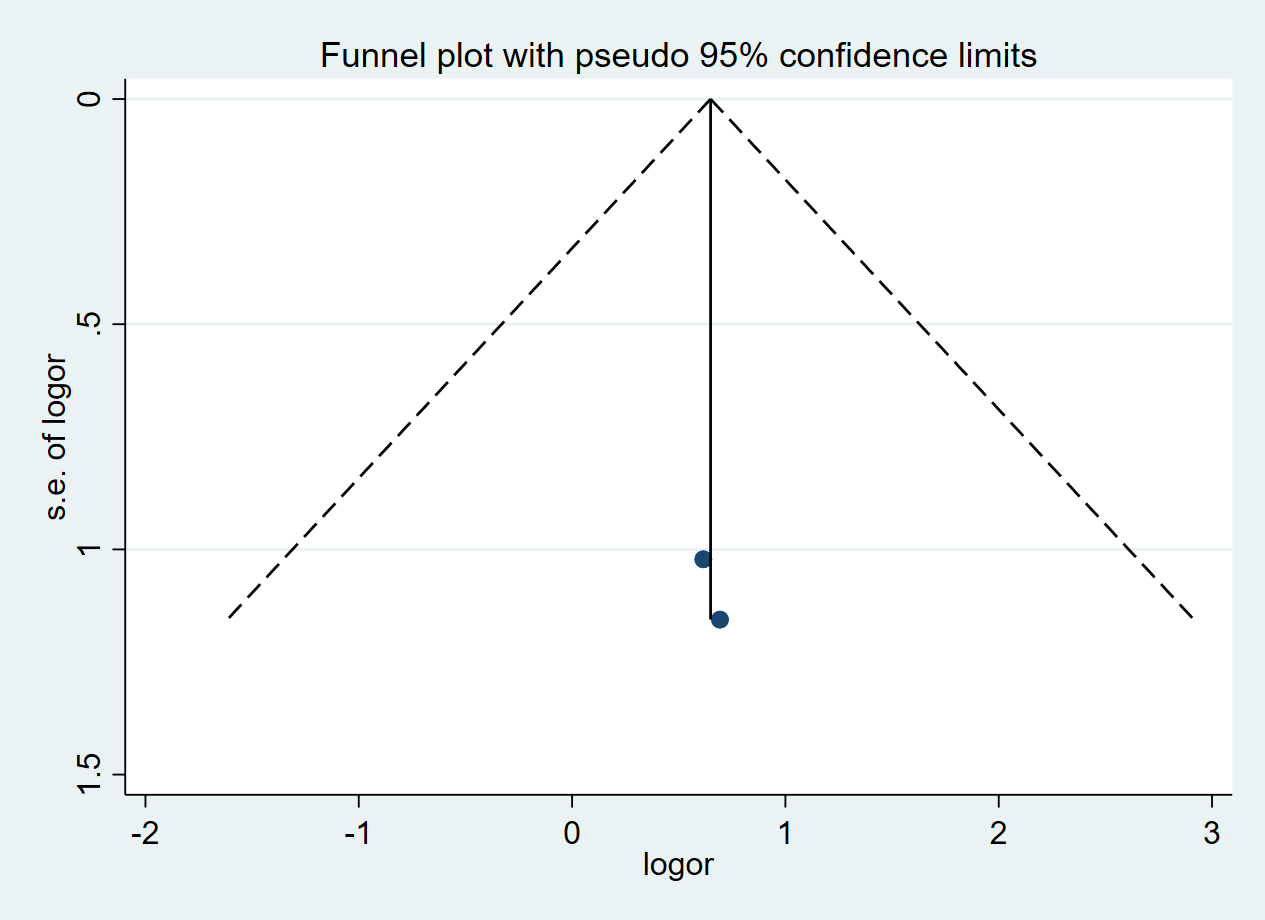

Supplement: S9 Fig — (TIF) [file pone.0304473.s018.tif]

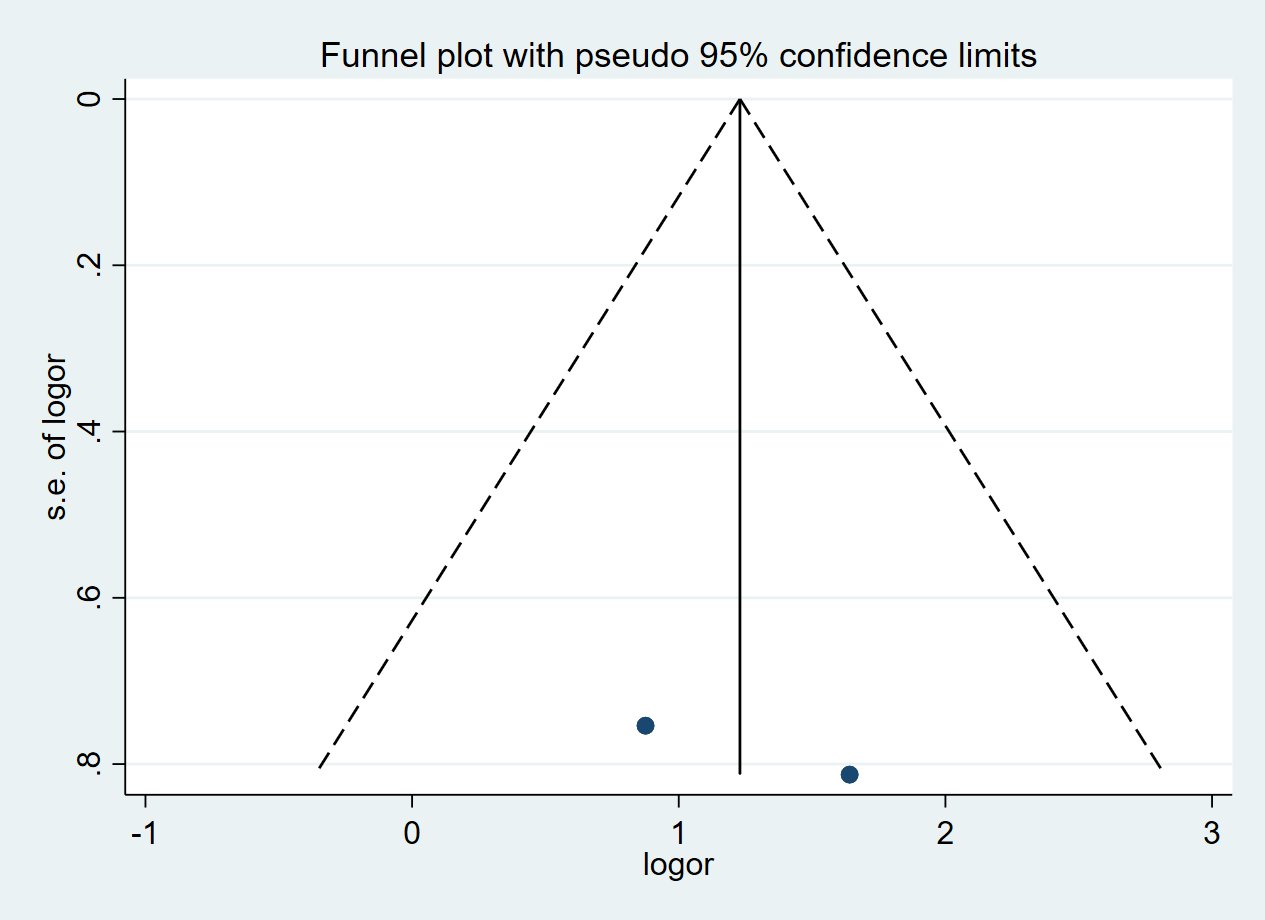

Supplement: S10 Fig — (TIF) [file pone.0304473.s019.tif]
