# Supplementary material for: Risk factors affecting spinal fusion: A meta-analysis of 39 cohort studies
Source: PLoS One. 2024 Jun 7;19(6):e0304473. doi: 10.1371/journal.pone.0304473 (PMC11161075; doi:10.1371/journal.pone.0304473)
Supplement: Attachment — Submitted filename: Response to Reviewers.docx [file pone.0304473.s020.docx]

**Detailed Rebuttal Letter to Editor and Reviewer**

Dear Prof. Hassan Zmerly,

Academic Editor, PLOS ONE

Thank you for forwarding the Reviewer comments to us. We have carefully gone through their feedback and have made the necessary changes to our manuscript. We appreciate the insightful feedback provided by the reviewers, as they have helped us improve the quality of our research. Please find below a detailed list of the changes made in response to the Editor and each of the Reviewer's concerns:

**Editor’s comments:**

1) A rebuttal letter that responds to each point raised by the academic editor and reviewer(s). You should upload this letter as a separate file labeled 'Response to Reviewers'.

Response: We respond to each point of view put forward by academic editors and reviewers, and if necessary, we modify the content of the manuscript according to the suggestions of academic editors and reviewers (the changes are marked in red font). Finally, we will upload this letter as a separate file labeled 'Response to Reviewers'.

2) A marked-up copy of your manuscript that highlights changes made to the original version. You should upload this as a separate file labeled 'Revised Manuscript with Track Changes'.

Response: All the revised contents in the manuscript are highlighted in red font. And we will upload this as a separate file labeled 'Revised Manuscript with Track Changes'.

3) An unmarked version of your revised paper without tracked changes. You should upload this as a separate file labeled 'Manuscript'.

Response: We separately uploaded a manuscript file without any markup, which was marked as "manuscript".

4) If you would like to make changes to your financial disclosure, please include your updated statement in your cover letter. Guidelines for resubmitting your figure files are available below the reviewer comments at the end of this letter.

Response: Thank editor for reminding us that we do not need to change our financial disclosure.

**Journal Requirements:**

1)Please ensure that your manuscript meets PLOS ONE's style requirements, including those for file naming. The PLOS ONE style templates can be found at

https://journals.plos.org/plosone/s/file?id=wjVg/PLOSOne_formatting_sample_main_body.pdf and

https://journals.plos.org/plosone/s/file?id=ba62/PLOSOne_formatting_sample_title_authors_affiliations.pdf

Response: We re-referenced the specified PLOS One style template to adjust the content format of our manuscript to ensure that our manuscript meets the style requirements of PLOS One.

2) We noticed you have some minor occurrence of overlapping text with the following previous publication(s), which needs to be addressed:

-https://link.springer.com/article/10.1007/s10143-023-020410?

https://journals.lww.com/international-journal-of-

surgery/fulltext/2023/10000/risk_factors_of_epidural_hematoma_in_patients.30.aspx

In your revision ensure you cite all your sources (including your own works), and quote or rephrase any duplicated text outside the methods section. Further consideration is dependent on these concerns being addressed.

Response: All relevant data for our articles are provided in the manuscript and supporting information files.

3) Thank you for stating the following in the Funding Section of your manuscript:

“This work was supported by the Health and Family Planning Commission Program of Hunan Province (no. 202204074707), the Health and Family Planning Commission Program of Wuhan City (no. WX18C29), and Natural Science Foundation of Hunan Province (no. 2022JJ30516).”

We note that you have provided funding information that is not currently declared in your Funding Statement. However, funding information should not appear in the Acknowledgments section or other areas of your manuscript. We will only publish funding information present in the Funding Statement section of the online submission form.

Please remove any funding-related text from the manuscript and let us know how you would like to update your Funding Statement. Currently, your Funding Statement reads as follows:

“The author(s) received no specific funding for this work.”

Please include your amended statements within your cover letter; we will change the online submission form on your behalf.

Response: We re-examined the Funding section of the manuscript and deleted the description of the funding statement in the manuscript.

Accordingly, we have revised the funding section as follows:

The authors received no specific funding for this work.

4) Before we proceed with your manuscript, please also provide non-author contact information (phone/email/hyperlink) for a data access committee, ethics committee, or other institutional body to which data requests may be sent. If no institutional body is available to respond to requests for your minimal data, please consider if there any institutional representatives who did not collaborate in the study, and are not listed as authors on the manuscript, who would be able to hold the data and respond to external

requests for data access? If so, please provide their contact information (i.e., email address). Please also provide details on how you will ensure persistent or long-term data storage and availability.

Response: Uploaded as supplementary information.

5) Please ensure that you include a title page within your main document. You should list all authors and all affiliations as per our author instructions and clearly indicate the corresponding author.

Response: We list all authors and all affiliations as per our author instructions and clearly indicate the corresponding author in the title page.

6) Please include your tables as part of your main manuscript and remove the individual files. Please note that (should remain/ be uploaded) as separate "supporting information" files.

Response: We have placed the table in the appropriate place in the article and deleted the separate files. In addition, we also upload "supporting information" as a separate file.

7）Please include captions for your Supporting Information files at the end of your manuscript, and update any in-text citations to match accordingly. Please see our Supporting Information guidelines for more information: <http://journals.plos.org/plosone/s/supporting-information>.

Response: We added the title information of the supporting material at the end of the manuscript and updated the reference in the manuscript.

**Reviewer #1:**

**Comments to the Author**

Thank you for submitting your work to PLOS ONE. The study aims to explore the potential risk factors regarding nonunion after spinal fusion surgery, leveraging a comprehensive meta-analysis approach. Before considering this manuscript for publication, I recommend a major revision based on the following aspects.

1) Surgical variability and fusion rate analysis should be added. Your study predominantly focused on general risk factors affecting spinal fusion without considering the influence of surgical variability, such as the type of surgery or the site of surgery. Given that these variables can significantly impact fusion outcomes, it would enhance the manuscript's value to report on how different types of surgeries or fusion sites correlate with fusion rates. It offers a more nuanced understanding of risk factors.

Response: The reviewers pointed out that we should increase the analysis of the influence of surgical variability (including surgical site and type of operation) on the fusion rate. We are very grateful to the reviewers for this important suggestion, and we very much agree with the reviewers' suggestion. Therefore, we re-extract the data from the included study, and explore the effects of different surgical sites and types of surgery on the fusion rate through stratified analysis, which will enrich our research content and results.

**S5 Table.** Fusion Rate by Study-level Factors.

| Variable | Rate, % | 95% CI | Degree of heterogeneity  (I² statistics; %) | *P* value | No. of included Studies | *P* for interaction |
| --- | --- | --- | --- | --- | --- | --- |
| **Total** | **89.2** | **87.4-91.1** | **86.9** | **<0.001** | **39** | **<0.001** |
| Study design |  |  |  |  |  | <0.001 |
| Retrospective | 92.0 | 90.2-93.8 | 81.2 | <0.001 | 23 |  |
| Prospective | 84.2 | 79.7-88.6 | 88.9 | <0.001 | 16 |  |
| Regions |  |  |  |  |  | <0.001 |
| China | 89.6 | 86.0-93.3 | 71.0 | 0.001 | 9 |  |
| USA | 89.3 | 86.4-92.1 | 91.1 | <0.001 | 19 |  |
| others | 88.6 | 85.1-92.2 | 82.3 | <0.001 | 11 |  |
| Sample size |  |  |  |  |  | <0.001 |
| <100 | 89.7 | 87.0-92.4 | 75.1 | <0.001 | 21 |  |
| 100-500 | 90.1 | 89.1-91.1 | 93.7 | <0.001 | 14 |  |
| >500 | 92.1 | 91.2-93.0 | 0 | 0.593 | 4 |  |
| Study year |  |  |  |  |  | <0.001 |
| <2015 | 89.3 | 86.3-92.2 | 86.2 | <0.001 | 19 |  |
| 2015-2020 | 87.0 | 82.8-91.2 | 84.1 | <0.001 | 10 |  |
| >2020 | 91.4 | 88.4-94.5 | 81.9 | <0.001 | 10 |  |
| Mean follow-up period | | |  |  |  | <0.001 |
| <24 months | 88.7 | 85.7-91.0 | 86.1 | <0.001 | 18 |  |
| 24-48 months | 88.5 | 85.1-91.5 | 90.2 | <0.001 | 16 |  |
| >48 months | 92.6 | 91.4-93.9 | 0 | 0.509 | 5 |  |
| Female % |  |  |  |  |  | <0.001 |
| <50 | 85.1 | 79.4-90.8 | 91.7 | <0.001 | 13 |  |
| ≥50 | 91.1 | 89.2-93.1 | 80.6 | <0.001 | 22 |  |
| NR | 91.6 | 89.4-91.9 | 37.3 | 0.188 | 4 |  |
| Surgical sites |  |  |  |  |  | <0.001 |
| Lumbar | 87.8 | 85.2-90.5 | 89.9 | <0.001 | 25 |  |
| Cervical | 92.0 | 89.7-94.3 | 73.7 | <0.001 | 13 |  |
| Surgical types |  |  |  |  |  | <0.001 |
| TLIF or PLIF | 85.5 | 81.2-89.7 | 92.4 | <0.001 | 12 |  |
| LLIF | 95.3 | 90.8-99.7 | 70.2 | 0.035 | 3 |  |
| ALIF | 86.3 | 81.1-91.5 | 42.6 | 0.175 | 3 |  |
| ACDF | 92.3 | 90.1-94.5 | 71.3 | <0.001 | 11 |  |
| Others | 88.2 | 83.5-92.9 | 85.4 | <0.001 | 10 |  |

Abbreviations: ACDF, Anterior cervical discectomy and fusion; ALIF, Anterior lumbar interbody fusion; CI, confidence interval; LLIF, Lateral lumbar interbody fusion; NR, not reported; PLIF, posterior lumbar interbody fusion; TLIF, transforaminal lumbar interbody fusion.

Accordingly, we have revised the result section as follows:

In addition, in the stratified analysis of surgical sites and surgical methods, we found that the fusion rate of cervical surgery was 92% (95% CI, 89.7% to 94.3%, I^2^=73.7%, P < 0.001), which was Higher than the fusion rate of lumbar surgery. And in lumbar fusion surgery, the fusion rate of lateral approach was significantly higher than that of other approaches (Rate, 95.3%; 95% CI, 90.8% to 99.7%, I^2^=70.2%, P < 0.001).

(Page 16, Paragraph 1)

2) Inclusion of forest plots in the main manuscript, not in supplement materials. While it is noted that forest plots were provided as supplementary figures, integrating these plots into the main figures of the manuscript would likely improve the accessibility and interpretability of your results for readers.

Response: We are very grateful to the reviewers for their important suggestions for our study. The reviewer pointed out that we should put the forest plots in the manuscript to improve the reader's accessibility and interpretability of the results. We very much agree with the reviewer's point of view. In order to respond to the important suggestion of the reviewer, we included all the forest plots in the main manuscript. At the same time, the title of the picture is added where it is quoted in the manuscript.

3) I also recommend adding tables that show baseline characteristics of the references, such as country, type and site of surgery, sample size, mean follow-up period, as well as included variables and significant variables for each reference.

Response: The reviewers suggested that show baseline characteristics of the references, such as country, type and site of surgery, sample size, mean follow-up period, as well as included variables and significant variables for each reference. We believe that this suggestion of the reviewers is of great importance and is of great help to improve the quality of this study. For this reason, we have added information about the inclusion of country, type and site of surgery and sample size and mean follow-up period in Table 1.

**Table 1. Characteristics of studies included in meta-analysis.**

| **First author** | **Year** | **Country** | **Observation Period** | **Study Design** | **Sample size** | **Female %** | **Mean age** | **Measurements of fusion** | **Mean Follow-up period** |
| --- | --- | --- | --- | --- | --- | --- | --- | --- | --- |
| Glassman, S. D. | 2000 | USA | 1992-1996 | Retrospective | 357 | 43.0 | 43.9 | 3D-CT | 24 months |
| Bose, B. | 2001 | USA | NR | Retrospective | 106 | 55.7 | 50.1 | X-ray | ≥12 months |
| Samartzis, D. | 2003 | USA | NR | Retrospective | 80 | 36.3 | 49.0 | Radio graphs | 16 months |
| Glassman, S. D. | 2003 | USA | NR | Retrospective | 137 | 60.5 | 60.3 | CT | 12 months |
| Gerszten, P. C. | 2011 | USA | 2005-2007 | Retrospective | 99 | 42.8 | 42.8 | MRI and CT | 24 months |
| Hoffmann, M. F. | 2012 | USA | 2003-2009 | Retrospective | 1398 | 58.9 | 60.0 | CT and X-ray and MRI | 183 months |
| Luszczyk, M. | 2013 | USA | NR | Retrospective | 573 | NR | NR | Radio graphs | 24 months |
| Urrutia, J. | 2013 | Chile | 2004-2010 | Retrospective | 47 | 76.6 | 46.5 | CT | 50.1 months |
| Frenkel, M. B. | 2013 | USA | 1997-2012 | Retrospective | 45 | 57.7 | 50.0 | X-ray and CT | 161 months |
| Adams, C. L. | 2014 | Australia | 2007-2010 | Retrospective | 70 | 50.0 | 55.4 | Radio graphical | 12 months |
| Tan, B. | 2015 | China | 2007-2010 | Retrospective | 146 | 41.1 | 64.7 | Radio graphs | 24 months |
| Yang, Y. | 2016 | China | 2011-2014 | Retrospective | 132 | 32.6 | 44.2 | CT | 24 months |
| Zhang, Y. H. | 2017 | China | 2007-2015 | Retrospective | 32 | NR | 6.80 | CT | 45 months |
| Phan, K. | 2018 | Australia | NR | Retrospective | 137 | 52.6 | 56.7 | CT | 12 months |
| Weng, F. | 2018 | China | 2015-2017 | Retrospective | 80 | 71.3 | 65.6 | NR | 6 months |
| Nourian, A. A. | 2019 | USA | NR | Retrospective | 93 | 67.0 | 65.0 | CT | >12 months |
| Niu, S. | 2020 | NR | 2007-2017 | Retrospective | 927 | 59.0 | 63.8 | CT | 6 months |
| Wu, F. L. | 2020 | China | NR | Retrospective | 50 | 48.0 | 61.2 | MRI and CT | 30 months |
| Son, H. J. | 2021 | USA | 2009-2019 | Retrospective | 121 | 76.0 | 68.2 | CT | >12 months |
| Tan, Y. | 2021 | Japan | 2017-2020 | Retrospective | 98 | 64.3 | 69.5 | CT | 33.5 months |
| Tannoury, C. | 2021 | USA | 2008-2019 | Retrospective | 220 | 62.8 | 66.0 | CT | 12 months |
| Wang, H. | 2021 | China | NR | Retrospective | 153 | 66.8 | 50.1 | CT | 40.8 months |
| Li, Z. | 2022 | China | 2017-2019 | Retrospective | 77 | 52.0 | 44.6 | CT | 12 months |
| Bishop, R. C. | 1996 | USA | 1991-1995 | Prospective | 132 | 54.6 | 45.2 | X-ray | 31 months |
| Tuli, S. K. | 2004 | USA | 1995-1999 | Prospective | 57 | 47.4 | 52.2 | Radio graphs | 6 months |
| Suchomel, P. | 2004 | Czech | 1998-2000 | Prospective | 79 | 37.9 | 47.8 | X-rays | 48 months |
| Burkus, J. K. | 2004 | USA | NR | Prospective | 46 | NR | NR | Radio assessments | 24 months |
| Cammisa Jr, F. P. | 2004 | USA | NR | Prospective | 120 | 49.0 | 48.0 | X-ray | 24.5 months |
| Burkus, J. K. | 2005 | USA | 1998-2001 | Prospective | 131 | 61.1 | 41.5 | Radio graphs and CT | ≥24 months |
| Joseph, V. | 2007 | Canada | 2003-2005 | Prospective | 33 | 39.4 | 49.7 | CT | 25 months |
| Frantzén, J. | 2011 | Finland | 1996-1998 | Prospective | 17 | 70.5 | 49.4 | CT and X- ray and MRI | 132 months |
| Wu, Z. X. | 2012 | China | 2004-2009 | Prospective | 157 | 61.2 | 62.1 | Radio graphs | 43 months |
| Ravindra, V. M. | 2015 | USA | 2011-2012 | Prospective | 133 | 44.0 | 57.0 | CT | >12 months |
| Burkus, J. K. | 2017 | USA | NR | Prospective | 710 | NR | NR | X-ray | 24 months |
| Moazzeni, K. | 2018 | Iran | 2014-2015 | Prospective | 96 | 62.5 | 57.8 | CT and X-ray | 12 months |
| Ravindra, V. M. | 2019 | USA | 2011-2012 | Prospective | 58 | 41.4 | 57.1 | X-ray | ≥12 months |
| Srour, R. | 2020 | France | 2017-2018 | Prospective | 53 | 50.9 | 65.0 | CT | 12 months |
| Hyun, S. J. | 2021 | Korea | NR | Prospective | 76 | 44.7 | 63.4 | X-ray and CT | 12 months |
| Zhang, W. | 2022 | China | 2018-2020 | Prospective | 69 | 61.5 | 54.6 | CT | 6 months |

**Table 1. Characteristics of studies included in meta-analysis (Continued).**

| **First author** | **Year** | **Fusion definition** | **significant variables** | **Fusion rate (%)** | **Surgical sites** | **Surgical types** |
| --- | --- | --- | --- | --- | --- | --- |
| Glassman, S. D. | 2000 | NR | Smoking | Smokers: 79.3%, Nonsmokers: 85.8% | Lumbar | Posterior fusion |
| Bose, B. | 2001 | Trabecular bony bridging across the disk space and lack of motion on flexion extension. | Smoking | Nonsmoking: 96.67%, smoking: 97.83% | Cervical | ACDF |
| Samartzis, D. | 2003 | A bony bridge incorporated the graft and the adjacent end plates and no radiolucencies or motion. | Autograft, allograft | Allograft: 94.3%; autograft: 100% | Cervical | ACDF |
| Glassman, S. D. | 2003 | NR | IDDM, NIDDM | NIDDM: 78.26%/IDDM: 74.28%, Control: 94.59% | Lumbar | PLIF |
| Gerszten, P. C. | 2011 | Bridging bone that traversed from end plate to end plate, with no motion detected on flexion extension lateral radio graphs. | rhBMP-2 | With rhBMP-2: 95.5%, Without rhBMP-2: 92.5% | Lumbar | Interbody fusions in the lumbosacral spine |
| Hoffmann, M. F. | 2012 | NR | DBM with rhBMP-2 | rhBMP-2: 95.7%/DBM: 86.9, Autograft: 84.8% | Lumbar | PLIF, TLIF |
| Luszczyk, M. | 2013 | Bony trabecular bridging between the graft and vertebral body, and motion was absent. | Smoking | Smoking: 91%, control: 91.6% | Cervical | ACDF |
| Urrutia, J. | 2013 | Trabeculae crossing the graft-vertebral body interface on both sides of the graft either. | Smoke | Smoke: 91.7% | Lumbar | Circumferential lumbar spinal fusion |
| Frenkel, M. B. | 2013 | The presence of motion on flexion-extension radio graphs. | rhBMP-2 | With rhBMP-2: 100%, Without rhBMP-2: 83% | Cervical | Anterior cervical fusion |
| Adams, C. L. | 2014 | NR | rhBMP-2, LBC | rhBMP-2: 94.1%, LBC: 89.5% | Lumbar | PLIF or TLIF |
| Tan, B. | 2015 | NR | rhBMP-2, ICBG | rhBMP-2: 87.7%, ICBG: 74% | Cervical | ACDF |
| Yang, Y. | 2016 | Evidence of continuous bridging bone between the adjacent end plates of the involved motion segment, radiolucent lines at 50% or less of the graft vertebra interfaces. | Two-level or single level ACDF | Single level: 94.6%, Two-level: 92.7% | Cervical | ACDF |
| Zhang, Y. H. | 2017 | The lack of hardware failure and presence of continuous bridging trabecular bone between the dorsal elements of the C1 and C2 on CT scans. | Structural allograft or autograft | Allograft: 94%, autograft: 100% | Cervical | Atlantoaxial fusion |
| Phan, K. | 2018 | Bridging trabecular formation across the intervertebral disk space with the absence of radiolucency spanning more than half of the implant. | Smoking | Smoking: 69.6%, no smoking: 85.1% | Lumbar | ALIF |
| Weng, F. | 2018 | Complete fusion and remodeling after intervention with newly-formed trabeculae; complete bone block after intervention. | EPS, CPS | EPS: 90%, CPS: 50% | Lumbar | Lumbar short-segment fixation and fusion |
| Nourian, A. A. | 2019 | NR | LLIF with rhBMP-2 | 1-level LLIF with rhBMP-2: 92%, 2-level LLIF with rhBMP-2: 86% | Lumbar | LLIF |
| Niu, S. | 2020 | NR | RhBMP-2 | RhBMP-2: 92.5%, no rhBMP-2: 71.4% | Lumbar | TLIF |
| Wu, F. L. | 2020 | NR | MIDLF, MI-TLIF | MIDLF: 94%, MI-TLIF: 88% | Lumbar | MIDLF, MI-TLIF |
| Son, H. J. | 2021 | Lenke grade B and BSF grade-3. | ICBG, E.BMP-2 | ICBG: 97.1%, E.BMP-2: 100% | Lumbar | LIF and PLF |
| Tan, Y. | 2021 | NR | Simultaneous single-position O-arm-navigated OLIF and PPS, MI-PLIF/TLIF | OLIF and PPS: 96.8%, MI-PLIF/TLIF: 94.2% | Lumbar | MIS-ATP-lumbar fusions |
| Tannoury, C. | 2021 | Examining consecutive sagittal and coronal cuts for continuous bony bridges. | Smoke, L5-S1 | Smoke: 95.3%, L5-S1: 95.2% | Lumbar | OLIF and PPS, MI-PLIF/TLIF |
| Wang, H. | 2021 | Mental ROM less than 3° in X-ray and continuous bone bridge demonstrated in CT imaging. | Male, smoke | Smoke: 87.84% | Cervical | CDR and ACDF |
| Li, Z. | 2022 | Unilateral or bilateral grade I or II fusion. | Modified facet joint fusion or posterolateral fusion | MFF: 94.3%, PLF: 76.2% | Lumbar | Modified facet joint fusion or posterolateral fusion |
| Bishop, R. C. | 1996 | Bony trabeculae were seen crossing the involved interspace. | Autograft tricortical iliac and the allograft tricortical iliac | Single-level ACDF: (autograft: 97%, allograft: 87%), multiple level interbody fusion: (autograft: 100%, allograft: 89%) | Cervical | Single-level or multiple-Level ACDF |
| Tuli, S. K. | 2004 | Presence of any trabeculae bridging between the vertebral body and allograft at the upper and lower aspects. | One level or two-level corpectomy | Cephalad aspect of the graft-host interface: 92%, caudad aspect: 93%, one-level corpectomy: 86%, two-level corpectomy: 100% | Cervical | Cervical decompressive corpectomy and reconstruction |
| Suchomel, P. | 2004 | Complete bridging of trabeculae between adjacent vertebral bodies and bone graft. | Number of fused levels, smoke, autologous, allogenic bone grafts | Autografts: 94.6%, allografts: 85.5%, smoke: 92.4% | Cervical | One- or two-level ACDF |
| Burkus, J. K. | 2004 | NR | rhBMP-2, ICBG | rhBMP-2-treated: 100%, auto graft-treated: 68.4% | Lumbar | ALIF |
| Cammisa Jr, F. P. | 2004 | NR | Grafton®, auto graft | Grafton® side: 52%, auto graft side: 54% | Lumbar | Posterolateral spine fusion |
| Burkus, J. K. | 2005 | (1) The presence of bridging trabecular bone connecting vertebral bodies through or around dowels, (2) no radiolucent area involving > 50% of the interface between dowels and end plates. | rhBMP-2/ACS, ICBG | rhBMP-2-treated: 98.5%, autograft-treated: 76.1% | Lumbar | Single-level ALIF |
| Joseph, V. | 2007 | The presence of bridging bone through the cage or external to it. | rhBMP-2 | With BMP: 94.4%, Without BMP: 89.4% | Lumbar | PLIF and TLIF |
| Frantzén, J. | 2011 | Bridging of bone between the transverse processes in addition to the incorporation of bone between the transverse processes. | BAG | BAG: 90%, Autologous Bone: 100% | Lumbar | Posterolateral spondylodesis |
| Wu, Z. X. | 2012 | Clear trabecular bone bridging across the segment to be fused, translation of 3 mm or less and angulation of < 5° on flexion-extension radio graphs. | EPS, CPS | EPS: 92.5%, CPS: 80.5% | Lumbar | Transpedicle fixation |
| Ravindra, V. M. | 2015 | The presence of bone trabeculation, without evidence of instrumentation loosening or breakage, and no observed motion between the graft and instrumentation. | Vitamin D | 84% | spine | Spinal fusion |
| Burkus, J. K. | 2017 | NR | rhBMP-2, ICBG | rhBMP-2-treated: 99.4%, autograft-treated: 87.2% | Cervical | Single-level anterior cervical arthrodesis |
| Moazzeni, K. | 2018 | Bridging bone remodeling across the transverse processes between the adjacent vertebrae. | Diabetic and non-diabetic patients after lumbar fusion | DM: 53%, control: 78% | Lumbar | Bilateral facet fusion |
| Ravindra, V. M. | 2019 | The presence of trabeculated bone, without evidence of hardware loosening or failure, and no observed motion between vertebral segments on X-rays. | Low vitamin D | Normal vitamin D: 76.47%, low vitamin D: 75.60% | Cervical | Anterior, posterior, or combined spinal fusion |
| Srour, R. | 2020 | Any sign of bony fusion inside or posterior to the device when viewing the postoperative CT scan. | Facet arthrodesis with or without PLIF | Facet arthrodesis with PLIF: 75.7%, non-PLIF: 88.7% | Lumbar | Facet osteosynthesis |
| Hyun, S. J. | 2021 | Less than 5 degrees of angular motion on flexion and extension radio graphs. | rhBMP-2 | DBM with rhBMP-2: 82.85%, DBM: 78.12% | Lumbar | TLIF |
| Zhang, W. | 2022 | Bridging bone bonding with both adjacent vertebral bodies. | Vitamin K2 + Vitamin D3 | Vitamin K2 + Vitamin D3: 91.18%, control: 71.43% | Lumbar | TLIF or PLIF |

Abbreviations: ACDF=Anterior cervical discectomy and fusion; ACS=absorbable collagen sponge; ALIF=Anterior lumbar interbody fusion; BAG=bioactive glass; BMI=body mass index; BSF=Brantigan, Steffee and Fraser; CDR= Cervical disc replacement; CPS=conventional pedicle screws; CT=computed tomography; DBM=demineralized bone matrix; DM=diabetes mellitus; E.BMP-2=E.coli-derived rhBMP-2; EPS=expandable pedicle screws; HA=hydroxyapatite; HO=heterotopic ossification; ICAG=iliac crest autograft; ICBG=iliac crest bone graft; IDDM=insulin-dependent diabetes mellitus; JOA=Japanese Orthopedic Association; LBC=local bone graft; LBP=low back pain; LLIF=Lateral lumbar interbody fusion; MFF=modified facet joint fusion; MIDLF=midline lumbar fusion; MI-PLIF/TLIF=Minimally invasive posterior or transforaminal lumbar interbody fusion; MIS-ATP=minimally invasive antepsoas; MI-TLIF=minimally invasive transforaminal lumbar interbody fusion; MRI=magnetic resonance imaging; NDI=Neck Disability Index; NIDDM=non-insulin dependent diabetes mellitus; NR=not reported; OLIF and PPS=Oblique lateral interbody fusion and percutaneous pedicle screw; OP-1=Osteogenic Protein-1; PLF=posterolateral fusion; PLIF=posterior lumbar interbody fusion; RCT=Randomized Controlled Trial; rhBMP-2=recombinant human bone morphogenetic protein-2; ROM=range of motion; RTC=rectangular titanium cage; TLIF=transforaminal lumbar interbody fusion; VAS=visual analog scale; W-TLIF=TLIF through Wiltse approach.

**Reviewer #2:**

**Comments to the Author**

1. In statistics and other analyses

1) There is a need to explain the data extraction process in meta-analysis in more detail.

Response: We sincerely thank the reviewers for the recognition of our study and work. In addition, the reviewers pointed out that we should explain the data extraction process in Meta analysis in more detail. We also think that this is very necessary. To this end, we have added and modified the content about the data extraction process in the methodology section of the manuscript.

Accordingly, we have revised the methods section as follows:

Two authors extracted data using a predesigned data extraction sheet. The specific extracted content was obtained by the relevant authors by reading the full text of the article and the contents of the table. When the data was incomplete or missing, we tried to contact the corresponding author of the article to obtain the relevant data. Differences between researchers in the process of extracting data were resolved through discussion or negotiation with third parties.

(Page 6, Paragraph 8)

2) The method for calculating the effect size of data extracted from each study must be described. Then, the authors must explain in detail how authors integrated these effect sizes and performed statistical analysis.

Response: The reviewer pointed out that we should add the method of calculating the effect amount and the description of how to carry on the statistical analysis to the effect amount in the manuscript. We very much agree with and thank the reviewers for this proposal. In this regard, our data processing method is described in more detail in the statistical analysis part of the method.

Accordingly, we have revised the methods section as follows:

The odds in each group were computed as *p/(1-p)* where *p* represents the proportion with exposure. The odds ratio (OR) was determined by dividing the odds in the fusion failure group by the odds in the comparator group. In the meta-analyses, study-specific log odds ratios were utilized as the outcome, and the aggregated estimates were then transformed into OR. If the OR > 1, it indicates a higher probability of fusion failure in the exposed group as opposed to the non-exposed group.

(Page 8, Paragraph 1)

2.From the results,

1) The authors mentioned MIS and the number of fused levels, and it seems that this should be described as a factor to be analyzed in the method. All factors to be analyzed must be introduced in the method, and the analysis details must be reported in the results.

Response: We are very grateful to the reviewers for their careful review of our research. Because of our mistake, we omitted the introduction of MIS and the number of fused levels in our method. For this reason, we revised the manuscript appropriately and re-added the content related to MIS and the number of fused levels.

Accordingly, we have revised the methods section as follows:

All of our analyses were performed using Stata software (Stata version 16.0, College Station, Texas, USA). We analyzed the risk factors affecting spinal fusion, including patient-related factors (e.g., smoking, diabetes, and vitamin D deficiency). surgery-related risk factors (e.g., allograft, without the use of BMP-2, conventional pedicle screw (CPS) fixation, and posterolateral fusion, MIS, number of fused levels)

(Page 9, Paragraph 1)

2) The authors found that factors that inhibit spinal fusion, such as smoking, diabetes, vitamin D deficiency, graft type, pedicle screw type, and fusion column, were extracted from 39 studies, but the number of studies was small, with only more than 500 subjects or an I^2^ value of more than 0.5. Is this why it is classified as high or medium quality? I think we need to present objective standards for this.

Response: The reviewers questioned the evidence level classification basis of our manuscript on risk factors. First of all, I would like to thank the reviewers for their important suggestions on the basis of the classification of the level of evidence. It has to be admitted that there is no uniform standard on the basis of the classification of the level of evidence. Previously, we have referred to a variety of criteria for the classification of the level of evidence. In the end, the standard currently used is selected. Because this set of standards is most applicable to this study. In addition, the standard for the classification of the level of evidence is now used frequently, and the articles expressed in a large number of high-scoring journals are also the criteria for the classification of this set of levels of evidence.

Grading Evidence Based on Egger’s *P* value, Sample Size and Heterogeneity.

| **Evidence** | **Definition** |
| --- | --- |
| Class 1 (High-quality) evidence | Defined as three conditions of Egger’s P value of the random effects model >0.1, a total population >1000, and lower between-study heterogeneity I2<50% are met simultaneously. |
| Class II (Moderate-quality) evidence | Defined as two of the three conditions of Egger’s P value of the random effects model >0.1, a pooled population >1000, and higher between-study heterogeneity I2 ≥50% are met. |
| Class III (Moderate-quality) evidence | Defined as one of the three conditions of Egger’s P value of the random effects model >0.1, a pooled population <1000, and lower between-study heterogeneity I2<50% are met. |
| Class IV (Low-quality) evidence | Defined as none of the three conditions of Egger’s P value of the random effects model >0.1, a pooled population <1000, and higher between-study heterogeneity I2≥50% are met. |

**Related reference**

(1) Luo M, Cao Q, Zhao Z, et al. Risk factors of epidural hematoma in patients undergoing spinal surgery: a meta-analysis of 29 cohort studies. Int J Surg. 2023;109(10):3147-3158. Published 2023 Oct 1. doi:10.1097/JS9.0000000000000538
(2) Mei Z, Wang Q, Zhang Y, et al. Risk Factors for Recurrence after anal fistula surgery: A meta-analysis. Int J Surg. 2019;69:153-164. doi:10.1016/j.ijsu.2019.08.003
